# The feasibility of finger prick autologous blood (FAB) as a novel treatment for severe dry eye disease (DED): protocol for a randomised controlled trial

**DOI:** 10.1136/bmjopen-2018-026770

**Published:** 2018-10-31

**Authors:** Shafi Balal, Arit Udoh, Yannis Pappas, Erica Cook, Garry Barton, Ali Hassan, Karen Hayden, Rupert Richard Alexander Bourne, Sajjad Ahmad, Shahina Pardhan, Michael Harrison, Benjamin Sharma, Mohammad Wasil, Anant Sharma

**Affiliations:** 1 Bedford Hospital NHS Trust, Bedford, UK; 2 Imperial College Healthcare NHS Trust, London, UK; 3 Clinical Trial Unit, School of Medicine, Anglia Ruskin University, Chelmsford, UK; 4 The Research Centre for Health Organisation and Delivery, Institute for Health Research, University of Bedfordshire, Luton, UK; 5 Norwich Medical School, University of East Anglia, Norwich, UK; 6 Moorfields Eye Hospital NHS Foundation Trust, London, UK; 7 Vision and Eye Research Unit (VERU), School of Medicine, Anglia Ruskin University, Cambridge, UK; 8 Ophthalmology, Cambridge University Hospitals, Cambridge, UK

**Keywords:** ophthalmology, orbital and lacrimal disorders, clinical trials

## Abstract

**Introduction:**

Patients with severe dry eye disease (DED) often have limited treatment options with standard non-surgical management focused on the use of artificial tears for lubrication and anti-inflammatory drugs. However, artificial tears do not address the extraordinary complexity of human tears. Crudely, human tears with its vast constituents is essentially filtered blood. Blood and several blood-derived products including autologous serum, have been studied as tear substitutes. This study proposes to test the use of whole, fresh, autologous blood obtained from a finger prick for treatment of severe DED.

**Methods and analysis:**

The research team at the two participating sites will approach patients with severe DED for this study. Recruitment will take place over 12 months and we expect to recruit 60 patients in total. The primary outcome of this feasibility study is to estimate the proportion of eligible patients approached who consent to and comply with study procedures including treatment regimen and completion of required questionnaires. The secondary outcome measures, although not powered for in this feasibility, include corneal inflammation (assessed by the Oxford corneal staining guide), patient pain and symptoms scores (assessed by the Ocular Surface Disease Index Score), and objective signs of DED as indicated by visual acuity (assessed by Schirmer’s test, tear break-up time, lower and/or upper tear meniscus height measurement). Other secondary outcomes include patients’ quality of life (assessed using the validated EQ-5D-5L Questionnaire), cost to the National Health Service (NHS) and patient (assessed via use of NHS services and privately purchased over-the-counter treatment related to DED) and safety measure of pressure within the eye (assessed by the Intraocular Pressure (IOP) Score).

**Ethics and dissemination:**

This protocol and any subsequent amendments, along with any accompanying material provided to the participant in addition to any advertising material used in this trial have been approved by the East of England - Cambridgeshire and Hertfordshire Research Ethics Committee (REC reference: 17/EE/0508). Written approval from the committee was obtained and subsequently submitted to the respective Trust’s Research and Development (R&D) office with final NHS R&D approval obtained. Data obtained from this study will be published in a suitable peer-review journal and will also presented at international ophthalmic conferences including the American Academy of Ophthalmology, the Royal College of Ophthalmology Annual Congress, the Association for Research and Vision and Ophthalmology, and the European Society of Cataract and Refractive Surgery. Information will be provided to patient groups and charities such as the Sjogren’s Society and the Royal National Institute of Blind People. This will also be shared with the study participants as well as with relevant patient groups and charities.

**Trial registration number:**

NCT03395431; Pre-results.

**Strengths and limitations of this Study**

- This single-blind randomised control trial addresses a significant problem by investigating the use of whole, fresh, autologous blood obtained from a finger prick for treatment of severe dry eye disease.
- The study intervention is cheap, simple and with minimal risk of complication.
- The intervention will allow for the delivery of the beneficial components of tears including growth factors and proteins found in whole blood.
- Patients in the intervention arm may experience soreness in fingers that are repeatedly pricked and will be advised to use a different finger for each eye.

## Introduction

Dry eye disease (DED) is an umbrella term encompassing a range of ocular diseases estimated to affect 14% of all adults aged 48–91 years.^1^ If left untreated, DED can lead to severe reduction in the quality of life (QoL) of the patient. It can also cause loss of vision, pain in response to light, painful recurring stabbing sensations and the feeling of grit in the affected eye(s). Studies show that moderate-to-severe DED can be as disruptive to patient QoL as angina.^2 3^ Despite this, only less than 2% of all UK medical research funding is directed at diseases of the eye, leaving eye research critically underfunded.^4^ No curative agents for DED exist. The treatment of the disorder is essentially symptomatic with standard non-surgical treatment focused on the use of artificial tears for lubrication and anti-inflammatory drugs.

Anti-inflammatory agents used in DED include topical steroids and ciclosporine drops but these can cause side effects that limit their long-term use.^5^ Topical steroids can also cause cataracts and glaucoma, all of which further limit their use. Surgical options for DED include punctal occlusion to reduce tear drainage, punctal cauterisation (burning the drainage channel of tears and preventing their outflow) and partial suturing of the eyelids. However, these treatment options are associated with adverse effects and varying levels of patient tolerability. Overall, available conventional treatment options for DED often only alleviate symptoms, have limited effectiveness, and in most cases patients may fail to respond—although the exact rate of treatment failure is unavailable in the published literature.

The mainstay of non-surgical treatment for DED focuses on the use of artificial tears. These can be purchased over the counter and include brand name products such as Viscotears and Optrex.^6^ Human tears contain an extensive range of growth factors, immunoglobulins, enzymes, cytokines, vitamins and electrolytes and these have been shown in numerous studies to be essential for the maintenance and proliferation of corneal epithelial cells as well as for defence against infection.^7^ It is well known that artificial tears fail to account for the extraordinarily complex composition of the natural tear film. Also, many artificial tears contain preservatives that have been shown to adversely affect the cornea.^8^ Crudely, the human tear with its vast constituents is essentially filtered blood and as such is an obvious source for a ‘tear mimic’ containing all tear constituents. Blood and several blood-derived products including autologous serum (AS) have been studied as tear substitute candidates.

AS eye drops have been found to be beneficial in uncontrolled trials by improving the ocular surface and reducing symptoms.^9–11^ However, a Cochrane review concluded that there is inconsistency in evidence on its benefit.^12^ Obtaining AS requires frequent drawing of blood from the patient—a feature that excludes patients with anaemia or heart failure from using AS. Furthermore it also appears that 100% AS is more beneficial than 50% serum and requires larger volumes of blood and/or more frequent venesection.^10^ Patients using AS also require access to a fridge, as the product needs to be stored at low temperatures—a factor that is likely to be inconvenient for patients. In addition, AS is obtained by processing clotted blood at an initial cost of £1653.56 to the National Health Service (NHS), and subsequent 3-monthly costs of £1131.27 per patient.

The relatively high cost represents the biggest hurdle in the use of AS and is often the reason for delay or inaccessibility in its use as a treatment for DED. We propose that finger prick autologous blood (FAB) is a simpler, cost-effective and possibly more acceptable method for treating DED. For this reason, this study proposes to test the use of FAB in which blood obtained from a small finger prick is applied to the eye. Autologous fresh blood is already being used in the subconjunctiva to help heal leaking trabeculectomy blebs.^13–16^ It is also used to help attach limbal autografts in cases of pterygium,^17^ and in vitreoretinal macular hole surgery^18–21^ with no adverse effects reported. The FAB method can be used in patients who are awaiting conventional treatment for autologous blood.

### Rationale and risks/benefits

This study proposes to test the feasibility of the use of whole, fresh, autologous blood as a treatment for severe DED (detailed study protocol presented as online supplementary file). The blood can be obtained from a clean finger, pricked by the patient using a diabetic lancet and administered immediately to the eye. This allows the delivery of most of the aforementioned beneficial components of tears and additional growth factors and proteins, fresh and unprocessed, which can help heal the ocular surface. If validated, this may replace current AS practice and with its ease of use, vastly reduced cost and greater convenience may mean that it could be extended to other ocular surface diseases. The proposing team have completed an exploratory study on the use of FAB for persistent epithelial defects and severe DED and preliminary results indicate improvement with no adverse events (AEs) reported.^22^ The exploratory study included 16 patients with a diagnosis of severe-to-moderate dry eye syndrome and used the FAB method for treatment. The findings of the study demonstrated mean improvements in visual acuity, Oxford corneal staining grade, tear break-up time and DED questionnaire score. The response rate from participants was good with only a single patient who met the inclusion criteria not wishing to participate in the trial. Both the amount of staining (indicating inflammation and ocular surface damage) and their DED questionnaire scores (indicating severity of their symptoms and impact on QoL) showed mean improvement which reached statistical significance. A search of clinical databases (Medline, CINAHL and AMED) including ongoing trials on the UKCRN (UK Clinical Research Network) portfolio database and related websites (www.controlled-trials.com) did not identify any studies using FAB as a treatment for DED.

10.1136/bmjopen-2018-026770.supp1 Supplementary data

## Primary and secondary objectives

The primary objective is to determine the feasibility of a definitive randomised controlled trial (RCT) to evaluate effectiveness of the use of fresh autologous blood (FAB) compared with conventional treatment for patients with severe dry eye syndrome. The primary outcome is the proportion of eligible patients approached who consent to and comply with study procedure including treatment regimen and completion of required questionnaires. The secondary objectives, although not powered for within this feasibility include: to estimate the effectiveness of the trial intervention and further explore the acceptability of the study design, and to explore the feasibility of collecting resource use and QoL data to inform the design of the health economics component of a future definitive trial. The secondary outcome measures, although not powered for within this feasibility, include corneal inflammation (assessed by the Oxford Corneal Staining Guide), patient pain and symptoms scores (assessed by the Ocular Surface Disease Index (OSDI) Score) and objective signs of DED as indicated by visual acuity (assessed by Schirmer’s test, tear break-up time, lower and/or upper tear meniscus height measurement). Other secondary outcomes include patients’ QoL (assessed using the validated EQ-5D-5L Questionnaire), cost to the NHS and patient (assessed via use of NHS services and privately purchased over-the-counter treatment related to dry eye disease) and safety measure of pressure within the eye (assessed by the Intraocular Pressure (IOP) Score).

## Method

### Study design

**Figure 1:**
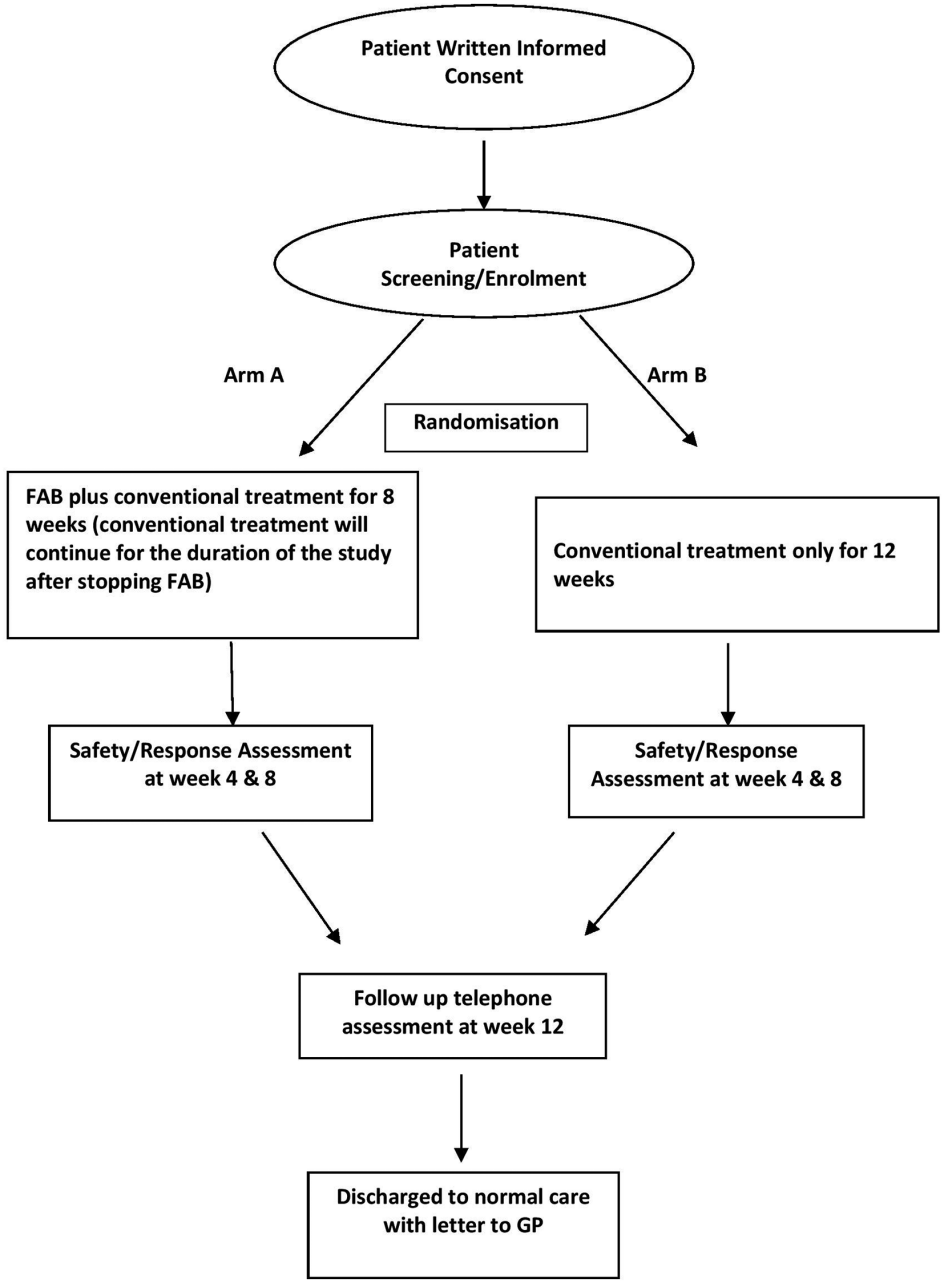
Study scheme. FAB, fresh autologous blood.

This is a single blind, two-arm feasibility RCT of FAB with conventional treatment versus conventional therapy alone for severe DED, including a qualitative process evaluation (figure 1 provides details of the study scheme). This trial is ongoing and open to recruitment at two participating sites: Bedford Hospital NHS Trust and Moorfields Eye Hospital London. Recruitment into the trial commenced in April 2018 and is due to finish by April 2019.

### Sample size

As this is a feasibility trial, there were no formal sample size calculations.^23^ The aim of the study is to recruit a sufficient number of patients to evaluate the acceptability and feasibility of the intervention. An outcome of this study will be to estimate parameters such as the SD for a sample size calculation of a subsequent full-scale trial. A sample of 52 patients (26 in each arm) is considered adequate for obtaining reliable sample size estimates.^24 25^ To allow for a conservative attrition rate of 10%, we aim to recruit 60 patients into the trial.

### Participant selection

#### Inclusion criteria

- Patient age ≥18 years.
- Severe symptomatic DED diagnosed by: OSDI Score >33; or Oxford corneal staining grade ≥2; or Schirmer’s test without anaesthesia <5 mm at 5 min.
- Patients on artificial tears and/or lubricating drops/gel four times a day.
- Patient able to give consent.
- Patients able and willing to complete the QoL Questionnaires required for the study.

#### Exclusion criteria

- Fear of needles.
- Unable or not willing to carry out repeat finger pricks.
- Patients with infected finger/s or systemic infection or on systemic antibiotics for infection.
- Patients with active ocular infection, active immunological corneal melt or recurrent corneal erosion.
- Pregnant or breastfeeding women.
- Previous use of FAB treatment (eg, from exploratory study).
- Systemic illness causing immune system deficiency.
- Graft versus host disease.
- Previous use of AS within 3 months.
- Diabetes.

### Intervention

#### Arm A—FAB plus conventional treatment

The patients will use FAB therapy alongside conventional therapy (artificial tears, ciclosporine drops or punctal plugs/cautery) as recommended by their treating ophthalmologist. Hands will be washed with soap and water, dried and then a fingertip of the hand will be wiped with an alcohol-based wipe and self-pricked using a standard diabetic lancet. The drop of blood produced as normal is applied to the lower fornix of the affected eye(s) with the lower lid pulled down slightly by the patient. The blood will be applied four times a day. A fresh finger will be used for each eye and blood wiped away with the alcohol-based wipe. Patients will be advised to keep nails short and to avoid nail varnish. The FAB intervention will be applied at least 15 min after any artificial tears and participants must not use drops for at least half an hour after this.

#### Arm B—conventional treatment only

The patients will use conventional therapy (artificial tears, ciclosporine drops and punctal plugs/cautery) as recommended by their treating ophthalmologist.

### Qualitative approach

A nested qualitative approach using in-depth, semistructured interviews will help us understand the lived experience of people using FAB and factors relating to the ways that clinical departments and healthcare professionals adapt to working with FAB. Topics, questions and probes in the interviews will emerge from three sources: the relevant literature; our experience of and involvement in clinical ophthalmology; and consultation with patients. In this feasibility study, we will use a convenience sampling technique for the qualitative work. We will interview all clinicians who administer the intervention in the study and we will aim to interview 10 patients (adjusted for setting, gender and age group representation). Given the resources, we will aim to draw findings from a larger sample in a future study. This phase will work in parallel to the quantitative data collection so that emerging themes can be investigated in later interviews. Even though the feasibility study sample is reasonably limited, we anticipate some useful insights about the use of FAB because of the triangulation between the RCT and the qualitative findings (for example, reasons for partial adherence).

### Economic evaluation

Good practice recommendations for cost-effectiveness analyses suggest concentrating on the measurement of large cost drivers, with less focus on resources that are not expected to differ between different treatments.^26^ Estimation of cost-effectiveness is therefore an iterative process and by including a health economic component in the feasibility it is possible to consider how the methods might be refined in any future definitive study. In order to estimate costs, informed by previous data collection instruments (Database of Instruments for Resource Use Measurement: http://www.dirum.org/) and National Institute for Health and Care Excellence guidance,^27^ a self-report resource use questionnaire will be devised. This will capture the use of NHS services and privately purchased over-the-counter treatments such as Optrex. For benefits, EQ-5D-5L^28^ will be used to measure QoL, enabling quality-adjusted life year scores to be calculated. The main purpose of the economic analysis is to inform the decision regarding how and what cost and effect data would be collected within a more definitive study. In order to inform this decision, we will estimate completion rates and seek to identify big cost drivers.

### Outcome assessment

The primary outcome measure is the proportion of eligible patients approached who consent to and comply with the trial protocol. This will be assessed by: number of eligible patients within the study population and recruitment time frame, percentage of eligible patients accepted for randomisation, patient compliance with trial protocol measured as per self-reported adherence to intervention and percentage of patients completing study. The secondary outcomes include reduction in corneal inflammation (assessed by the Oxford corneal staining guide), patient pain and symptoms scores (assessed by the OSDI Score) and improvement in objective signs of DED as indicated by visual acuity, Schirmer’s test, tear break-up time, lower and/or upper tear menisci height measurement. Other secondary end points include patients’ QoL (assessed using the EQ-5D-5L Questionnaire), cost to the NHS and patient (assessed via use of additional NHS services and privately purchased over-the-counter treatments related to DED) and safety measure of pressure within the eye (assessed by IOP Score).

### Data analysis

Detailed data validation will examine completeness, existence and accuracy of collected data to assess data quality and identify missing and conflicting data. Statistical analysis will be performed on an intention-to-treat basis ie, inclusion of patients randomly assigned, regardless of adherence, actual treatment received, and subsequent withdrawal of treatment and/or deviation from the protocol and per-protocol (ie, inclusion of those who completed the treatment as planned)^29^ and will be reported according to 2010 CONSORT guidelines.^30^ All statistical analyses will be completed using SPSS Statistics V.22.0.^31^ A value of p<0.05 will be accepted as statistically significant. Conforming to recommendations for feasibility studies, data analysis will be primarily descriptive, with means, SD and frequency counts calculated for all variables of interest.

Exploratory efficacy analysis will compare the primary outcome variables derived from the data collected at 8 weeks between the two arms using a marginal general linear model with robust standard errors, to allow for clustering by group. Secondary outcomes between the intervention and control groups will be compared at 4 weeks, 8 weeks and 12 weeks (4 weeks post-treatment). Safety outcome measures will include IOP rise and any reported infection. Secondary analyses of the primary outcome, controlling for baseline covariates, will be performed using proportional hazards regression models.^32^ Analysis of treatment discontinuation adjusted for clinical site, baseline status, and whether the patient is randomised to the FAB or control (conventional treatment only) group will be conducted. Any interaction between baseline status or regimen and treatment will also be assessed. Interactions between significant covariates and treatment effects will be assessed in the context of the proportional hazards models.

Interviews will be recorded, transcribed and analysed using the framework analysis. We will structure the analysis of collected data to fit two evaluation frameworks relevant to the successful implementation of FAB:

- Patient-oriented: Understand the lived experience of patients using FAB with emphasis on intervention acceptability, perceived enablers and barriers for adherence, perceived clarity of advice, and guidance and factors relating to the initial uptake of the intervention.
- Organisation-oriented: Whether and how the use of FAB affected workload and/or workflow; identify process change, adaptation challenges, skills gap; establish treatment fidelity and clinician acceptability.

### Study procedures

#### Informed consent procedures

It is the responsibility of the investigator, or appropriate Good Clinical Practice (GCP) trained person delegated by the investigator as documented in the site delegation log, to obtain written informed consent from each participant prior to any participation/study-specific procedures. This should follow adequate explanation of the aims, methods, anticipated benefits and potential hazards of the study. The participants will be given ample time to consider giving their consent for the study. For this study a minimum of 24 hours will be given during which the consenting team will be contactable over the phone to answer any questions. The date on which the participant information sheet (PIS) is given to the participant must be documented within the patient’s notes to confirm that sufficient time was given. The investigator (or other qualified person) will explain to the potential participant that they are free to refuse any involvement with the study or alternatively withdraw their consent at any point during the study and for any reason. If there is any further safety information which may result in significant changes in the risk/benefit analysis, the PIS and informed consent form (ICF) will be reviewed and updated accordingly. All participants that are actively enrolled on the study will be informed of the updated information and given a revised copy of the PIS/ICF in order to confirm their wish to continue on the study.

#### Screening procedures

Patients will have undergone the below procedures as per standard of care which will in turn feed in to the investigator for their review prior to approaching the potential participant.

- OSDI Score >33; or
- Oxford corneal staining grade ≥2; or
- Schirmer’s test without anaesthesia <5 mm at 5 min.

The results from these will be reviewed by the investigator prior to approaching the potential participant.

#### Randomisation procedures

Randomisation will be carried out by the Anglia Ruskin Clinical Trials Unit (ARCTU) using the SEALED ENVELOPE randomisation service. SEALED ENVELOPE is a randomisation and online database service developed for clinical trial services. It is an internet-based system and will be set for this study by ARCTU in accordance with the protocol. Enrolled patients will undergo 1:1 block randomisation to Arm A or Arm B. The system stores the predetermined sequence of randomisation and this list is visible to neither the investigator nor the ARCTU staff. Once a patient has consented to take part in the trial, they will be randomly allocated either to Arm A to receive FAB and conventional therapy or to Arm B to receive conventional therapy only. The research nurse or fellow or designated staff will log on to a web browser application and enter the patient’s eligibility factors into the system. The treatment allocation will be sent to the research team who will make the necessary arrangements for the patient’s treatment plan.

The treating ophthalmologist in the clinic will prescribe and counsel all patients on the correct technique for conventional treatment. The unblinded research nurses will provide additional training to the patients on the method of delivering FAB, including the use of a training video. Patients will be advised by the research team to inform their ophthalmologist if they have been started on any new treatment during the trial period by other clinicians or themselves.

### End of study definition

The definition of the end of study will be the point where the last patient recruited had the last follow-up visit.

### Participant withdrawal

Patients will be withdrawn from study based on the following:

- New diagnosis of infection on finger or eye.
- Requesting to be withdrawn for other reasons such as inconvenience of increased clinic visits.
- Pregnancy after recruitment.
- Systemic infection, or systemic antimicrobials for infection.
- If finger sore from repeated prick and patient does not want to use another finger.

Any adverse effects will be reported back to the lead investigator at the site and, if necessary, the patients.

### Notification and reporting AEs or reactions

No serious AEs (SAEs) or complications associated with use of FAB in the treatment of ocular disease has been reported by the literature. Preliminary results of a pilot study conducted by the authors and involving 16 patients with DED also demonstrated no adverse effects of using FAB in the eye for 2 months.^22^ Although, we do not envisage any SAEs with the intervention in this trial, if an AE occurs and is not defined as SERIOUS, the AE will be recorded in the study file and the participant followed up by the research team. The AE will also be documented in the participants’ medical notes (where appropriate) and the case report form (CRF). SAEs that are considered to be ‘related’ and ‘unexpected’ will be reported to the sponsor within 24 hours of learning of the event and to the main research ethics committee within 15 days in line with the required time frame. SAE reporting will commence at the start of the trial and up to 4 weeks after the intervention.

### Patient and public involvement

Patients who participated in a previous pilot (exploratory) study conducted by the study authors^22^ were involved in a focus group meeting and provided inputs on research design and study procedures. Two of the patients also participated in an advisory group for this trial and provided input on prioritising the research questions and also reviewed the study protocol and participant information resources. Patients in this trial will be invited to request study results from the research team if interested.

## Ethics and dissemination

Participant anonymity will be protected and maintained at all times. Information with regards to study participants will be kept confidential and managed in accordance with the Data Protection Act, NHS Caldicott Guardian, The Research Governance Framework for Health and Social Care and Research Ethics Committee Approval in the UK. Data obtained in the trial will be stored using a unique study code, which is non-identifiable and anonymised. Patients' personal data will be stored separately and confidentially by the participating sites and will not be entered into the trial database. All written information will stored in a locked filing cabinet in a locked room. Any web-based data will be stored in a secure password-protected and encrypted central database at ARCTU. Only individuals directly involved with the study will have access to trial data. Study participants will also be provided with the contact details of the trial team at the respective sites who will be able to answer any additional queries.

## Supplementary Material

Reviewer comments

Author's manuscript
