# Supplementary material for: The feasibility of finger prick autologous blood (FAB) as a novel treatment for severe dry eye disease (DED): protocol for a randomised controlled trial
Source: BMJ Open. 2018 Oct 31;8(10):e026770. doi: 10.1136/bmjopen-2018-026770 (PMC6252631; doi:10.1136/bmjopen-2018-026770)
Supplement: Author's manuscript [file bmjopen-2018-026770.draft_revisions.pdf]

BMJ Open is committed to open peer review. As part of this commitment we make the peer review history of every article we publish publicly available.

When an article is published we post the peer reviewers' comments and the authors' responses online. We also post the versions of the paper that were used during peer review. These are the versions that the peer review comments apply to.

The versions of the paper that follow are the versions that were submitted during the peer review process. They are not the versions of record or the final published versions. They should not be cited or distributed as the published version of this manuscript.

BMJ Open is an open access journal and the full, final, typeset and author-corrected version of record of the manuscript is available on our site with no access controls, subscription charges or pay-per-view fees (http://bmjopen.bmj.com).

If you have any questions on BMJ Open's open peer review process please email info.bmjopen@bmj.com

BMJ Open

The Feasibility of Fingerprick Autologous Blood (FAB) As a Novel Treatment for Severe Dry Eye Disease (DED): Protocol for a Randomised Controlled Trial

Journal:	*BMJ Open*
Manuscript ID	bmjopen-2018-026770
Article Type:	Protocol
Date Submitted by the Author:	18-Sep-2018

Complete List of Authors:	Balal, Shafi ; Bedford Hospital NHS Trust; Imperial College Healthcare NHS Trust<br>Udoh, Arit; Anglia Ruskin University, Clinical Trial Unit<br>Pappas, Yannis; The University of Bedfordshire,<br>Cook, Erica; University of Bedfordshire, Department of Psychology<br>Barton, Garry; University of East Anglia, Norwich Medical School<br>Hassan, Ali ; Bedford Hospital NHS Trust; Moorfields Eye Hospital NHS Foundation Trust<br>Hayden , Karen ; Anglia Ruskin University, Clinical Trial Unit, School of Medicine<br>Bourne, Rupert; Anglia Ruskin University, Vision and Eye Research Unit; Huntingdon Glaucoma Diagnostic & Research Centre at Hinchingbrooke Hospital,<br>Ahmad , Sajjad ; Moorfields Eye Hospital NHS Foundation Trust<br>Pardhan, Shahina ; Anglia Ruskin University, Vision and Eye Research Unit (VERU), School of Medicine<br>Harrison , Michael ; Anglia Ruskin University<br>Sharma, Benjamin ; Bedford Hospital NHS Trust<br>Wasil , Mohammad ; Bedford Hospital NHS Trust<br>Sharma, Anant ; Bedford Hospital NHS Trust; Moorfields Eye Hospital NHS Foundation Trust

Keywords:	OPHTHALMOLOGY, Orbital and lacrimal disorders < OPHTHALMOLOGY, Clinical trials < THERAPEUTICS

SCHOLARONE™
Manuscripts

The Feasibility of Fingerprick Autologous Blood (FAB) As a Novel Treatment for Severe Dry Eye Disease (DED): Protocol for a Randomised Controlled Trial

Authors:

Shafi Balal[1,2]
Principal Investigator
[1]Bedford Hospital NHS Trust, Bedford, United Kingdom, and
[2]Imperial College Healthcare NHS Trust, London, United Kingdom
Email: shafi.balal@nhs.net

***Arit Udoh**[3]
Trial Coordinator
[3]Clinical Trial Unit, School of Medicine, Anglia Ruskin University, Chelmsford, United Kingdom
Email: arctu@anglia.ac.uk
Phone: 0845 196 4938

Yannis Pappas[4]
Qualitative Lead
[4]The Research Centre for Health Organisation and Delivery, Institute for Health Research, University of Bedfordshire, Luton, United Kingdom
Email: Yannis.Pappas@beds.ac.uk

Erica Cook[4]
Trial Statistician
[4]The Research Centre for Health Organisation and Delivery, Institute for Health Research, University of Bedfordshire, Luton, United Kingdom
Email: erica.cook@beds.ac.uk

Garry Barton[5]
Health Economist
[5]Norwich Medical School, University of East Anglia, Norwich, United Kingdom
Email: G.Barton@uea.ac.uk

Ali Hassan[1,6]
Research Fellow
[1]Bedford Hospital NHS Trust, Bedford, United Kingdom, and
[6]Moorfields Eye Hospital NHS Foundation Trust, London, United Kingdom
Email: Ali.Hassan@bedfordhospital.nhs.uk

Karen Hayden[3]
Clinical Trial Unit Interim Director
[3]Clinical Trial Unit, School of Medicine, Anglia Ruskin University, Chelmsford, United Kingdom
Email: karen.hayden@anglia.ac.uk

Rupert Bourne[7]

For peer review only

For peer review only - http://bmjopen.bmj.com/site/about/guidelines.xhtml

Co-Investigator
[7]Vision and Eye Research Unit (VERU), School of Medicine, Anglia Ruskin University, Cambridge, United Kingdom.
Email: rb@rupertbourne.co.uk

Sajjad Ahmad[6]
Principal Investigator
[6]Moorfields Eye Hospital NHS Foundation Trust, London, United Kingdom
Email: Sajjad.Ahmad@moorfields.nhs.uk

Shahina Pardhan[7]
Academic Lead
[7]Vision and Eye Research Unit (VERU), School of Medicine, Anglia Ruskin University, Cambridge, United Kingdom.
Email: Shahina.Pardhan@anglia.ac.uk

Michael Harrison[3]
Co-investigator
[3]Clinical Trial Unit, School of Medicine, Anglia Ruskin University, Chelmsford, United Kingdom
Email: Michael.harrisson@anglia.ac.uk

Benjamin Sharma[1]
Trial Assistant
[1]Bedford Hospital NHS Trust, Bedford, United Kingdom
Email: ruben.sharma12@gmail.com

Mohammad Wasil
Assistant Director, RDI
[1]Bedford Hospital NHS Trust, Bedford, United Kingdom
Email: mohammad.wasil@bedfordhospital.nhs.uk

Anant Sharma[1,6].
Chief Investigator
[1]Bedford Hospital NHS Trust, Bedford, United Kingdom, and
[6]Moorfields Eye Hospital NHS Foundation Trust, London, United Kingdom
Email: Anant.Sharma@bedfordhospital.nhs.uk

Trial Sponsor: Bedford Hospital NHS Trust, Bedford, United Kingdom
Word count: 3828
***Corresponding author: arctu@anglia.ac.uk**

For peer review only

For peer review only - http://bmjopen.bmj.com/site/about/guidelines.xhtml

ABSTRACT

Introduction

Patients with severe dry eye disease often have limited treatment options with standard non-surgical management focused on the use of artificial tears for lubrication and anti-inflammatory drugs. However, artificial tears do not address the extraordinary complexity of human tears. Crudely, human tears with its vast constituents is essentially filtered blood. Blood and several blood-derived products including autologous serum, have been studied as tears substitutes. This study proposes to test the use of whole, fresh, autologous blood obtained from a fingerprick for treatment of severe dry eye disease.

Methods and Analysis

The research team at the two participating sites will approach patients with severe dry eye disease for this study. Recruitment will take place over 12 months and we expect to recruit 60 patients in total. The primary outcome of this feasibility study is to estimate the proportion of eligible patients approached who consent to and comply with study procedures including treatment regimen and completion of required questionnaires. The secondary outcome measures, although not powered for in this feasibility, include corneal inflammation (assessed by Oxford Corneal Staining Guide), patient pain and symptoms scores (assessed by Ocular Surface Disease Index (OSDI) score), and objective signs of dry eye disease as indicated by visual acuity (assessed by Schirmer's test, tear breakup time, lower and/or upper tear meniscus height measurement). Other secondary outcomes include patients' quality of life (assessed using the validated EQ-5D-5L questionnaire), cost to the NHS and patient (assessed via use of NHS services and privately purchased over the counter treatment related to dry eyes disease) and safety measure of pressure within the eye (assessed by intra ocular pressure (IOP) score).

Ethics and Dissemination

This trial is ongoing and received a favourable research ethics opinion from the East of England - Cambridgeshire and Hertfordshire Research Ethics Committee (REC reference: 17/EE/0508).

For peer review only - http://bmjopen.bmj.com/site/about/guidelines.xhtml

Trial Registration Number: NCT03395431

Trial Sponsor: Bedford Hospital NHS Trust, Bedford, United Kingdom

Keywords: ophthalmology, orbital and lacrimal disorder, clinical trials

Strength and Limitation of Study

- This study proposes to test the feasibility of the use of whole, fresh, autologous blood obtained from a fingerprick for treatment of severe dry eye disease.
- The use of fingerpick autologous blood will allow for the delivery of beneficial components of tears including growth factors and proteins that can help heal the ocular surface.
- If validated, fingerpick autologous blood may replace the use of autologous serum and its vastly reduced cost with greater convenience may mean that its use can be extended to other ocular surface diseases.
- Patients in the intervention arm may experience soreness in fingers that are repeatedly pricked.
- To minimise risk of finger soreness, patients will be advised to use a different finger for each eye.

For peer review only

For peer review only - http://bmjopen.bmj.com/site/about/guidelines.xhtml

INTRODUCTION

Dry eye disease (DED) is an umbrella term encompassing a range of ocular diseases estimated to affect 14% of all adults aged 48 to 91 (1). If left untreated, DED can lead to severe reduction in the quality of life of the sufferer. It can also cause loss of vision, pain in response to light, painful recurring stabbing sensations, and the feeling of grit in the affected eye(s). Studies show that moderate to severe DED can be as disruptive to patient quality of life as angina (2,3). Despite this, only less than 2% of all United Kingdom (UK) medical research funding is directed at diseases of the eye, leaving eye research critically underfunded (4). No curative agents for DED exist. The treatment of the disorder is essentially symptomatic with standard non-surgical treatment focused on the use of artificial tears for lubrication and anti-inflammatory drugs.

Anti-inflammatory agents used in DED include topical steroids and cyclosporine drops but these can cause side effects that limit their long term use (5). Topical steroids can also cause cataracts and glaucoma, all of which further limit their use. Surgical options for DED include punctal occlusion to reduce tear drainage, punctal cauterization (burning the drainage channel of tears and preventing their outflow) and partial suturing of the eyelids. However, these treatment options are associated with adverse effects and varying levels of patient tolerability. Overall, available conventional treatment options for DED often only alleviate symptoms, have limited effectiveness, and in most cases patients may fail to respond— although the exact rate of treatment failure is unavailable in the published literature.

The mainstay of non-surgical treatment for DED focuses on the use of artificial tears. These can be purchased over the counter and include brand name products such as Viscotears® and Optrex® (6). Human tears contains an extensive range of growth factors, immunoglobulins, enzymes, cytokines, vitamins and electrolytes and these have been shown in numerous studies to be essential for the maintenance and proliferation of corneal epithelial cells as well as for defence against infection (7). It is well known that artificial tears fail to account for the extraordinarily complex composition of the natural tear film. Also, many artificial tears contain preservatives that have been shown to adversely affect the cornea (8). Crudely, human tears with its vast constituents is essentially filtered blood and as such is an obvious source for a “tear mimic” containing all tear constituents. Blood and several blood-derived products including autologous serum have been studied as tears substitute candidates.

For peer review only - http://bmjopen.bmj.com/site/about/guidelines.xhtml

Autologous serum (AS) eye drops have been found in uncontrolled trials to be beneficial by improving the ocular surface and reducing symptoms (9–11). However, a Cochrane review concluded that there is inconsistency in evidence on its benefit (12). Obtaining autologous serum requires frequent drawing of blood from the patient— a feature that excludes patients with anaemia or heart failure from using AS. Furthermore it also appears that 100% autologous serum is more beneficial than 50% serum and requires larger volumes of blood and/or more frequent venesection (10). Patients using AS also require access to a fridge, as the product needs to be stored at low temperatures; a factor that is likely to be inconvenient for patients. In addition, AS is obtained by processing clotted blood with the initial cost to the NHS of £1653.56, and subsequent three-monthly cost of £1131.27 per patient.

The relatively high cost represents the biggest hurdle in the use of AS and is often the reason for delay or inaccessibility in its use as a treatment for DED..We propose that finger prick autologous blood is a simpler, cost-effective and possibly more acceptable method for treating dry eye disease. For this reason, this study proposes to test the use of fingerprick autologous blood (FAB) in which blood obtained from a small finger-prick is applied to the eye. Autologous fresh blood is already used in the sub-conjunctiva to help heal leaking trabeculectomy blebs (13–16). It is also used to help attach limbal autografts in cases of pterygium (17), and in vitreoretinal macular hole surgery (18–21) with no adverse effects reported. The FAB method can be used in patients who are awaiting conventional treatment for autologous blood.

For peer review only

Rationale and Risks/Benefits

This study proposes to test the feasibility of the use of whole, fresh, autologous blood as a treatment for severe dry eye disease. The blood can be obtained from a clean finger, pricked by the patient using a diabetic lancet and administered immediately to the eye. This allows the delivery of not only most of the aforementioned beneficial components of tears but additional growth factors and proteins, fresh and unprocessed, which can help heal the ocular surface. If validated, this may replace current autologous serum practice and its ease of use, vastly reduced cost with greater convenience may mean that it could be extended to other ocular surface diseases. The proposing team have completed an exploratory study on the use of fingerprick autologous blood (FAB) for persistent epithelial defects and severe dry eye disease and preliminary results indicate improvement with no adverse events reported (22).

For peer review only - http://bmjopen.bmj.com/site/about/guidelines.xhtml

The exploratory study included 16 patients with a diagnosis of severe to moderate dry eye syndrome and used the FAB method for treatment. The findings of the study demonstrated mean improvements in visual acuity, Oxford corneal staining grade, tear breakup time, Schirmer's test and dry eye disease questionnaire score. The response rate from participants was good with only a single patient who met the inclusion criteria not wishing to participate in the trial. Both the amount of staining (indicating inflammation and ocular surface damage) and their DED questionnaire scores (indicating severity of their symptoms and impact on quality of life) showed mean improvement which reached statistical significance. A search of clinical databases (Medline, CINAHL and AMED) including on-going trials on the UK CRN portfolio database and related websites (www.controlled-trials.com) did not identified any studies using FAB as a treatment for dry eye disease.

PRIMARY AND SECONDARY OBJECTIVES

The primary objective is to determine the feasibility of a definitive randomised controlled trial (RCT) to evaluate effectiveness of the use of fresh autologous blood (FAB) compared to conventional treatment for patients with severe dry eye syndrome. The primary outcome is the proportion of eligible patients approached who consent to and comply with study procedure including treatment regimen and completion of required questionnaires. The secondary objectives, although not powered for within this feasibility include: to estimate the effectiveness of the trial intervention and further explore the acceptability of the study design, and to explore the feasibility of collecting resource use and quality of life data to inform the design of the health economics component of a future definitive trial. The secondary outcome measures, although not powered for within this feasibility, include corneal inflammation (assessed by Oxford Corneal Staining Guide), patient pain and symptoms scores (assessed by Ocular Surface Disease Index (OSDI) score), and objective signs of dry eye disease as indicated by visual acuity (assessed by Schirmer's test, tear breakup time, lower and/or upper tear meniscus height measurement). Other secondary outcomes include patients' quality of life (assessed using the validated EQ-5D-5L questionnaire), cost to the NHS and patient (assessed via use of NHS services and privately purchased over the counter treatment related to dry eyes disease) and safety measure of pressure within the eye (assessed by intra ocular pressure (IOP) score).

For peer review only - http://bmjopen.bmj.com/site/about/guidelines.xhtml

METHOD

Study Design

This is a single blind, two-arm feasibility randomised controlled trial of FAB with conventional treatment versus conventional therapy alone for severe dry eye disease, including a qualitative process evaluation (Figure 1 provides details of the study scheme). This trial is ongoing and open to recruitment at two participating sites: Bedford Hospital NHS Trust and Moorfields Eye Hospital London. Recruitment into the trial commenced in April 2018 and is due to finish by April 2019.

Sample Size

As this is a feasibility trial, there was no formal sample size calculations (23). The aim of the study is to recruit sufficient patients to evaluate the acceptability and feasibility of the intervention. An outcome of this study will be to estimate parameters such as the standard deviation for a sample size calculation of a subsequent full-scale trial. A sample of 52 patients (26 in each arm) is considered adequate for obtaining reliable sample size estimates (24, 25). To allow for a conservative attrition rate of 10%, we aim to recruit 60 patients into the trial.

Participant Selection

Inclusion Criteria

- Patient age ≥ 18 years
- Severe symptomatic dry eye disease diagnosed by: Ocular surface disease index (OSDI) score of greater than 33; OR Oxford Corneal Staining grade 2 or greater; OR Schirmer's without anaesthesia <5mm at 5 minutes
- Patients on artificial tears and/or lubricating drops/gel four times a day
- Patient able to give consent
- Patients able and willing to complete the quality of life (QoL) questionnaires required for the study.

Exclusion Criteria

- Fear of needles
- Unable or not willing to carry out repeat finger pricks
- Patients with infected finger/s or systemic infection or on systemic antibiotics for infection.
- Patients with active ocular infection, active immunological corneal melt, or recurrent corneal erosion.

For peer review only - http://bmjopen.bmj.com/site/about/guidelines.xhtml

- Pregnant or breast feeding women
- Previous use of FAB treatment (e.g. from exploratory study)
- Systemic illness causing immune system deficiency
- Graft versus host disease
- Previous use of autologous serum within 3 months
- Diabetes

Intervention

- ***Arm A – FAB plus conventional treatment***

The patients will use FAB therapy alongside conventional therapy (artificial tears, cyclosporine drops or punctal plugs/cautery) as recommended by their treating ophthalmologist. A fingertip of the hand will be wiped with an alcohol steret and self-pricked using a standard diabetic lancet. The drop of blood is produced as normal and applied to the lower fornix of the affected eye(s) with the lower lid pulled down slightly by the patient. The blood will be applied four times a day. A fresh finger will be used for each eye and blood wiped away with the alcohol steret. Patients will be advised to keep nails short and nail varnish avoided. FAB will be applied at least 15 minutes after any artificial tears and no drops for at least half an hour afterwards.

- ***Arm B – Conventional treatment only***

The patients will use conventional therapy (artificial tears, cyclosporin drops and punctal plugs/cautery) as recommended by their treating ophthalmologist.

Qualitative Approach

A nested qualitative approach using in-depth, semi-structured interviews will help us understand the lived experience of people using FAB and factors relating to the ways that clinical departments and healthcare professionals adapt to working with FAB. Topics, questions and probes in the interviews will emerge from three sources: the relevant literature; our experience of and involvement in clinical ophthalmology; and consultation with patients. In this feasibility study, we will use a convenience sampling technique for the qualitative work. We will interview all clinicians who administer the intervention in the study and we will aim to interview 10 patients (adjusted for setting, gender and age group representation). Given the resources, we will aim to draw findings from a larger sample in a future study. This phase will work in parallel to the quantitative data collection so that emerging themes can be investigated in later interviews. Even though the feasibility study sample is reasonably

For peer review only - http://bmjopen.bmj.com/site/about/guidelines.xhtml

limited, we anticipate some useful insights about the use of FAB to occur because of the triangulation between the RCT and the qualitative findings (for example, reasons for partial adherence).

Economic Evaluation

Good practice recommendations for cost-effectiveness analyses suggest concentrating on the measurement of large cost drivers, with less focus on resources that are not expected to differ between different treatments (26). Estimation of cost-effectiveness is therefore an iterative process and by including a health economic component in a feasibility it is possible to consider how the methods might be refined in any future definitive study. In order to estimate costs, informed by previous data collection instruments (Database of Instruments for Resource Use Measurement: http://www.dirum.org/) and NICE guidance (27), a self-report resource use questionnaire will be devised. This will capture the use of NHS services and privately purchased over the counter treatments such as OPTREX. For benefits, the EQ-5D-5L (28) will be used to measure quality of life, enabling QALY (Quality Adjusted Life Year) scores to be calculated. The main purpose of the economic analysis is to inform the decision regarding how and what cost and effect data would be collected within a more definitive study. In order to inform this decision, we will estimate completion rates and seek to identify big cost drivers.

Outcome Assessment

The primary outcome measure is the proportion of eligible patients approached who consent to and comply with the trial protocol. This will be assessed by: number of eligible patients within the study population and recruitment timeframe, percentage of eligible patients accepted for randomisation, patient compliance with trial protocol measured as per self-reported adherence to intervention, and percentage of patients completing study. The secondary outcomes include reduction in corneal inflammation (assessed by Oxford Corneal Staining Guide), patient pain and symptoms scores (assessed by Ocular Surface Disease Index (OSDI) score), improvement in objective signs of dry eye disease as indicated by visual acuity (assessed by Schirmer's test, tear breakup time, lower and/or upper tear menisci height measurement). Other secondary endpoints include patients' quality of life (assessed using the EQ-5D-5L questionnaire), cost to the NHS and patient (assessed via use of additional NHS services and privately purchased over the counter treatments related to dry

For peer review only - http://bmjopen.bmj.com/site/about/guidelines.xhtml

eyes disease) and safety measure of pressure within the eye (assessed by Intra ocular pressure (IOP) score).

Data Analysis

A detailed data validation will examine completeness, existence and accuracy of collected data to assess data quality and identify missing and conflicting data. The statistical analysis will be performed on an intention-to-treat basis (i.e. inclusion of patients randomly assigned, regardless of adherence, actual treatment received, and subsequent withdrawal of treatment and/or deviation from the protocol and per-protocol (i.e. inclusion of those who completed the treatment as planned) (29) and will be reported according to 2010 CONSORT guidelines (30). All statistical analyses will be completed using SPSS Statistics v.22.0 (31). A p-value of <0.05 will be accepted as statistical significance. Conforming to recommendations for feasibility studies, data analysis will be primarily descriptive, with means, standard deviations and frequency counts calculated for all variables of interest.

Exploratory efficacy analysis will compare the primary outcome variables derived from the data collected at 8 weeks between the two arms using a marginal general linear model (GLM) with robust standard errors, to allow for clustering by group. Secondary outcomes between the intervention and control groups will be compared at 4 weeks, 8 weeks and 12 weeks (4 weeks post treatment). Safety outcome measures will include IOP rise and any reported infection. Secondary analyses of the primary outcome, controlling for baseline covariates, will be performed using proportional hazards regression models (32). Analysis of treatment discontinuation adjusted for clinical site, baseline status, and whether the patient is randomized to the FAB or control (conventional treatment only) group will be conducted. Any interaction between baseline status or regimen and treatment will also be assessed. Interactions between significant covariates and treatment effects will be assessed in the context of the proportional hazards models.

Interviews will be recorded, transcribed and analysed using the framework analysis. We will structure the analysis of collected data to fit two evaluation frameworks relevant to the successful implementation of FAB:

- Patient-oriented: Understand the lived experience of patients using FAB with emphasis on intervention acceptability, perceived enablers and barriers for adherence,

For peer review only - http://bmjopen.bmj.com/site/about/guidelines.xhtml

perceived clarity of advice, and guidance and factors relating to the initial uptake of the intervention.

- Organisation-oriented: Whether and how the use of FAB affected workload and/or workflow; identify process change, adaptation challenges, skills gap; establish treatment fidelity and clinician acceptability.

Study Procedures

Informed Consent Procedures

It is the responsibility of the Investigator, or appropriately GCP trained person delegated by the Investigator as documented in the site delegation log, to obtain written informed consent from each participant prior to any participation/study specific procedures. This should follow adequate explanation of the aims, methods, anticipated benefits and potential hazards of the study. The participants will be given ample time to consider giving their consent for the study. For this study a minimum of 24 hours will be given during which the consenting team will be contactable over the phone to answer any questions. The date that the Participant Information Sheet (PIS) is given to the participant must be documented within the patient's notes to confirm that sufficient time was given. The Investigator (or other qualified person) will explain to the potential participant that they are free to refuse any involvement within the study or alternatively withdraw their consent at any point during the study and for any reason. If there is any further safety information which may result in significant changes in the risk/benefit analysis, the PIS and Informed Consent Form (ICF) will be reviewed and updated accordingly. All participants that are actively enrolled on the study will be informed of the updated information and given a revised copy of the PIS/ICF in order to confirm their wish to continue on the study.

Screening Procedures

Patients will have undergone the below procedures as per standard of care which will in turn feed in to the investigator for their review prior to approaching the potential participant.

- Ocular surface disease index (OSDI) score of greater than 33; OR
- Oxford Corneal Staining grade 2 or greater; OR
- Schirmer's without anaesthesia <5mm at 5 minutes

The results of these which will be reviewed by investigator prior to approaching the potential participant.

For peer review only - http://bmjopen.bmj.com/site/about/guidelines.xhtml

Randomisation Procedures

Randomisation will be carried out by the Anglia Ruskin Clinical Trials Unit (ARCTU) using the SEALED ENVELOPE randomisation service. SEALED ENVELOPE is a randomisation and online database service developed for clinical trial services. It is an internet based system and will be set for this study by ARCTU in accordance with the protocol. Enrolled patients will undergo 1:1 block randomisation to Arm A or B. The system stores the pre-determined sequence of randomisation and this list is visible to neither the investigator nor ARCTU staff. Once a patient has consented to take part in the trial, they will be randomly allocated to either Arm A to receive FAB and convention therapy or Arm B to receive conventional therapy only. The Research Nurse or Fellow or designated staff will log on to a web browser application and enter the patient's eligibility factors into the system. The treatment allocation will be sent to the research team who will make the necessary arrangements for the patient's treatment plan.

The treating ophthalmologist in clinic will prescribe and counsel all patients on the correct technique for conventional treatment. The unblinded research nurses will provide additional training to the patients on the method of delivering FAB, including the use of a training video. Patients will be advised by the research team to inform their ophthalmologist if they have been started on any new treatment during the trial period by other clinicians or themselves.

End of Study Definition

The definition of the end of study will be the point where the last patient recruited had the last follow-up visit.

Participant Withdrawal

Patients will be withdrawn from study based on the following:

- New diagnosis of infection on finger or eye
- Requesting to be withdrawn for other reasons such as inconvenience of increased clinic visits
- Pregnancy after recruitment
- Systemic infection, or systemic antimicrobials for infection.
- If finger sore from repeated prick and patient does not want to use another finger.

Any adverse effects will be reported back to the lead investigator at the site and if necessary patients

For peer review only - http://bmjopen.bmj.com/site/about/guidelines.xhtml

Notification and Reporting Adverse Events or Reactions

No serious adverse events (SAEs) or complications associated with use of fingerprick autologous blood in the treatment of ocular disease has been reported by the literature. Preliminary results of a pilot study conducted by the authors and involving 16 patients with dry eye disease also demonstrated no adverse effects of using FAB in the eye for two months (22). Although, we do not envisage any serious adverse events with the intervention in this trial, if an adverse event (AE) occurs and is not defined as SERIOUS, the AE will be recorded in the study file and the participant followed up by the research team. The AE will also be documented in the participants' medical notes (where appropriate) and the CRF. Serious Adverse Event (SAEs) that are considered to be 'related' and 'unexpected' will be reported to the sponsor within 24 hours of learning of the event and to the Main REC within 15 days in line with the required timeframe. SAE reporting will commence at the start of the trial and up to 4 weeks after the intervention.

PATIENT AND PUBLIC INVOLVEMENT

Patients who participated in a previous pilot (exploratory) study conducted by the study authors (22) were involved in a focus group meeting and provided inputs on research design and study procedures. Two of the patients also participated in an advisory group for this trial provided input on prioritising the research questions and also reviewed the study protocol and participant information resources. Patients in this trial will be invited to request study results from the research team if interested.

ETHICS AND DISSEMINATION

Participant anonymity will be protected and maintained at all times. Information with regards to study participants will be kept confidential and managed in accordance with the Data Protection Act, NHS Caldicott Guardian, The Research Governance Framework for Health and Social Care and Research Ethics Committee Approval in United Kingdom. Data obtained in the trial will be stored using a unique study code, which is non-identifiable and anonymised. Patient's personal data will be stored separately and confidentially by the participating sites and not entered into the trial database. All written information will stored in a locked filing cabinet in a locked room. Any web-based data will be stored in a secure password protected and encrypted central database at the Anglia Ruskin Clinical Trials Unit.

For peer review only - http://bmjopen.bmj.com/site/about/guidelines.xhtml

Only individuals directly involved with the study will have access to trial data. This protocol and any subsequent amendments, along with any accompanying material provided to the participant in addition to any advertising material used in this trial have been approved by the East of England - Cambridgeshire and Hertfordshire Research Ethics Committee (REC reference: 17/EE/0508). Written Approval from the Committee was obtained and subsequently submitted to the respective Trust's Research and Development office with final NHS R&D approval obtained. Data obtained from this study will be published in a suitable peer-review journal and also presented at international ophthalmic conferences including American Academy of Ophthalmology, Royal College of Ophthalmology annual congress, Association for Research and Vision and Ophthalmology, and the European Society of Cataract and Refractive Surgery. Information will be provided to patient groups and charities such as the Sjogren's society and the Royal National Institute of Blind People. Study participants will also be provided with the contact details of the trial team at the respective sites who will be able to answer any additional queries.

For peer review only

For peer review only - http://bmjopen.bmj.com/site/about/guidelines.xhtml

REFERENCES

1. Moss SE, Klein R, Klein BK. Prevalence of and risk factors for dry eye syndrome. *Arch Ophthalmol*. 2000 Sep 1;118(9):1264–8.

2. Hom MM, Nguyen A, Bielory L. Allergic conjuctivitis and dry eye syndrome. Ann *Allergy Asthma Immunol Off Publ Am Coll Allergy Asthma Immunol*. 2012;108:163–6.

3. Schiffman RM, Walt JG, Jacobsen G, Doyle JJ, Lebovics G, Sumner W. Utility assessment among patients with dry eye disease. *Ophthalmol*. 2003;110(7):1412–9.

4. National Eye Research Centre. Eye research overview [Internet]. 2016. Available from: https://www.nerc-charity.org.uk/our-eye-research

5. Kymionis GD, Bouzoukis DI, Diakonis VF, Siganos C. Treatment of chronic dry eye: focus on cyclosporine. *Clin Ophthalmol Auckl NZ*. 2008 Dec;2(4):829–36.

6. Diebold Y, Herreras JM, Callejo S, Argueso P, Calonge M. Carbomer- Versus Cellulose-Based Artificial-Tear Formulations: Morphologic and Toxicologic Effects on a Corneal Cell Line. *Cornea*. 1998;17(4):433.

7. Yokoi N, Yamada H, Mizukusa Y, Bron AJ, Tiffany JM, Kato T, et al. Rheology of Tear Film Lipid Layer Spread in Normal and Aqueous Tear–Deficient Dry Eyes. *Invest Ophthalmol Vis Sci.* 2008 Dec 1;49(12):5319–24.

8. Pfister R, Burstein N. The effects of opthalmic drugs, vehicles and preservatives on corneal epithelium: a scanning electron microscope study. *Invest Ophthalmol Vis Sci.* 1976;15(4):246–59.

9. Fox R, Chan R, Michelson J, Belmont J, Michelson P. Beneficial effect of artificial tears made with artificial tears made with autologous serum in patients with keratoconjunctivitis sicca. *Arthritis Rheumatol*. 1984;29:577–83.

10. Poon AC, Geerling G, Dart JKG, Fraenkel GE, Daniels JT. Autologous serum eyedrops for dry eyes and epithelial defects: clinical and in vitro toxicity studies. *Br J Ophthalmol.* 2001 Oct 1;85(10):1188.

11. Tsubota K, Goto E, Fujuta H, Ono M, Inuoue H, Saito I. Treatment of dry eye by autologous serum application in Sjogren's Syndrome. *Br J Ophthalmol.* 1999;83:390–5.

12. Pan Q, Angelina A, Zambrano A, Marrone M, Stark WJ, Heflin T, et al. Autologous serum eye drops for dry eye. Cochrane Database Syst Rev [Internet]. 2013;(8). Available from: http://dx.doi.org/10.1002/14651858.CD009327.pub2

13. Biswas S, Zaheer I, Monsalve B, Diamond JP. Compression sutures with autologous blood injection for leaking trabeculectomy blebs. *Br J Ophthalmol.* 2009 Mar 25;93(4):549.

14. Burnstein A, WuDunn D, Ishii Y, Jonescu-Cuypers C, Cantor LB. Autologous blood injection for late-onset filtering bleb leak. *Am J Ophthalmol*. 2001 Jul 1;132(1):36–40.

For peer review only - http://bmjopen.bmj.com/site/about/guidelines.xhtml

15. Dinah C, Bhachech B, Ghosh G. Long-term success with autologous blood injection for leaking trabeculectomy blebs. *Br J Ophthalmol.* 2010 Mar 9;94(3):392.

16. Haynes WL, Alward WLM. Combination of Autologous Blood Injection and Bleb Compression Sutures to Treat Hypotony Maculopathy. *J Glaucoma* [Internet]. 1999;8(6). Available from: http://journals.lww.com/glaucomajournal/Fulltext/1999/12000/Combination_of_Autologous_Blood_Injection_and_Bleb.8.aspx

17. de Wit D, Athanasiadis I, Sharma A, Moore J. Sutureless and glue-free conjunctival autograft in pterygium surgery: a case series. *Eye*. 2010 Sep;24(9):1474–7.

18. Chuang L-H, Wang N-K, Yeung L, Chen Y-P, Hwang Y-S, Wu W-C, et al. Use of autologous whole blood during internal limiting membrane peeling and macular hole surgery is protective for indocyanine green toxicity. *Cutan Ocul Toxicol*. 2010 Jun 1;29(2):98–104.

19. Lai C-C, Hwang Y-S, Liu L, Chen K-J, Wu W-C, Chuang L-H, et al. Blood-Assisted Internal Limiting Membrane Peeling for Macular Hole Repair. *Ophthalmol*. 2009 Aug 1;116(8):1525–30.

20. Lai C-C, Wu W-C, Chuang L-H, Yeung L, Chen T-L, Lin K-K. Prevention of Indocyanine Green Toxicity on Retinal Pigment Epithelium with Whole Blood in Stain-Assisted Macular Hole Surgery. *Ophthalmol*. 2005 Aug 1;112(8):1409–14.

21. McCannel CA. Heparinized Autologous Blood for Visualization of the Internal Limiting Membrane During Macular Hole Surgery. *Retina* [Internet]. 2008;28(2). Available from: http://journals.lww.com/retinajournal/Fulltext/2008/02000/Heparinized_Autologous_Blood_for_Visualization_of.22.aspx

22. Than J, Balal S, Wawrzynski J, Nesaratnam N, Saleh GM, Moore J, et al. Fingerprick autologous blood: a novel treatment for dry eye syndrome. *Eye*. 2017 Jun 16;31:1655.

23. Lancaster GA, Dodd S, Williamson PR. Design and analysis of pilot studies: recommendations for good practice. *J Eval Clin Pract*. 2004 Jun 4;10(2):307–12.

24. Sim J, Lewis M. The size of a pilot study for a clinical trial should be calculated in relation to considerations of precision and efficiency. *J Clin Epidemiol.* 2012 Mar 1;65(3):301–8.

25. Julious SA. Sample size of 12 per group rule of thumb for a pilot study. *Pharm Stat.* 2005 Nov 24;4(4):287–91.

26. Ramsey SD, Willke RJ, Glick H, Reed SD, Augustovski F, Jonsson B, et al. Cost-Effectiveness Analysis Alongside Clinical Trials II—An ISPOR Good Research Practices Task Force Report. *Value Health*. 2015 Mar 1;18(2):161–72.

For peer review only - http://bmjopen.bmj.com/site/about/guidelines.xhtml

27. National Institute for Health and Clinical Excellence. NICE Guide to the methods of technology appraisal 2013 [Internet]. NICE; 2013. Available from: https://www.nice.org.uk/process/pmg9/chapter/introduction

28. Herdman M, Gudex C, Lloyd A, Janssen M, Kind P, Parkin D, et al. Development and preliminary testing of the new five-level version of EQ-5D (EQ-5D-5L). *Qual Life Res.* 2011 Dec 1;20(10):1727–36.

29. Fisher LD, Dixon DO, Herson J, Frankowski RK, Hearron MS, Peace KE. Intention to treat in clinical trials. In: Statistical issues in drug research and development. New York: Marcel Dekker; 1990. p. 331–50.

30. Schulz KF, Altman DG, Moher D. CONSORT 2010 Statement: updated guidelines for reporting parallel group randomised trials. *BMJ* [Internet]. 2010 Mar 24;340. Available from: http://www.bmj.com/content/340/bmj.c332.abstract

31. IBM. IBM SPSS Statistics for Windows, Version 22.0. Armounk, NY: IBM Corp.; 2012.

32. Cox D. Regression models and life-tables. *J R Stat Soc*. 1972;(34):187–220.

Authors' Contribution: SB and AS conceived the study with input from YP, EC, GB, MH, RB, SA, BS, MW and SP. AU, KH and MH developed the study protocol with statistical input from EC, qualitative design from YP and input on the economic evaluation phase from GB. AH reviewed the draft protocol for intellectual content with final review and approval by all the authors.

Funding Statement: This work was supported by NIHR Research for Patient Benefit grant number PB-PG-0416-20040.

Ethics Approval: Ethical approval for this study has been granted by the East of England - Cambridgeshire and Hertfordshire Research Ethics Committee (REC reference: 17/EE/0508).

Competing interest: None declared

For peer review only

For peer review only - http://bmjopen.bmj.com/site/about/guidelines.xhtml

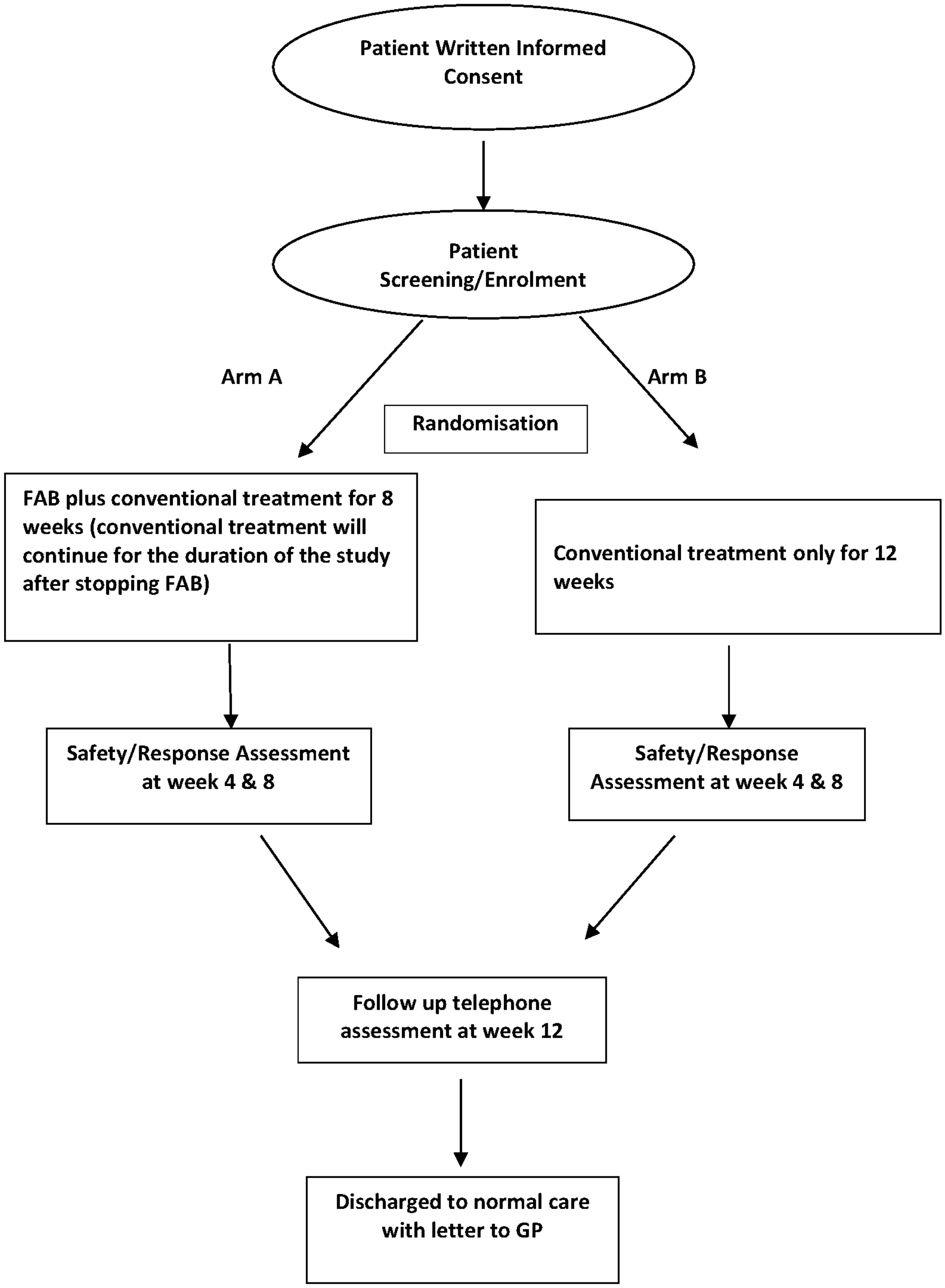
Patient Written Informed Consent
Patient Screening/Enrolment
Arm A
Arm B
Randomisation
FAB plus conventional treatment for 8 weeks (conventional treatment will continue for the duration of the study after stopping FAB)
Conventional treatment only for 12 weeks
Safety/Response Assessment at week 4 & 8
Safety/Response Assessment at week 4 & 8
Follow up telephone assessment at week 12
Discharged to normal care with letter to GP

Anglia Ruskin University | Postgraduate Medical Institute Clinical Trials Unit

The Feasibility of Fingerprick Autologous Blood (FAB) As a Novel Treatment for Severe Dry Eye Disease (DED): A Randomised Controlled Trial

Short title/Acronym: FAB in DED Trial

Sponsor: Bedford Hospital NHS Trust

Representative of the Sponsor: Dr Mohammad Wasil
Assistant Director, RDI
Bedford Hospital NHS Trust
Kempston Road
Bedford
MK42 9DJ

REC reference: 17/EE/0508

IRAS Project ID: 233813

For peer review only - http://bmjopen.bmj.com/site/about/guidelines.xhtml

Anglia Ruskin University | Postgraduate Medical Institute Clinical Trials Unit

Chief Investigator Agreement Page

The clinical study as detailed within this research protocol **(Version 1.4, dated 10 July 2018)** or any subsequent amendments will be conducted in accordance with the Research Governance Framework for Health & Social Care (2005), the World Medical Association Declaration of Helsinki (1996) and the current applicable regulatory requirements and any subsequent amendments of the appropriate regulations.

Chief Investigator Name: Mr Anant Sharma

Chief Investigator Site: Bedford Hospital NHS Trust

Signature and Date:

For peer review only - http://bmjopen.bmj.com/site/about/guidelines.xhtml

Postgraduate Medical Institute
Clinical Trials Unit

Statistician Agreement Page

The clinical study as detailed within this research protocol **(Version 1.4, dated 10 July 2018)** or any subsequent amendments will be conducted in accordance with the Research Governance Framework for Health & Social Care (2005), the World Medical Association Declaration of Helsinki (1996) and the current applicable regulatory requirements and any subsequent amendments of the appropriate regulations.

Statistician Name: Dr Erica Cook

Site: University of Bedfordshire, Bedford

Signature and Date:

For peer review only - http://bmjopen.bmj.com/site/about/guidelines.xhtml

Anglia Ruskin University | Postgraduate Medical Institute **Clinical Trials Unit**

Principal Investigator Agreement Page

The clinical study as detailed within this research protocol **(Version 1.4, dated 10 July 2018)** or any subsequent amendments will be conducted in accordance with the Research Governance Framework for Health & Social Care (2005), the World Medical Association Declaration of Helsinki (1996) and the current applicable regulatory requirements and any subsequent amendments of the appropriate regulations.

Principal Investigator Name:

Principal Investigator Site:

Signature and Date:

For peer review only

For peer review only - http://bmjopen.bmj.com/site/about/guidelines.xhtml

Anglia Ruskin University | Postgraduate Medical Institute **Clinical Trials Unit**

Study Summary/Synopsis

**TITLE**	The Feasibility of Fingerprick Autologous Blood (FAB) as a Novel Treatment for Severe Dry Eye Disease (DED): A Randomised Controlled Trial
**SHORT TITLE**	FAB in DED Trial
**Protocol Version Number and Date**	**Version 1.4, dated 10 July 2018**
**Methodology**	Single-blind randomised controlled Trial
**Study Duration**	24months
**Study Centre**	Bedford Hospital NHS Trust, Moorfields Eye Hospital London,
**Objectives**	To determine the feasibility of a definitive randomised controlled trial (RCT) to determine effectiveness of the use of fresh autologous blood (FAB) compared to conventional treatment for patients with dry eye syndrome.
**Number of Participants**	60
**Main Inclusion Criteria**	- Patient age > 18 years<br>- Severe symptomatic dry eye disease diagnosed by: Ocular Surface Disease Index (OSDI) score of greater than 33; OR Oxford Corneal Staining grade 2 or greater; OR Schirmer's without anaesthesia <5mm at 5 minutes.<br>- Patient able to consent<br>- Patients able and willing complete the quality of life (QoL) questionnaires required for the study
**Statistical Methodology and Analysis**	Data analysis will be primarily descriptive with means, standard deviations and frequency counts calculated for all variables of interest. Exploratory statistical analysis will compare the primary outcome and secondary outcomes between the intervention and control groups at 4 weeks, 8 weeks and 12 weeks (4 weeks post treatment)

For peer review only - http://bmjopen.bmj.com/site/about/guidelines.xhtml

Anglia Ruskin University | Postgraduate Medical Institute **Clinical Trials Unit**

Glossary of Terms and Abbreviations

AE	Adverse Event
AR	Adverse Reaction
ASR	Annual Safety Report
CA	Competent Authority
CI	Chief Investigator
CRF	Case Report Form
CRO	Contract Research Organisation
DMC	Data Monitoring Committee
EC	European Commission
GAfREC	Governance Arrangements for NHS Research Ethics Committees
ICF	Informed Consent Form
ISRCTN	International Standard Randomised Controlled Trial Number
MA	Marketing Authorisation
MS	Member State
Main REC	Main Research Ethics Committee
NHS R&D	National Health Service Research & Development
PI	Principle Investigator
QA	Quality Assurance
QC	Quality Control
Participant	An individual who takes part in a clinical trial
RCT	Randomised Controlled Trial
REC	Research Ethics Committee
SAE	Serious Adverse Event
SDV	Source Document Verification
SOP	Standard Operating Procedure
SSA	Site Specific Assessment
TMG	Trial Management Group
TSC	Trial Steering Committee

For peer review only

For peer review only - http://bmjopen.bmj.com/site/about/guidelines.xhtml

Anglia Ruskin University | Postgraduate Medical Institute **Clinical Trials Unit**

Table of Contents

1 Introduction ... 9
1.1 Background ... 9
1.2 Rationale and Risks/Benefits ... 11
2 Trial Objectives and Design ... 12
2.1 Primary Objective and Endpoints ... 12
2.2 Secondary Objective and Endpoints ... 12
2.3 Trial Design ... 13
2.3.1 Setting ... 14
2.4 Study Scheme Diagram ... 15
3 Participant Selection ... 16
3.1 Inclusion Criteria ... 16
3.2 Exclusion Criteria ... 16
4 Study Procedures ... 17
4.1 Informed Consent Procedures ... 17
4.2 Screening Procedures ... 17
4.3 Randomisation Procedures ... 18
4.4 Baseline Procedures ... 18
4.5 Schedule of Assessment ... 19
4.6 End of Study Definition ... 20
4.7 Participant Withdrawal ... 20
5 Pharmacovigilance ... 21
5.1 General Definitions ... 21
5.1.1 Adverse Event (AE) ... 21
5.1.2 Serious Adverse Event (SAE) ... 21
5.2 Investigators Assessment ... 21
5.2.1 Seriousness ... 21
5.2.2 Causality ... 21
5.2.3 Expectedness ... 21
5.2.4 Severity ... 22
5.3 Notification and reporting Adverse Events or Reactions ... 22
5.4 Notification and Reporting of Serious Adverse Events ... 22
5.5 Urgent Safety Measures ... 22
5.6 Annual Safety Reporting ... 23
5.7 Overview of the Safety Reporting Process/Pharmacoviligance responsibilities ... 23
6 Statistical Considerations ... 24
6.1 Qualitative approach ... 25
7 Data Handling & Record Keeping ... 27
7.1 Confidentiality ... 27
7.2 Study Documents ... 27
7.3 Case Report Form ... 28
7.4 Record Retention and Archiving ... 28
7.5 Compliance ... 28
7.6 Clinical Governance Issues ... 29
7.6.1 Ethical Considerations ... 29

For peer review only - http://bmjopen.bmj.com/site/about/guidelines.xhtml

Anglia Ruskin University | Postgraduate Medical Institute **Clinical Trials Unit**

7.7 Quality Control and Quality Assurance 29
7.7.1 Summary Monitoring Plan 29
7.7.2 Audit and Inspection 29
7.8 Non-Compliance 30
8 Trial Committees 31
9 Publication Policy 32
References 33
Appendix 36

For peer review only

For peer review only - http://bmjopen.bmj.com/site/about/guidelines.xhtml

Anglia Ruskin University | Postgraduate Medical Institute Clinical Trials Unit

1 Introduction

1.1 Background

Dry eye disease (DED) is an umbrella term encompassing a range of diseases estimated to affect 14% of all adults aged 48 to 91. If left untreated, DED can lead to severe reduction in the quality of life of the sufferer. It can also cause loss of vision, pain in response to light, painful recurring stabbing sensations, and the feeling of grit in the affected eye(s). Studies show that moderate to severe DED can be as disruptive as angina (1,2). Despite this, only less than 2% of all UK medical research funding is directed at diseases of the eye, leaving eye research critically underfunded (3).

No curative agents for DED exist. The treatment of the disorder is essentially symptomatic with standard non-surgical treatment focused on the use of artificial tears for lubrication and anti-inflammatory drugs. Anti-inflammatory agents include topical steroids and ciclosporin drops but these can cause side effects that limit their long term use (4). Topical steroids can also cause cataracts and glaucoma, all of which further limit its use. Surgical options for DED include punctal occlusion to reduce tear drainage, punctal cauterization (burning the drainage channel of tears, preventing their outflow) and partial suturing of the eyelids; but these are associated with adverse effects and varying levels of patient tolerability. Overall, available conventional treatment options for DED often only alleviate symptoms, have limited effectiveness, and in most cases patients may fail to respond; although the exact rate of treatment failure is unavailable in the published literature.

The mainstay of non-surgical treatment for DED focuses on the use of artificial tears. These can be purchased over the counter and include brand name products such as Viscotears® and Optrex® (5). Human tears contains an extensive range of growth factors, immunoglobulins, enzymes, cytokines, vitamins and electrolytes and these have been shown in numerous studies to be essential for the maintenance and proliferation of corneal epithelial cells as well as for defence against infection (6). It is well known that artificial tears fails to account for the extraordinarily complex composition of the natural tear film. Also, many artificial tears contain preservatives that have been shown to adversely affect the cornea (7). Crudely, human tears with its vast constituents is essentially filtered blood and as such is an obvious source for a

For peer review only - http://bmjopen.bmj.com/site/about/guidelines.xhtml

"tear mimic" containing the substances of tears. Blood, and several blood derived products, including autologous serum, have been studied as tear substitute candidates.

Autologous serum (AS) eye drops have been found in uncontrolled trials to be beneficial in DED patient by improving the ocular surface and reducing symptoms (8–10). However, a Cochrane review concluded that there is inconsistency in evidence on its benefit (11). Obtaining autologous serum requires frequent drawing of blood from the patient— a feature that excludes patients with anaemia or heart failure from using AS. Furthermore it also appears that 100% autologous serum is more beneficial than 50% serum and requires larger volumes of blood and/or more frequent venesection (9). Patients using AS also require access to a fridge as the product needs to be stored at low temperatures; a factor that is likely to be inconvenient for patients. In addition, AS is obtained by processing clotted blood which is often too expensive for the health service to consistently purchase, given the initial cost of £1653.56 and subsequent three-monthly cost of £1131.27 per patient.

The relatively high cost represents the biggest hurdle in the use of AS and is often the reason for delay or inaccessibility in starting treatment for DED using AS. However, we propose that finger prick autologous blood may be a simpler, cost-effective and possibly more acceptable method for treating dry eye disease. For this reason, this study proposes to test the use of finger prick autologous blood (FAB) technique in which whole blood is applied to the eye from a cleaned finger. Autologous fresh blood is already used subconjunctivally to help heal leaking trabeculectomy blebs (12–15). It is also used to help attach limbal autografts in cases of pterygium (16) and vitreoretinal macular hole surgery (17–20), with no adverse effects reported. The FAB method can be used immediately for patients who are awaiting conventional treatment for autologous blood. The objective of this study is therefore to investigate if serum via a drop of fresh blood is an effective treatment for severe dry eye disease which currently require venesected autologous blood and whether this would be particularly useful in the group of patients in whom venesection is contraindicated.

For peer review only - http://bmjopen.bmj.com/site/about/guidelines.xhtml

Anglia Ruskin University | Postgraduate Medical Institute Clinical Trials Unit

1.2 Rationale and Risks/Benefits

This study proposes to test the use of whole, fresh, autologous blood as a treatment for severe dry eye disease. The blood can be obtained from a cleaned finger, pricked by the patient using a diabetic lancet and administered immediately to the dry eye. This allows the delivery of not only most of the aforementioned beneficial components of tears but additional growth factors and proteins, fresh and unprocessed, which can help heal the ocular surface. If validated, this may replace current autologous serum practice and its ease of use, vastly reduced cost with greater convenience may mean that it could be extended to other ocular surface diseases.

The proposing team have completed an exploratory study on the use of finger-prick autologous blood (FAB) for persistent epithelial defects and severe dry eye disease and preliminary results indicate improvement with no adverse events reported (21). The exploratory study included 16 patients with a diagnosis of severe to moderate dry eye syndrome and used the FAB method for treatment. The findings of the study demonstrated mean improvements in visual acuity, Oxford corneal staining grade, tear breakup time, Schirmer's test and dry eye disease questionnaire score. The response rate from participants was good with only a single patient who met the inclusion criteria not wishing to participate in the trial. Both the amount of staining (indicating inflammation and ocular surface damage) and their DED questionnaire scores (indicating severity of their symptoms and impact on quality of life) showed mean improvement which reached statistical significance. A search of clinical databases (Medline, CINAHL and AMED) including on-going trials on the UK CRN portfolio database and related websites (www.controlled-trials.com) did not identified any studies using FAB as a treatment for dry eye disease.

For peer review only - http://bmjopen.bmj.com/site/about/guidelines.xhtml

Anglia Ruskin University | Postgraduate Medical Institute **Clinical Trials Unit**

2 Trial Objectives and Design

2.1 Primary Objective and Endpoints

The primary objective is to determine the feasibility of a definitive randomised controlled trial (RCT) to evaluate effectiveness of the use of fresh autologous blood (FAB) compared to conventional treatment for patients with dry eye syndrome. This will involve specifically assessing the following endpoints:

i. Number of eligible patients within the study population and recruitment time frame.
ii. Percentage of eligible patients accepted for randomisation.
iii. Patient compliance with trial protocol, measured as per self-reported adherence to intervention.
iv. Percentage of patients completing study.

2.2 Secondary Objective and Endpoints

The secondary objectives are:

- To determine the effectiveness of the trial intervention and further explore the acceptability of the study design.
- To explore the feasibility of collecting resource use and quality of life data, to inform the design of the health economics component of a future definitive trial.

The secondary end points, which have not been powered for within the feasibility study, include:

For peer review only - http://bmjopen.bmj.com/site/about/guidelines.xhtml

Anglia Ruskin University | Postgraduate Medical Institute **Clinical Trials Unit**

Outcome Measure	Endpoint
Reduction in corneal inflammation as indicated by staining on front of the eye	Oxford Corneal Staining Guide
Patient pain and symptoms scores	OSDI score
Improvement in objective signs of dry eye disease as indicated by visual acuity	Schirmer's test, tear breakup time, lower and/or upper tear menisci height measurement
Willingness for patients to be randomised and acceptability of the intervention	Structured qualitative interviews
Impact on patients' quality of life	EQ-5D-5L score
Cost to the NHS and patient	Use of additional NHS services and privately purchased over the counter treatments related to dry eyes disease
Safety measure of pressure within eye	Intra ocular pressure (IOP) score

2.3 Trial Design

Single-blind two-arm feasibility randomised controlled trial of FAB with conventional treatment versus conventional therapy alone for dry eye disease, including a qualitative process evaluation.

- ***Arm A – FAB plus conventional treatment***

The patients will use FAB therapy alongside conventional therapy (artificial tears, ciclosporin drops and punctal plugs/cautery) as recommended by their treating ophthalmologist. A fingertip of the hand will be wiped with an alcohol steret and self-pricked using a standard diabetic lancet. The drop of blood is produced as normal and applied to the lower fornix of the affected eye(s) with the lower lid pulled down slightly by the patient. The blood will be applied four times a day. A fresh finger should be used for each eye. The blood will be wiped away with the alcohol steret. Nails should be kept short and nail varnish should be avoided. FAB should be applied at least 15 minutes after any artificial tears and no drops for at least half an hour afterwards.

For peer review only - http://bmjopen.bmj.com/site/about/guidelines.xhtml

- ***Arm B – Conventional treatment only***

The patients will use conventional therapy (artificial tears, cyclosporin drops and punctal plugs/cautery) as recommended by their treating ophthalmologist.

2.3.1 **Setting**

The study will take place at NHS sites. The main site will be Bedford Hospital NHS Trust and Moorfields Eye Hospital London. Patients will be recruited from corneal and external eye disease clinics at these sites.

For peer review only

For peer review only - http://bmjopen.bmj.com/site/about/guidelines.xhtml

Anglia Ruskin University | Postgraduate Medical Institute **Clinical Trials Unit**

2.4 Study Scheme Diagram

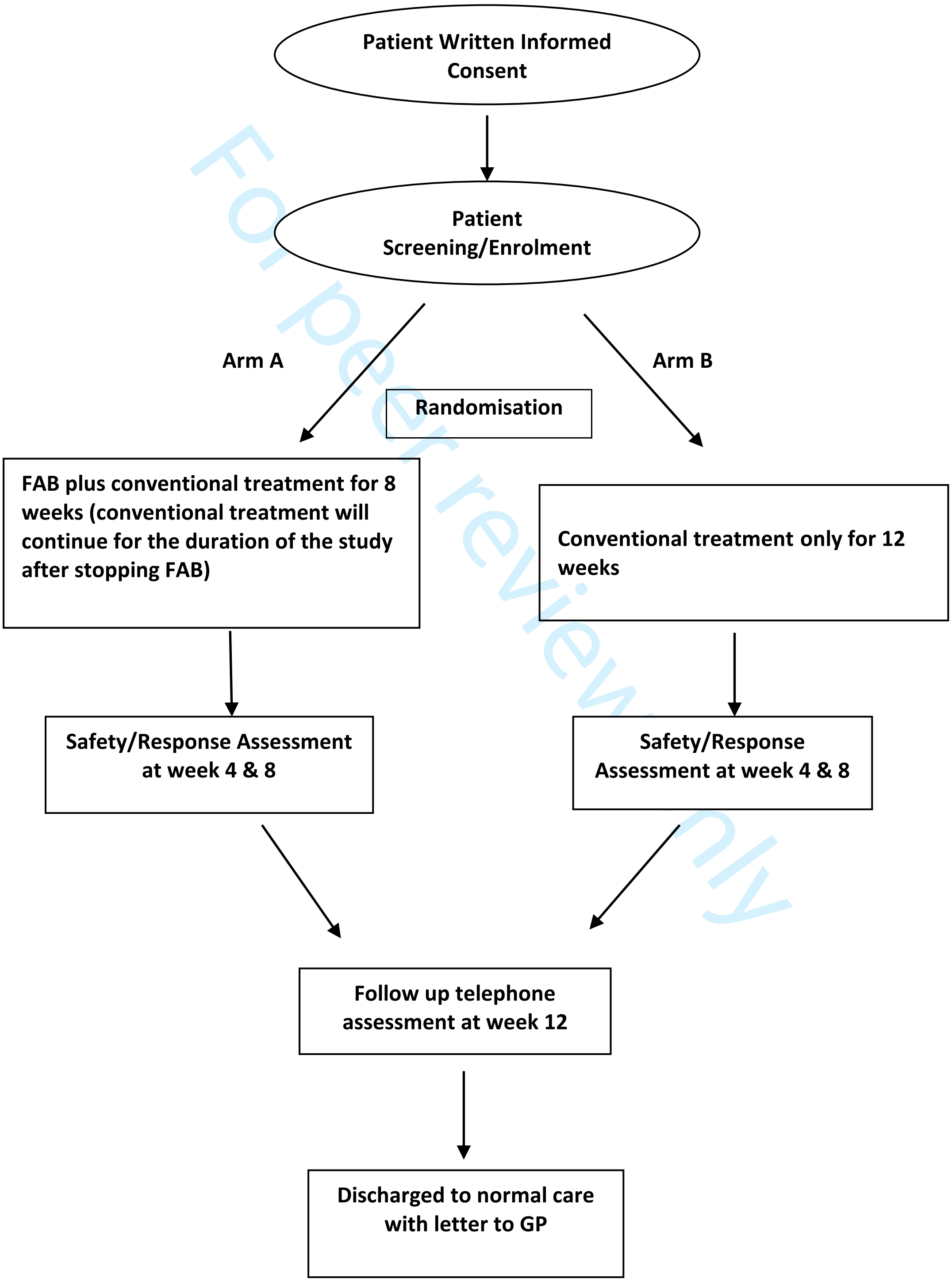

For peer review only - http://bmjopen.bmj.com/site/about/guidelines.xhtml

Anglia Ruskin University | Postgraduate Medical Institute **Clinical Trials Unit**

3 Participant Selection

60 patients (30 in each arm) will be recruited into the study. This allows for an attrition rate of 10%.

3.1 Inclusion Criteria

i. Patient age ≥ 18 years
ii. Severe symptomatic dry eye disease diagnosed by: Ocular surface disease index (OSDI) score of greater than 33; OR Oxford Corneal Staining grade 2 or greater; OR Schirmer's without anaesthesia <5mm at 5 minutes
iii. Patients on artificial tears and/or lubricating drops/gel four times a day
iv. Patient able to give consent
v. Patients able and willing complete the quality of life (QoL) questionnaires required for the study

3.2 Exclusion Criteria

i. Fear of needles
ii. Unable or not willing to carry out repeat finger pricks
iii. Patients with infected finger/s or systemic infection or on systemic antibiotics for infection.
iv. Patients with active ocular infection, active immunological corneal melt, or recurrent corneal erosion.
v. Pregnant or breast feeding women
vi. Previous use of FAB treatment (e.g. from exploratory study)
vii. Systemic illness causing immune system deficiency
viii. Graft versus host disease
ix. Previous use of autologous serum within 3 months
x. Diabetes

For peer review only - http://bmjopen.bmj.com/site/about/guidelines.xhtml

4 Study Procedures

4.1 Informed Consent Procedures

It is the responsibility of the Investigator, or appropriately GCP trained person delegated by the Investigator as documented in the site delegation log, to obtain written informed consent from each participant prior to any participation/study specific procedures. This should follow adequate explanation of the aims, methods, anticipated benefits and potential hazards of the study. The participants will be given ample time to consider giving their consent for the study. For this study a minimum of 24 hours will be given during which the consenting team will be contactable over the phone to answer any questions. The date that the Participant Information Sheet (PIS) is given to the participant must be documented within the patient's notes to confirm that sufficient time was given. The Investigator (or other qualified person) will explain to the potential participant that they are free to refuse any involvement within the study or alternatively withdraw their consent at any point during the study and for any reason.

If there is any further safety information which may result in significant changes in the risk/benefit analysis, the PIS and Informed Consent Form (ICF) will be reviewed and updated accordingly. All participants that are actively enrolled on the study will be informed of the updated information and given a revised copy of the PIS/ICF in order to confirm their wish to continue on the study.

4.2 Screening Procedures

Patients will have undergone the below procedures as per standard of care which will in turn feed in to the investigator for their review prior to approaching the potential participant.

- Ocular surface disease index (OSDI) score of greater than 33; OR
- Oxford Corneal Staining grade 2 or greater; OR
- Schirmer's without anaesthesia <5mm at 5 minutes

The results of these which will be reviewed by investigator prior to approaching the potential participant.

For peer review only - http://bmjopen.bmj.com/site/about/guidelines.xhtml

Anglia Ruskin University | Postgraduate Medical Institute **Clinical Trials Unit**

4.3 Randomisation Procedures

Randomisation will be carried out by the Anglia Ruskin Clinical Trials Unit (ARCTU) using the SEALED ENVELOPE randomisation service. SEALED ENVELOPE is a randomisation and online database service developed for clinical trial services. It is an internet based system and will be set for this study by ARCTU in accordance with the protocol. Enrolled patients will undergo 1:1 block randomisation to Arm A or B. The system stores the pre-determined sequence of randomisation and this list is visible to neither the investigator nor ARCTU staff. Once a patient has consented to take part in the trial, they will be randomly allocated to either Arm A to receive FAB and convention therapy or Arm B to receive conventional therapy only. The Research Nurse or Fellow or designated staff will log on to a web browser application and enter the patient's eligibility factors into the system. The treatment allocation will be sent to the research team who will make the necessary arrangements for the patient's treatment plan.

The treating ophthalmologist in clinic will prescribe and counsel all patients on the correct technique for conventional treatment. The unblinded research nurses will provide additional training to the patients on the method of delivering FAB, including the use of a training video. Patients will be advised by the research team to inform their ophthalmologist if they have been started on any new treatment during the trial period by other clinicians or themselves over the counter.

4.4 Baseline Procedures

- Patients will undergo the following at baseline:
- Physical examination
- BP and pulse
- Visual acuity test
- Lower and/or upper tear menisci test on optical coherence tomography (OCT, where available)
- Tear breakup time (TBUT)
- Intraocular pressure (IOP)
- Schirmer's test
- Oxford corneal staining grade
- Patient pain and symptom score (OSDI score)
- Lissamine green stain assessment (if available)
- Anterior segment colour photography and cobalt blue fluorescein eye stain photography (if available)
- Anterior segment optical coherence topography (OCT) (if available)
- MMP-9 tear swap (if available)

For peer review only - http://bmjopen.bmj.com/site/about/guidelines.xhtml

For peer review only

4.5 Schedule of Assessment

Procedures	Screening (as per standard of care)	Baseline Visit (Day 1)	Week 4 visit (+/- 7days	Week 8 visit (+/- 7days)	Week 12 follow up (Telephone, +/- 7days)
Informed consent		x			
Demographics		x			
Medical history		x			
Physical examination		x	x	x	
BP and pulse		x	x	x	
Current medical conditions		x	x	x	
Concomitant medications		x	x	x	
Eligibility assessment	x				
Randomisation		x			
Visit dates and appointment		x	x	x	
FAB treatment (Group A only)		x	x	x	
**Treatment and Follow up Phase**					
Completion of QoL questionnaire (EQ-5D-5L)		x	x	x	x
Completion of Health resource questionnaire			x	x	x
Review treatment diary			x	x	
Schirmer's test without anaesthesia (if other criteria not met)	x	x	x	x	
Patient pain and symptom score (OSDI score) if other criteria not met	x	x	x	x	x
Visual acuity		x	x	x	
Lissamine green stain assessment (if available)		x	x	x	
Anterior segment colour photography and cobalt blue fluorescein eye stain photography (if available)		x	x	x	
Anterior segment optical coherence topography (OCT) (if available)		x	x	x	
MMp-9 tear swap (if available)		x		x	
Oxford corneal staining grade	x	x	x	x	
Lower and/or upper tear menisci height (if available)		x	x	x	
Tear breakup time		x	x	x	
Qualitative interview				x	

For peer review only - http://bmjopen.bmj.com/site/about/guidelines.xhtml

Anglia Ruskin University | Postgraduate Medical Institute **Clinical Trials Unit**

4.6 End of Study Definition

The definition of the end of study will be the point where the last patient recruited had the last follow-up visit.

4.7 Participant Withdrawal

Patients will be withdrawn from study based on the following:

- New diagnosis of infection on finger or eye
- Requesting to be withdrawn for other reasons such as inconvenience of increased clinic visits
- Pregnancy after recruitment
- Systemic infection, or systemic antimicrobials for infection.
- If finger sore from repeated prick and patient does not want to use another finger.

Any adverse effects will be reported back to the lead investigator at the site and if necessary patients

For peer review only

For peer review only - http://bmjopen.bmj.com/site/about/guidelines.xhtml

Anglia Ruskin University | Postgraduate Medical Institute **Clinical Trials Unit**

5 Pharmacovigilance

5.1 General Definitions

5.1.1 Adverse Event (AE)

An AE is any untoward medical occurrence in a participant to whom a medicinal product has been administered, including occurrences which are not necessarily caused by or related to that product. An AE can therefore be any unfavourable and unintended sign (including an abnormal laboratory finding), symptom or disease temporarily associated with study activities.

5.1.2 Serious Adverse Event (SAE)

An SAE fulfils at least one of the following criteria:

- Is fatal – results in death (NOTE: death is an outcome, not an event)
- Is life-threatening
- Requires inpatient hospitalisation or prolongation of existing hospitalisation
- Results in persistent or significant disability/incapacity
- Is a congenital anomaly/birth defect
- Is otherwise considered medically significant by the Investigator

5.2 Investigators Assessment

5.2.1 Seriousness

The Chief/Principal Investigator responsible for the care of the participant, or in his absence an authorised medic within the research team, is responsible for assessing whether the event is serious according to the definitions given in section 5.1.

5.2.2 Causality

The Investigator must assess the causality of all serious adverse events in relation to the trial treatment according to the definition given

5.2.3 Expectedness

The investigator must assess the expectedness of all SAEs according to the definition given. If the SAE is unexpected and related, then it needs immediate reporting.

For peer review only - http://bmjopen.bmj.com/site/about/guidelines.xhtml

Anglia Ruskin University | Postgraduate Medical Institute Clinical Trials Unit

5.2.4 Severity

The Investigator must assess the severity of the event according to the following terms and assessments. The intensity of an event should not be confused with the term "serious" which is a regulatory definition based on participant/event outcome criteria.

Mild: Some discomfort noted but without disruption of daily life
Moderate: Discomfort enough to affect/reduce normal activity
Severe: Complete inability to perform daily activities and lead a normal life

5.3 Notification and reporting Adverse Events or Reactions

If the AE is not defined as SERIOUS, the AE is recorded in the study file and the participant is followed up by the research team. The AE is documented in the participants' medical notes (where appropriate) and the CRF.

5.4 Notification and Reporting of Serious Adverse Events

Serious Adverse Event (SAEs) that are considered to be 'related' and 'unexpected' are to be reported to the sponsor within 24 hours of learning of the event and to the Main REC within 15 days in line with the required timeframe. For further guidance on this matter, please refer to Appendix.

5.5 Urgent Safety Measures

The CI may take urgent safety measures to ensure the safety and protection of the clinical trial participants from any immediate hazard to their health and safety, in accordance with Regulation 30. The measures should be taken immediately. In this instance, the approval of the Licensing Authority Approval prior to implementing these safety measures is not required. However, it is the responsibility of the CI to inform the sponsor and Main Research Ethics Committee (via telephone) of this event **immediately**.

The CI has an obligation to inform both the Main Ethics Committee **in writing within 3 days**, in the form of a substantial amendment. The sponsor must be sent a copy of the

For peer review only - http://bmjopen.bmj.com/site/about/guidelines.xhtml

correspondence with regards to this matter. For further guidance on this matter, please refer to Appendix.

5.6 Annual Safety Reporting

The CI will send the Annual Progress Report to the main REC using the NRES template (the anniversary date is the date on the MREC "favourable opinion" letter from the MREC) and to the sponsor. Please see appendix for further information.

5.7 Overview of the Safety Reporting Process/Pharmacoviligance responsibilities

The CI/PI has the overall pharmacovigilance oversight responsibility. The CI/PI has a duty to ensure that pharmacovigilance monitoring and reporting is conducted in accordance with the sponsor's requirements.

For peer review only - http://bmjopen.bmj.com/site/about/guidelines.xhtml

Anglia Ruskin University | Postgraduate Medical Institute **Clinical Trials Unit**

6 Statistical Considerations

A detailed data validation will examine completeness, existence and accuracy of collected data to assess data quality and identify missing and conflicting data. To distinguish missing value mechanisms visualisation methods will be used [R package; Visualization and Imputation of Missing (VIM)] (22). Assumptions based on data Missing At Random (MAR)— that is, that a value is missing depends only on observed values and not on unobserved values (23)— will be imputed using multivariate imputations procedures [(R package; Multivariate Imputation by Chained Equations (MICE) (24)]. The statistical analysis will be performed on an intention-to-treat basis and per-protocol and will be reported according to 2010 CONSORT guidelines (25). All statistical analyses will be completed using SPSS Statistics V.22.0 (26). A p-value of <.05 will be accepted as statistical significance.

Conforming to recommendations for feasibility studies, data analysis will be primarily descriptive, with means, standard deviations and frequency counts calculated for all variables of interest. Exploratory efficacy analysis will compare the primary outcome variables derived from the data collected at 8 weeks between the two arms using a marginal general linear model (GLM) with robust standard errors, to allow for clustering by group. Secondary outcomes between the intervention and control groups will be compared at 4 weeks, 8 weeks and 12 weeks (4 weeks post treatment). Safety outcome measures will include IOP rise and any reported infection.

Secondary analyses of the primary outcome, controlling for baseline covariates, will be performed using proportional hazards regression models (27). Analysis of treatment discontinuation adjusted for clinical site, baseline status, and whether the patient is randomized to the FAB or control (conventional treatment only) group will be conducted. Any interaction between baseline status or regimen and treatment will also be assessed. Interactions between significant covariates and treatment effects will be assessed in the context of the proportional hazards models.

For peer review only - http://bmjopen.bmj.com/site/about/guidelines.xhtml

Anglia Ruskin University | Postgraduate Medical Institute **Clinical Trials Unit**

6.1 Qualitative approach

A nested qualitative approach using in-depth, semi-structured interviews will help us understand the lived experience of people using FAB and factors relating to the ways that clinical departments and healthcare professionals adapt to working with FAB. Topics, questions and probes in the interviews will emerge from three sources: a) the relevant literature; b) our experience of and involvement in clinical ophthalmology; c) consultation with patients. In this feasibility study, we will use a convenience sampling technique for the qualitative work. We will interview all clinicians who administer the intervention in the study and we will aim to interview 10 patients (adjusted for setting, gender and age group representation). Given the resources, we will aim to draw findings from a larger sample in a future study. Interviews will be recorded, transcribed and analysed using the framework analysis. We will structure the analysis of collected data to fit two evaluation frameworks relevant to the successful implementation of FAB:

1) Patient-oriented: Understand the lived experience of patients using FAB with emphasis on intervention acceptability, perceived enablers and barriers for adherence, perceived clarity of advice and guidance and factors relating to the initial uptake of the intervention.
2) Organisation-oriented: Whether and how the use of FAB affected workload and/or workflow; identify process change, adaptation challenges, skills gap; establish treatment fidelity and clinician acceptability.

This phase will work in parallel to the quantitative data collection so that emerging themes can be investigated in later interviews. Even though the feasibility study sample is reasonably limited, we anticipate some useful insights about the use of FAB to occur because of the triangulation between the RCT and the qualitative findings (e.g. reasons for partial adherence).

6.2 Economic Evaluation

Good practice recommendations for cost-effectiveness analyses (28) suggest concentrating on the measurement of large cost drivers, with less focus on resources that are not expected to differ between different treatments. Estimation of cost-effectiveness is therefore an iterative

For peer review only - http://bmjopen.bmj.com/site/about/guidelines.xhtml

Anglia Ruskin University | Postgraduate Medical Institute **Clinical Trials Unit**

process and by including a health economic component in a feasibility it is possible to consider how the methods might be refined in any future definitive study. In order to estimate costs, informed by previous data collection instruments (Database of Instruments for Resource Use Measurement: http://www.dirum.org/) and NICE guidance (29), a self-report questionnaire will be devised. This will capture the use of NHS services and privately purchased over the counter treatments such as OPTREX. For benefits, the EQ-5D-5L (30) will be used to measure quality of life, enabling QALY (Quality Adjusted Life Year) scores to be calculated. The main purpose of the economic analysis is to inform the decision regarding how and what cost and effect data would be collected within a more definitive study. in order to inform this decision, we will estimate completion rates and seek to identify big cost drivers.

For peer review only

For peer review only - http://bmjopen.bmj.com/site/about/guidelines.xhtml

Anglia Ruskin University | Postgraduate Medical Institute **Clinical Trials Unit**

7 Data Handling & Record Keeping

7.1 Confidentiality

The Investigator has a responsibility to ensure that participant anonymity is protected and maintained. They must also ensure that their identities are protected from any unauthorised parties. Information with regards to study participants will be kept confidential and managed in accordance with the Data Protection Act, NHS Caldicott Guardian, The Research Governance Framework for Health and Social Care and Research Ethics Committee Approval.

7.2 Study Documents

- A signed protocol and any subsequent amendments
- Investigator's Brochure
- Sponsor Self-Monitoring template for the trial team to complete on a regular basis as detailed by the Monitoring section
- Current/Superseded Participant Information Sheets (as applicable)
- Current/Superseded Consent Forms (as applicable)
- Indemnity documentation from sponsor
- Conditions of Sponsorship from sponsor
- Conditional/Final R&D Approval
- Signed site agreement
- Ethics submissions/approvals/correspondence
- CVs of CI and site staff
- Delegation log
- Staff training log
- Site signature log
- Participant identification log
- Screening log
- Enrolment log
- Monitoring visit log
- Protocol training log
- Correspondence relating to the trial
- Communication Plan between the CI/PI and members of the study team
- SAE reporting plan for the study

For peer review only - http://bmjopen.bmj.com/site/about/guidelines.xhtml

Anglia Ruskin University | Postgraduate Medical Institute **Clinical Trials Unit**

7.3 Case Report Form

Project data collection will be managed by the Clinical Trials Unit Data Manager. The responsibility for data entry rests with the Research Nurse or designated staff who is supported by the Investigator. ARCTU uses an online data management system called MACRO to design and manage eCRFs (Electronic Case Report Forms). ARCTU will work together with the sponsor to design and validate the data collection tools so that they are appropriate for this study. Once a patient is enrolled on the study the research team can access these forms remotely through the Internet portal and study data will be entered and captured for the study. All data will be in anonymised form and patients will be identifiable only by study number. Data will be remotely monitored by ARCTU and discussed at data monitoring committee meetings. Any inconsistencies, validation errors or inaccuracies will be reported to the lead investigator regularly. Once data collection is complete and the data has been validated, a data lock will be performed and analysis will begin.

7.4 Record Retention and Archiving

During the course of research, all records are the responsibility of the Chief Investigator and must be kept in secure conditions. When the research trial is complete, it is a requirement of the Research Governance Framework and Trust Policy that the records are kept for a further 20 years.

7.5 Compliance

The CI will ensure that the trial is conducted in compliance with the principles of the Declaration of Helsinki (1996), and in accordance with all applicable regulatory requirements including but not limited to the Research Governance Framework, Trust and Research Office policies and procedures and any subsequent amendments.

For peer review only - http://bmjopen.bmj.com/site/about/guidelines.xhtml

Anglia Ruskin University | Postgraduate Medical Institute Clinical Trials Unit

7.6 Clinical Governance Issues

7.6.1 Ethical Considerations

This protocol and any subsequent amendments, along with any accompanying material provided to the participant in addition to any advertising material will be submitted by the Investigator to an Independent Research Ethics Committee. Written Approval from the Committee will be obtained and subsequently submitted to the trusts Research and Development office to obtain Final R&D approval.

7.7 Quality Control and Quality Assurance

7.7.1 Summary Monitoring Plan

ARCTU will ensure that the project is carried out in accordance with the Research Governance Framework. All research team members will have GCP (Good Clinical Practice) training before the research commences to ensure every aspect from trial design to dissemination is carried out in line with these principals. GCP is an international quality standard that is provided by International Conference on Harmonisation (ICH), an international body that defines standards, which governments can transpose into regulations for clinical trials involving human subjects.

7.7.2 Audit and Inspection

Auditing: Definition "A systematic and independent examination of trial related activities and documents to determine whether the evaluated trial related activities were conducted, and the data were recorded, analysed and accurately reported according to the protocol, sponsor's standard operating procedures (SOPs), Good Clinical Practice (GCP), and the applicable regulatory requirement(s)."

The FAB in DED study may receive an audit by any of the methods listed below:

For peer review only - http://bmjopen.bmj.com/site/about/guidelines.xhtml

Anglia Ruskin University | Postgraduate Medical Institute **Clinical Trials Unit**

- A project may be identified via the risk assessment process.
- An individual investigator or department may request an audit.
- A project may be identified via an allegation of research misconduct or fraud or a suspected breach of regulations.
- Projects may be selected at random. The Department of Health states that Trusts should be auditing a minimum of 10% of all research projects.
- Projects may be randomly selected for audit by an external organisation.

Internal audits will be conducted by a sponsor's representative

7.8 Non-Compliance

Non-compliance can be defined as 'a noted systematic lack of both the CI and the study staff adhering to SOPs/protocol/ICH-GCP, which leads to prolonged collection of deviations, breaches or suspected fraud.'

These non-compliances may be captured from a variety of different sources including monitoring visits, CRFs, communications and updates. The sponsor will maintain a log of the non-compliances to ascertain if there are any trends developing which to be escalated. The sponsor will assess the non-compliances and action a timeframe in which they need to be dealt with. Each action will be given a different timeframe dependant on the severity. If the actions are not dealt with accordingly, the sponsor will agree an appropriate action, including an on-site audit.

For peer review only - http://bmjopen.bmj.com/site/about/guidelines.xhtml

Anglia Ruskin University | Postgraduate Medical Institute Clinical Trials Unit

8 Trial Committees

Trial Management Group (TMG)

A Trial Management Group (TMG) has been formed comprising the Chief Investigator, other lead investigators (clinical and non-clinical) and members of the Data Centres. The TMG will be responsible for the day-to-day running and management of the trial and will meet at least 3 times a year by teleconference.

Trial Steering Committee (TSC)

The Trial Steering Committee (TSC) has membership from TMG plus independent members, including the chair. The role of the TSC is to provide overall supervision for the trial and provide advice through its independent Chairman. The ultimate decision for the continuation of the trial lies with the TSC.

Independent Data Monitoring Committee (IDMC)

The Independent Data Monitoring Committee (IDMC) is the only group who sees the confidential, accumulating data to the trial. Reports to the IDMC will be produced by the trial statistician. The IDMC will meet within 6 months of the trial opening with the frequency of meetings dictated by the IDMC. The IDMC will consider the report for the interim analysis based on the first 30 patients and will decide, keeping in mind the rules described in the statistics section, whether to recommend that the study should continue or be closed. The primary outcome measure for the study addresses toxicity and so it aligns with the IDMC's precautionary role in patient safety. The details of the interim analysis will remain confidential to the IDMC until the study is closed.

For peer review only - http://bmjopen.bmj.com/site/about/guidelines.xhtml

Anglia Ruskin University | Postgraduate Medical Institute Clinical Trials Unit

9 Publication Policy

Data obtained from this study will be published in a suitable peer-review journal and also presented at international ophthalmic conferences including American Academy of Ophthalmology, Royal College of Ophthalmology annual congress, Association for Research and Vision and Ophthalmology, and the European Society of Cataract and Refractive Surgery. Information would be provided to patient groups and charities such as the Sjogren's society and the Royal National Institute of Blind People.

For peer review only

For peer review only - http://bmjopen.bmj.com/site/about/guidelines.xhtml

References

1. Hom MM, Nguyen A, Bielory L. Allergic conjuctivitis and dry eye syndrome. Ann Allergy Asthma Immunol Off Publ Am Coll Allergy Asthma Immunol. 2012;108:163–6.

2. Schiffman RM, Walt JG, Jacobsen G, Doyle JJ, Lebovics G, Sumner W. Utility assessment among patients with dry eye disease. Ophthalmology. 2003;110(7):1412–9.

3. National Eye Research Centre. Eye research overview [Internet]. 2016. Available from: https://www.nerc-charity.org.uk/our-eye-research

4. Kymionis GD, Bouzoukis DI, Diakonis VF, Siganos C. Treatment of chronic dry eye: focus on cyclosporine. Clin Ophthalmol Auckl NZ. 2008 Dec;2(4):829–36.

5. Diebold Y, Herreras JM, Callejo S, Argueso P, Calonge M. Carbomer- Versus Cellulose-Based Artificial-Tear Formulations: Morphologic and Toxicologic Effects on a Corneal Cell Line. Cornea. 1998;17(4):433.

6. Yokoi N, Yamada H, Mizukusa Y, Bron AJ, Tiffany JM, Kato T, et al. Rheology of Tear Film Lipid Layer Spread in Normal and Aqueous Tear–Deficient Dry Eyes. Invest Ophthalmol Vis Sci. 2008 Dec 1;49(12):5319–24.

7. Pfister R, Burstein N. The effects of opthalmic drugs, vehicles and preservatives on corneal epithelium: a scanning electron microscope study. Invest Ophthalmol Vis Sci. 1976;15(4):246–59.

8. Fox R, Chan R, Michelson J, Belmont J, Michelson P. Beneficial effect of artificial tears made with artificial tears made with autologous serum in patients with keratoconjunctivitis sicca. Arthritis Rheumatol. 1984;29:577–83.

9. Poon AC, Geerling G, Dart JKG, Fraenkel GE, Daniels JT. Autologous serum eyedrops for dry eyes and epithelial defects: clinical and in vitro toxicity studies. Br J Ophthalmol. 2001 Oct 1;85(10):1188.

10. Tsubota K, Goto E, Fujuta H, Ono M, Inuoue H, Saito I. Treatment of dry eye by autologous serum application in Sjogren's Syndrome. Br J Ophthalmol. 1999;83:390–5.

11. Pan Q, Angelina A, Zambrano A, Marrone M, Stark WJ, Heflin T, et al. Autologous serum eye drops for dry eye. Cochrane Database Syst Rev [Internet]. 2013;(8). Available from: http://dx.doi.org/10.1002/14651858.CD009327.pub2

12. Biswas S, Zaheer I, Monsalve B, Diamond JP. Compression sutures with autologous blood injection for leaking trabeculectomy blebs. Br J Ophthalmol. 2009 Mar 25;93(4):549.

13. Burnstein A, WuDunn D, Ishii Y, Jonescu-Cuypers C, Cantor LB. Autologous blood injection for late-onset filtering bleb leak. Am J Ophthalmol. 2001 Jul 1;132(1):36–40.

For peer review only - http://bmjopen.bmj.com/site/about/guidelines.xhtml

Anglia Ruskin University | Postgraduate Medical Institute **Clinical Trials Unit**

14. Dinah C, Bhachech B, Ghosh G. Long-term success with autologous blood injection for leaking trabeculectomy blebs. Br J Ophthalmol. 2010 Mar 9;94(3):392.

15. Haynes WL, Alward WLM. Combination of Autologous Blood Injection and Bleb Compression Sutures to Treat Hypotony Maculopathy. J Glaucoma [Internet]. 1999;8(6). Available from: http://journals.lww.com/glaucomajournal/Fulltext/1999/12000/Combination_of_Autologous_Blood_Injection_and_Bleb.8.aspx

16. de Wit D, Athanasiadis I, Sharma A, Moore J. Sutureless and glue-free conjunctival autograft in pterygium surgery: a case series. Eye. 2010 Sep;24(9):1474–7.

17. Chuang L-H, Wang N-K, Yeung L, Chen Y-P, Hwang Y-S, Wu W-C, et al. Use of autologous whole blood during internal limiting membrane peeling and macular hole surgery is protective for indocyanine green toxicity. Cutan Ocul Toxicol. 2010 Jun 1;29(2):98–104.

18. Lai C-C, Hwang Y-S, Liu L, Chen K-J, Wu W-C, Chuang L-H, et al. Blood-Assisted Internal Limiting Membrane Peeling for Macular Hole Repair. Ophthalmology. 2009 Aug 1;116(8):1525–30.

19. Lai C-C, Wu W-C, Chuang L-H, Yeung L, Chen T-L, Lin K-K. Prevention of Indocyanine Green Toxicity on Retinal Pigment Epithelium with Whole Blood in Stain-Assisted Macular Hole Surgery. Ophthalmology. 2005 Aug 1;112(8):1409–14.

20. McCannel CA. Heparinized Autologous Blood for Visualization of the Internal Limiting Membrane During Macular Hole Surgery. Retina [Internet]. 2008;28(2). Available from: http://journals.lww.com/retinajournal/Fulltext/2008/02000/Heparinized_Autologous_Blood_for_Visualization_of.22.aspx

21. Balal S, Sharma A, Nesaratnam N, Than J, Wawrzynski J, Patel A. Fingerprick autologous blood: a novel treatment for dry eye syndrome and persistent epithelial defects In: Cornea Medical II [Internet]. Copehagen: European Society of Cataract & Refractive Surgeons; 2016. Available from: http://www.escrs.org/abstracts/details.asp?confid=22&sessid=819&type=Free&paperid=27426

22. VIM. Visualization and Imputation of Missing Values [Internet]. 2017. Available from: [http://cran.r-project.org/package=VIM]

23. Schafer JL, John W. Graham. Missing Data: Our View of the State of the Art. Psychol Methods. 2002;7(2):147–77.

24. Azur MJ, Stuart EA, Frangakis C, Leaf PJ. Multiple imputation by chained equations: what is it and how does it work? Int J Methods Psychiatr Res. 2011;20(1):40–9.

25. Schulz KF, Altman DG, Moher D. CONSORT 2010 Statement: updated guidelines for reporting parallel group randomised trials. BMJ [Internet]. 2010 Mar 24;340. Available from: http://www.bmj.com/content/340/bmj.c332.abstract

For peer review only - http://bmjopen.bmj.com/site/about/guidelines.xhtml

Anglia Ruskin University | Postgraduate Medical Institute **Clinical Trials Unit**

26. IBM. IBM SPSS Statistics for Windows, Version 22.0. Armounk, NY: IBM Corp.; 2012.

27. Cox D. Regression models and life-tables. J R Stat Soc. 1972;(34):187–220.

28. Ramsey SD, Willke RJ, Glick H, Reed SD, Augustovski F, Jonsson B, et al. Cost-Effectiveness Analysis Alongside Clinical Trials II—An ISPOR Good Research Practices Task Force Report. Value Health. 2015 Mar 1;18(2):161–72.

29. National Institute for Health and Clinical Excellence. NICE Guide to the methods of technology appraisal 2013. NICE; 2013.

30. Herdman M, Gudex C, Lloyd A, Janssen M, Kind P, Parkin D, et al. Development and preliminary testing of the new five-level version of EQ-5D (EQ-5D-5L). Qual Life Res. 2011 Dec 1;20(10):1727–36.

For peer review only

For peer review only - http://bmjopen.bmj.com/site/about/guidelines.xhtml

Anglia Ruskin University | Postgraduate Medical Institute **Clinical Trials Unit**

Appendix

	Who	When	How	To Whom
**SAE**	Chief Investigator	-Report to Sponsor within 24 hours of learning of the event<br><br>-Report to the MREC within 15 days of learning of the event	SAE Report form for Non-CTIMPs, available from NRES website.	Sponsor and MREC
**Urgent Safety Measures**	Chief Investigator	Contact the Sponsor and MREC Immediately<br><br>Within 3 days	By phone<br><br>Substantial amendment form giving notice in writing setting out the reasons for the urgent safety measures and the plan for future action.	Main REC and Sponsor<br><br>Main REC with a copy also sent to the sponsor. The MREC will acknowledge this within 30 days of receipt.
**Progress Reports**	Chief Investigator	Annually (starting 12 months after the date of favourable opinion)	Annual Progress Report Form (non-CTIMPs) available from the NRES website	Main REC
**Declaration of the conclusion or early termination of the study**	Chief Investigator	Within 90 days (conclusion)<br><br>Within 15 days (early termination)<br>*The end of study should be defined in the protocol*	End of Study Declaration form available from the NRES website	Main REC with a copy to be sent to the sponsor
**Summary of final Report**	Chief Investigator	Within one year of conclusion of the Research	No Standard Format However, the following Information should be included:-<br>Where the study has met its objectives, the main findings and arrangements for publication or dissemination including feedback to participants	Main REC with a copy to be sent to the sponsor

For peer review only - http://bmjopen.bmj.com/site/about/guidelines.xhtml

Reporting checklist for protocol of a clinical trial.

Based on the SPIRIT guidelines.

Instructions to authors

Complete this checklist by entering the page numbers from your manuscript where readers will find each of the items listed below.

Your article may not currently address all the items on the checklist. Please modify your text to include the missing information. If you are certain that an item does not apply, please write "n/a" and provide a short explanation.

Upload your completed checklist as an extra file when you submit to a journal.

In your methods section, say that you used the SPIRIT reporting guidelines, and cite them as:

Chan A-W, Tetzlaff JM, Altman DG, Laupacis A, Gøtzsche PC, Krleža-Jerić K, Hróbjartsson A, Mann H, Dickersin K, Berlin J, Doré C, Parulekar W, Summerskill W, Groves T, Schulz K, Sox H, Rockhold FW, Rennie D, Moher D. SPIRIT 2013 Statement: Defining standard protocol items for clinical trials. Ann Intern Med. 2013;158(3):200-207

		Reporting Item	Page Number
Title	#1	Descriptive title identifying the study design, population, interventions, and, if applicable, trial acronym	1
Trial registration	#2a	Trial identifier and registry name. If not yet registered, name of intended registry	3
Trial registration: data set	#2b	All items from the World Health Organization Trial Registration Data Set	n/a
Protocol version	#3	Date and version identifier	n/a
Funding	#4	Sources and types of financial, material, and other support	18
Roles and responsibilities: contributorship	#5a	Names, affiliations, and roles of protocol contributors	1 & 2
Roles and responsibilities:	#5b	Name and contact information for the trial sponsor	2

For peer review only

sponsor contact information			
Roles and responsibilities: sponsor and funder	#5c	Role of study sponsor and funders, if any, in study design; collection, management, analysis, and interpretation of data; writing of the report; and the decision to submit the report for publication, including whether they will have ultimate authority over any of these activities	n/a
Roles and responsibilities: committees	#5d	Composition, roles, and responsibilities of the coordinating centre, steering committee, endpoint adjudication committee, data management team, and other individuals or groups overseeing the trial, if applicable (see Item 21a for data monitoring committee)	n/a
Background and rationale	#6a	Description of research question and justification for undertaking the trial, including summary of relevant studies (published and unpublished) examining benefits and harms for each intervention	5, 6
Background and rationale: choice of comparators	#6b	Explanation for choice of comparators	7
Objectives	#7	Specific objectives or hypotheses	7
Trial design	#8	Description of trial design including type of trial (eg, parallel group, crossover, factorial, single group), allocation ratio, and framework (eg, superiority, equivalence, non-inferiority, exploratory)	8
Study setting	#9	Description of study settings (eg, community clinic, academic hospital) and list of countries where data will be collected. Reference to where list of study sites can be obtained	8
Eligibility criteria	#10	Inclusion and exclusion criteria for participants. If applicable, eligibility criteria for study centres and individuals who will perform the interventions (eg, surgeons, psychotherapists)	8
Interventions: description	#11a	Interventions for each group with sufficient detail to allow replication, including how and when they will be administered	9

For peer review only

Interventions: modifications	#11b	Criteria for discontinuing or modifying allocated interventions for a given trial participant (eg, drug dose change in response to harms, participant request, or improving / worsening disease)	n/a
Interventions: adherance	#11c	Strategies to improve adherence to intervention protocols, and any procedures for monitoring adherence (eg, drug tablet return; laboratory tests)	n/a
Interventions: concomitant care	#11d	Relevant concomitant care and interventions that are permitted or prohibited during the trial	9
Outcomes	#12	Primary, secondary, and other outcomes, including the specific measurement variable (eg, systolic blood pressure), analysis metric (eg, change from baseline, final value, time to event), method of aggregation (eg, median, proportion), and time point for each outcome. Explanation of the clinical relevance of chosen efficacy and harm outcomes is strongly recommended	7
Participant timeline	#13	Time schedule of enrolment, interventions (including any run-ins and washouts), assessments, and visits for participants. A schematic diagram is highly recommended (see Figure)	
Sample size	#14	Estimated number of participants needed to achieve study objectives and how it was determined, including clinical and statistical assumptions supporting any sample size calculations	8
Recruitment	#15	Strategies for achieving adequate participant enrolment to reach target sample size	8
Allocation: sequence generation	#16a	Method of generating the allocation sequence (eg, computer-generated random numbers), and list of any factors for stratification. To reduce predictability of a random sequence, details of any planned restriction (eg, blocking) should be provided in a separate document that is unavailable to those who enrol participants or assign interventions	12
Allocation concealment	#16b	Mechanism of implementing the allocation sequence (eg, central telephone; sequentially numbered, opaque, sealed	12

For peer review only

mechanism		envelopes), describing any steps to conceal the sequence until interventions are assigned	
Allocation: implementation	#16c	Who will generate the allocation sequence, who will enrol participants, and who will assign participants to interventions	n/a
Blinding (masking)	#17a	Who will be blinded after assignment to interventions (eg, trial participants, care providers, outcome assessors, data analysts), and how	
Blinding (masking): emergency unblinding	#17b	If blinded, circumstances under which unblinding is permissible, and procedure for revealing a participant's allocated intervention during the trial	
Data collection plan	#18a	Plans for assessment and collection of outcome, baseline, and other trial data, including any related processes to promote data quality (eg, duplicate measurements, training of assessors) and a description of study instruments (eg, questionnaires, laboratory tests) along with their reliability and validity, if known. Reference to where data collection forms can be found, if not in the protocol	14
Data collection plan: retention	#18b	Plans to promote participant retention and complete follow-up, including list of any outcome data to be collected for participants who discontinue or deviate from intervention protocols	14
Data management	#19	Plans for data entry, coding, security, and storage, including any related processes to promote data quality (eg, double data entry; range checks for data values). Reference to where details of data management procedures can be found, if not in the protocol	14
Statistics: outcomes	#20a	Statistical methods for analysing primary and secondary outcomes. Reference to where other details of the statistical analysis plan can be found, if not in the protocol	10, 11
Statistics: additional analyses	#20b	Methods for any additional analyses (eg, subgroup and adjusted analyses)	n/a
Statistics: analysis population and missing data	#20c	Definition of analysis population relating to protocol non-adherence (eg, as randomised analysis), and any statistical methods to handle missing data (eg, multiple imputation)	10

For peer review only

Data monitoring: formal committee	#21a	Composition of data monitoring committee (DMC); summary of its role and reporting structure; statement of whether it is independent from the sponsor and competing interests; and reference to where further details about its charter can be found, if not in the protocol. Alternatively, an explanation of why a DMC is not needed	n/a
Data monitoring: interim analysis	#21b	Description of any interim analyses and stopping guidelines, including who will have access to these interim results and make the final decision to terminate the trial	n/a
Harms	#22	Plans for collecting, assessing, reporting, and managing solicited and spontaneously reported adverse events and other unintended effects of trial interventions or trial conduct	13
Auditing	#23	Frequency and procedures for auditing trial conduct, if any, and whether the process will be independent from investigators and the sponsor	n/a
Research ethics approval	#24	Plans for seeking research ethics committee / institutional review board (REC / IRB) approval	3
Protocol amendments	#25	Plans for communicating important protocol modifications (eg, changes to eligibility criteria, outcomes, analyses) to relevant parties (eg, investigators, REC / IRBs, trial participants, trial registries, journals, regulators)	14
Consent or assent	#26a	Who will obtain informed consent or assent from potential trial participants or authorised surrogates, and how (see Item 32)	12
Consent or assent: ancillary studies	#26b	Additional consent provisions for collection and use of participant data and biological specimens in ancillary studies, if applicable	n/a
Confidentiality	#27	How personal information about potential and enrolled participants will be collected, shared, and maintained in order to protect confidentiality before, during, and after the trial	14
Declaration of interests	#28	Financial and other competing interests for principal investigators for the overall trial and each study site	18
Data access	#29	Statement of who will have access to the final trial dataset,	15

For peer review only

		and disclosure of contractual agreements that limit such access for investigators	
Ancillary and post trial care	#30	Provisions, if any, for ancillary and post-trial care, and for compensation to those who suffer harm from trial participation	n/a
Dissemination policy: trial results	#31a	Plans for investigators and sponsor to communicate trial results to participants, healthcare professionals, the public, and other relevant groups (eg, via publication, reporting in results databases, or other data sharing arrangements), including any publication restrictions	15
Dissemination policy: authorship	#31b	Authorship eligibility guidelines and any intended use of professional writers	n/a
Dissemination policy: reproducible research	#31c	Plans, if any, for granting public access to the full protocol, participant-level dataset, and statistical code	n/a
Informed consent materials	#32	Model consent form and other related documentation given to participants and authorised surrogates	n/a
Biological specimens	#33	Plans for collection, laboratory evaluation, and storage of biological specimens for genetic or molecular analysis in the current trial and for future use in ancillary studies, if applicable	n/a

The SPIRIT checklist is distributed under the terms of the Creative Commons Attribution License CC-BY-ND 3.0. This checklist can be completed online using https://www.goodreports.org/, a tool made by the EQUATOR Network in collaboration with Penelope.ai

For peer review only

BMJ Open

The Feasibility of Fingerprick Autologous Blood (FAB) As a Novel Treatment for Severe Dry Eye Disease (DED): Protocol for a Randomised Controlled Trial

Journal:	*BMJ Open*
Manuscript ID	bmjopen-2018-026770.R1
Article Type:	Protocol
Date Submitted by the Author:	25-Sep-2018

Complete List of Authors:	Balal, Shafi ; Bedford Hospital NHS Trust; Imperial College Healthcare NHS Trust<br>Udoh, Arit; Anglia Ruskin University, Clinical Trial Unit<br>Pappas, Yannis; The University of Bedfordshire,<br>Cook, Erica; University of Bedfordshire, Department of Psychology<br>Barton, Garry; University of East Anglia, Norwich Medical School<br>Hassan, Ali ; Bedford Hospital NHS Trust; Moorfields Eye Hospital NHS Foundation Trust<br>Hayden , Karen ; Anglia Ruskin University, Clinical Trial Unit, School of Medicine<br>Bourne, Rupert; Anglia Ruskin University, Vision and Eye Research Unit; Huntingdon Glaucoma Diagnostic & Research Centre at Hinchingbrooke Hospital,<br>Ahmad , Sajjad ; Moorfields Eye Hospital NHS Foundation Trust<br>Pardhan, Shahina ; Anglia Ruskin University, Vision and Eye Research Unit (VERU), School of Medicine<br>Harrison , Michael ; Anglia Ruskin University<br>Sharma, Benjamin ; Bedford Hospital NHS Trust<br>Wasil , Mohammad ; Bedford Hospital NHS Trust<br>Sharma, Anant ; Bedford Hospital NHS Trust; Moorfields Eye Hospital NHS Foundation Trust

**Primary Subject Heading**:	Ophthalmology
Secondary Subject Heading:	Evidence based practice
Keywords:	OPHTHALMOLOGY, Orbital and lacrimal disorders < OPHTHALMOLOGY, Clinical trials < THERAPEUTICS

SCHOLARONE™ Manuscripts

The Feasibility of Fingerprick Autologous Blood (FAB) As a Novel Treatment for Severe Dry Eye Disease (DED): Protocol for a Randomised Controlled Trial

Authors:

Shafi Balal[1,2]
Principal Investigator
[1]Bedford Hospital NHS Trust, Bedford, United Kingdom, and
[2]Imperial College Healthcare NHS Trust, London, United Kingdom
Email: shafi.balal@nhs.net

***Arit Udoh**[3]
Trial Coordinator
[3]Clinical Trial Unit, School of Medicine, Anglia Ruskin University, Chelmsford, United Kingdom
Email: arctu@anglia.ac.uk
Phone: 0845 196 4938

Yannis Pappas[4]
Qualitative Lead
[4]The Research Centre for Health Organisation and Delivery, Institute for Health Research, University of Bedfordshire, Luton, United Kingdom
Email: Yannis.Pappas@beds.ac.uk

Erica Cook[4]
Trial Statistician
[4]The Research Centre for Health Organisation and Delivery, Institute for Health Research, University of Bedfordshire, Luton, United Kingdom
Email: erica.cook@beds.ac.uk

Garry Barton[5]
Health Economist
[5]Norwich Medical School, University of East Anglia, Norwich, United Kingdom
Email: G.Barton@uea.ac.uk

Ali Hassan[1,6]
Research Fellow
[1]Bedford Hospital NHS Trust, Bedford, United Kingdom, and
[6]Moorfields Eye Hospital NHS Foundation Trust, London, United Kingdom
Email: Ali.Hassan@bedfordhospital.nhs.uk

Karen Hayden[3]
Clinical Trial Unit Interim Director
[3]Clinical Trial Unit, School of Medicine, Anglia Ruskin University, Chelmsford, United Kingdom
Email: karen.hayden@anglia.ac.uk

For peer review only

For peer review only - http://bmjopen.bmj.com/site/about/guidelines.xhtml

Rupert Bourne[7]
Co-Investigator
[7]Vision and Eye Research Unit (VERU), School of Medicine, Anglia Ruskin University, Cambridge, United Kingdom.
Email: rb@rupertbourne.co.uk

Sajjad Ahmad[6]
Principal Investigator
[6]Moorfields Eye Hospital NHS Foundation Trust, London, United Kingdom
Email: Sajjad.Ahmad@moorfields.nhs.uk

Shahina Pardhan[7]
Academic Lead
[7]Vision and Eye Research Unit (VERU), School of Medicine, Anglia Ruskin University, Cambridge, United Kingdom.
Email: Shahina.Pardhan@anglia.ac.uk

Michael Harrison[3]
Co-investigator
[3]Clinical Trial Unit, School of Medicine, Anglia Ruskin University, Chelmsford, United Kingdom
Email: Michael.harrisson@anglia.ac.uk

Benjamin Sharma[1]
Trial Assistant
[1]Bedford Hospital NHS Trust, Bedford, United Kingdom
Email: ruben.sharma12@gmail.com

Mohammad Wasil
Assistant Director, RDI
[1]Bedford Hospital NHS Trust, Bedford, United Kingdom
Email: mohammad.wasil@bedfordhospital.nhs.uk

Anant Sharma[1,6].
Chief Investigator
[1]Bedford Hospital NHS Trust, Bedford, United Kingdom, and
[6]Moorfields Eye Hospital NHS Foundation Trust, London, United Kingdom
Email: Anant.Sharma@bedfordhospital.nhs.uk

Trial Sponsor: Bedford Hospital NHS Trust, Bedford, United Kingdom
Word count: 3828
***Corresponding author: arctu@anglia.ac.uk**

For peer review only - http://bmjopen.bmj.com/site/about/guidelines.xhtml

ABSTRACT

Introduction

Patients with severe dry eye disease often have limited treatment options with standard non-surgical management focused on the use of artificial tears for lubrication and anti-inflammatory drugs. However, artificial tears do not address the extraordinary complexity of human tears. Crudely, human tears with its vast constituents is essentially filtered blood. Blood and several blood-derived products including autologous serum, have been studied as tears substitutes. This study proposes to test the use of whole, fresh, autologous blood obtained from a fingerprick for treatment of severe dry eye disease.

Methods and Analysis

The research team at the two participating sites will approach patients with severe dry eye disease for this study. Recruitment will take place over 12 months and we expect to recruit 60 patients in total. The primary outcome of this feasibility study is to estimate the proportion of eligible patients approached who consent to and comply with study procedures including treatment regimen and completion of required questionnaires. The secondary outcome measures, although not powered for in this feasibility, include corneal inflammation (assessed by Oxford Corneal Staining Guide), patient pain and symptoms scores (assessed by Ocular Surface Disease Index (OSDI) score), and objective signs of dry eye disease as indicated by visual acuity (assessed by Schirmer's test, tear breakup time, lower and/or upper tear meniscus height measurement). Other secondary outcomes include patients' quality of life (assessed using the validated EQ-5D-5L questionnaire), cost to the NHS and patient (assessed via use of NHS services and privately purchased over the counter treatment related to dry eyes disease) and safety measure of pressure within the eye (assessed by intra ocular pressure (IOP) score).

Ethics and Dissemination

This trial is ongoing and received a favourable research ethics opinion from the East of England - Cambridgeshire and Hertfordshire Research Ethics Committee (REC reference: 17/EE/0508).

For peer review only - http://bmjopen.bmj.com/site/about/guidelines.xhtml

The results of this study will be published in a suitable peer-review journal and also presented at international ophthalmic conferences. This will also be shared with the study participants as well as with relevant patient groups and charities.

Trial Registration Number: NCT03395431

Trial Sponsor: Bedford Hospital NHS Trust, Bedford, United Kingdom

Keywords: ophthalmology, orbital and lacrimal disorder, clinical trials

Strength and Limitation of Study

- This single-blind randomised control trial addresses a significant problem by investigating the use of whole, fresh, autologous blood obtained from a fingerprick for treatment of severe dry eye disease.
- The study intervention is cheap, simple and with minimal risk of complication.
- The intervention will allow for the delivery of the beneficial components of tears including growth factors and proteins found in whole blood.
- Patients in the intervention arm may experience soreness in fingers that are repeatedly pricked and will be advised to use a different finger for each eye.

For peer review only

INTRODUCTION

Dry eye disease (DED) is an umbrella term encompassing a range of ocular diseases estimated to affect 14% of all adults aged 48 to 91 (1). If left untreated, DED can lead to severe reduction in the quality of life of the sufferer. It can also cause loss of vision, pain in response to light, painful recurring stabbing sensations, and the feeling of grit in the affected eye(s). Studies show that moderate to severe DED can be as disruptive to patient quality of life as angina (2,3). Despite this, only less than 2% of all United Kingdom (UK) medical research funding is directed at diseases of the eye, leaving eye research critically underfunded (4). No curative agents for DED exist. The treatment of the disorder is essentially symptomatic with standard non-surgical treatment focused on the use of artificial tears for lubrication and anti-inflammatory drugs.

Anti-inflammatory agents used in DED include topical steroids and cyclosporine drops but these can cause side effects that limit their long term use (5). Topical steroids can also cause cataracts and glaucoma, all of which further limit their use. Surgical options for DED include punctal occlusion to reduce tear drainage, punctal cauterization (burning the drainage channel of tears and preventing their outflow) and partial suturing of the eyelids. However, these treatment options are associated with adverse effects and varying levels of patient tolerability. Overall, available conventional treatment options for DED often only alleviate symptoms, have limited effectiveness, and in most cases patients may fail to respond— although the exact rate of treatment failure is unavailable in the published literature.

The mainstay of non-surgical treatment for DED focuses on the use of artificial tears. These can be purchased over the counter and include brand name products such as Viscotears® and Optrex® (6). Human tears contains an extensive range of growth factors, immunoglobulins, enzymes, cytokines, vitamins and electrolytes and these have been shown in numerous studies to be essential for the maintenance and proliferation of corneal epithelial cells as well as for defence against infection (7). It is well known that artificial tears fail to account for the extraordinarily complex composition of the natural tear film. Also, many artificial tears contain preservatives that have been shown to adversely affect the cornea (8). Crudely, human tears with its vast constituents is essentially filtered blood and as such is an obvious source for a "tear mimic" containing all tear constituents. Blood and several blood-derived products including autologous serum have been studied as tears substitute candidates.

For peer review only - http://bmjopen.bmj.com/site/about/guidelines.xhtml

For peer review only

Autologous serum (AS) eye drops have been found in uncontrolled trials to be beneficial by improving the ocular surface and reducing symptoms (9–11). However, a Cochrane review concluded that there is inconsistency in evidence on its benefit (12). Obtaining autologous serum requires frequent drawing of blood from the patient— a feature that excludes patients with anaemia or heart failure from using AS. Furthermore it also appears that 100% autologous serum is more beneficial than 50% serum and requires larger volumes of blood and/or more frequent venesection (10). Patients using AS also require access to a fridge, as the product needs to be stored at low temperatures; a factor that is likely to be inconvenient for patients. In addition, AS is obtained by processing clotted blood with the initial cost to the NHS of £1653.56, and subsequent three-monthly cost of £1131.27 per patient.

The relatively high cost represents the biggest hurdle in the use of AS and is often the reason for delay or inaccessibility in its use as a treatment for DED..We propose that finger prick autologous blood is a simpler, cost-effective and possibly more acceptable method for treating dry eye disease. For this reason, this study proposes to test the use of fingerprick autologous blood (FAB) in which blood obtained from a small finger-prick is applied to the eye. Autologous fresh blood is already used in the sub-conjunctiva to help heal leaking trabeculectomy blebs (13–16). It is also used to help attach limbal autografts in cases of pterygium (17), and in vitreoretinal macular hole surgery (18–21) with no adverse effects reported. The FAB method can be used in patients who are awaiting conventional treatment for autologous blood.

Rationale and Risks/Benefits

This study proposes to test the feasibility of the use of whole, fresh, autologous blood as a treatment for severe dry eye disease (detailed study protocol presented as supplementary file). The blood can be obtained from a clean finger, pricked by the patient using a diabetic lancet and administered immediately to the eye. This allows the delivery of not only most of the aforementioned beneficial components of tears but additional growth factors and proteins, fresh and unprocessed, which can help heal the ocular surface. If validated, this may replace current autologous serum practice and its ease of use, vastly reduced cost with greater convenience may mean that it could be extended to other ocular surface diseases. The proposing team have completed an exploratory study on the use of fingerprick autologous blood (FAB) for persistent epithelial defects and severe dry eye disease and preliminary

For peer review only - http://bmjopen.bmj.com/site/about/guidelines.xhtml

results indicate improvement with no adverse events reported (22). The exploratory study included 16 patients with a diagnosis of severe to moderate dry eye syndrome and used the FAB method for treatment. The findings of the study demonstrated mean improvements in visual acuity, Oxford corneal staining grade, tear breakup time, Schirmer's test and dry eye disease questionnaire score. The response rate from participants was good with only a single patient who met the inclusion criteria not wishing to participate in the trial. Both the amount of staining (indicating inflammation and ocular surface damage) and their DED questionnaire scores (indicating severity of their symptoms and impact on quality of life) showed mean improvement which reached statistical significance. A search of clinical databases (Medline, CINAHL and AMED) including on-going trials on the UK CRN portfolio database and related websites (www.controlled-trials.com) did not identified any studies using FAB as a treatment for dry eye disease.

PRIMARY AND SECONDARY OBJECTIVES

The primary objective is to determine the feasibility of a definitive randomised controlled trial (RCT) to evaluate effectiveness of the use of fresh autologous blood (FAB) compared to conventional treatment for patients with severe dry eye syndrome. The primary outcome is the proportion of eligible patients approached who consent to and comply with study procedure including treatment regimen and completion of required questionnaires. The secondary objectives, although not powered for within this feasibility include: to estimate the effectiveness of the trial intervention and further explore the acceptability of the study design, and to explore the feasibility of collecting resource use and quality of life data to inform the design of the health economics component of a future definitive trial. The secondary outcome measures, although not powered for within this feasibility, include corneal inflammation (assessed by Oxford Corneal Staining Guide), patient pain and symptoms scores (assessed by Ocular Surface Disease Index (OSDI) score), and objective signs of dry eye disease as indicated by visual acuity (assessed by Schirmer's test, tear breakup time, lower and/or upper tear meniscus height measurement). Other secondary outcomes include patients' quality of life (assessed using the validated EQ-5D-5L questionnaire), cost to the NHS and patient (assessed via use of NHS services and privately purchased over the counter treatment related to dry eyes disease) and safety measure of pressure within the eye (assessed by intra ocular pressure (IOP) score).

For peer review only - http://bmjopen.bmj.com/site/about/guidelines.xhtml

METHOD

Study Design

This is a single blind, two-arm feasibility randomised controlled trial of FAB with conventional treatment versus conventional therapy alone for severe dry eye disease, including a qualitative process evaluation (Figure 1 provides details of the study scheme). This trial is ongoing and open to recruitment at two participating sites: Bedford Hospital NHS Trust and Moorfields Eye Hospital London. Recruitment into the trial commenced in April 2018 and is due to finish by April 2019.

Sample Size

As this is a feasibility trial, there was no formal sample size calculations (23). The aim of the study is to recruit sufficient patients to evaluate the acceptability and feasibility of the intervention. An outcome of this study will be to estimate parameters such as the standard deviation for a sample size calculation of a subsequent full-scale trial. A sample of 52 patients (26 in each arm) is considered adequate for obtaining reliable sample size estimates (24, 25). To allow for a conservative attrition rate of 10%, we aim to recruit 60 patients into the trial.

Participant Selection

Inclusion Criteria

- Patient age ≥ 18 years
- Severe symptomatic dry eye disease diagnosed by: Ocular surface disease index (OSDI) score of greater than 33; OR Oxford Corneal Staining grade 2 or greater; OR Schirmer's without anaesthesia <5mm at 5 minutes
- Patients on artificial tears and/or lubricating drops/gel four times a day
- Patient able to give consent
- Patients able and willing to complete the quality of life (QoL) questionnaires required for the study.

Exclusion Criteria

- Fear of needles
- Unable or not willing to carry out repeat finger pricks
- Patients with infected finger/s or systemic infection or on systemic antibiotics for infection.

For peer review only - http://bmjopen.bmj.com/site/about/guidelines.xhtml

- Patients with active ocular infection, active immunological corneal melt, or recurrent corneal erosion.
- Pregnant or breast feeding women
- Previous use of FAB treatment (e.g. from exploratory study)
- Systemic illness causing immune system deficiency
- Graft versus host disease
- Previous use of autologous serum within 3 months
- Diabetes

Intervention

- ***Arm A – FAB plus conventional treatment***

The patients will use FAB therapy alongside conventional therapy (artificial tears, cyclosporine drops or punctal plugs/cautery) as recommended by their treating ophthalmologist. A fingertip of the hand will be wiped with an alcohol steret and self-pricked using a standard diabetic lancet. The drop of blood is produced as normal and applied to the lower fornix of the affected eye(s) with the lower lid pulled down slightly by the patient. The blood will be applied four times a day. A fresh finger will be used for each eye and blood wiped away with the alcohol steret. Patients will be advised to keep nails short and nail varnish avoided. FAB will be applied at least 15 minutes after any artificial tears and no drops for at least half an hour afterwards.

- ***Arm B – Conventional treatment only***

The patients will use conventional therapy (artificial tears, cyclosporin drops and punctal plugs/cautery) as recommended by their treating ophthalmologist.

Qualitative Approach

A nested qualitative approach using in-depth, semi-structured interviews will help us understand the lived experience of people using FAB and factors relating to the ways that clinical departments and healthcare professionals adapt to working with FAB. Topics, questions and probes in the interviews will emerge from three sources: the relevant literature; our experience of and involvement in clinical ophthalmology; and consultation with patients. In this feasibility study, we will use a convenience sampling technique for the qualitative work. We will interview all clinicians who administer the intervention in the study and we will aim to interview 10 patients (adjusted for setting, gender and age group representation). Given the resources, we will aim to draw findings from a larger sample in a future study. This phase will work in parallel to the quantitative data collection so that emerging themes can be

For peer review only - http://bmjopen.bmj.com/site/about/guidelines.xhtml

investigated in later interviews. Even though the feasibility study sample is reasonably limited, we anticipate some useful insights about the use of FAB to occur because of the triangulation between the RCT and the qualitative findings (for example, reasons for partial adherence).

Economic Evaluation

Good practice recommendations for cost-effectiveness analyses suggest concentrating on the measurement of large cost drivers, with less focus on resources that are not expected to differ between different treatments (26). Estimation of cost-effectiveness is therefore an iterative process and by including a health economic component in a feasibility it is possible to consider how the methods might be refined in any future definitive study. In order to estimate costs, informed by previous data collection instruments (Database of Instruments for Resource Use Measurement: http://www.dirum.org/) and NICE guidance (27), a self-report resource use questionnaire will be devised. This will capture the use of NHS services and privately purchased over the counter treatments such as OPTREX. For benefits, the EQ-5D-5L (28) will be used to measure quality of life, enabling QALY (Quality Adjusted Life Year) scores to be calculated. The main purpose of the economic analysis is to inform the decision regarding how and what cost and effect data would be collected within a more definitive study. In order to inform this decision, we will estimate completion rates and seek to identify big cost drivers.

Outcome Assessment

The primary outcome measure is the proportion of eligible patients approached who consent to and comply with the trial protocol. This will be assessed by: number of eligible patients within the study population and recruitment timeframe, percentage of eligible patients accepted for randomisation, patient compliance with trial protocol measured as per self-reported adherence to intervention, and percentage of patients completing study. The secondary outcomes include reduction in corneal inflammation (assessed by Oxford Corneal Staining Guide), patient pain and symptoms scores (assessed by Ocular Surface Disease Index (OSDI) score), improvement in objective signs of dry eye disease as indicated by visual acuity (assessed by Schirmer's test, tear breakup time, lower and/or upper tear menisci height measurement). Other secondary endpoints include patients' quality of life (assessed using the EQ-5D-5L questionnaire), cost to the NHS and patient (assessed via use of

For peer review only - http://bmjopen.bmj.com/site/about/guidelines.xhtml

additional NHS services and privately purchased over the counter treatments related to dry eyes disease) and safety measure of pressure within the eye (assessed by Intra ocular pressure (IOP) score).

Data Analysis

A detailed data validation will examine completeness, existence and accuracy of collected data to assess data quality and identify missing and conflicting data. The statistical analysis will be performed on an intention-to-treat basis (i.e. inclusion of patients randomly assigned, regardless of adherence, actual treatment received, and subsequent withdrawal of treatment and/or deviation from the protocol and per-protocol (i.e. inclusion of those who completed the treatment as planned) (29) and will be reported according to 2010 CONSORT guidelines (30). All statistical analyses will be completed using SPSS Statistics v.22.0 (31). A p-value of <0.05 will be accepted as statistical significance. Conforming to recommendations for feasibility studies, data analysis will be primarily descriptive, with means, standard deviations and frequency counts calculated for all variables of interest.

Exploratory efficacy analysis will compare the primary outcome variables derived from the data collected at 8 weeks between the two arms using a marginal general linear model (GLM) with robust standard errors, to allow for clustering by group. Secondary outcomes between the intervention and control groups will be compared at 4 weeks, 8 weeks and 12 weeks (4 weeks post treatment). Safety outcome measures will include IOP rise and any reported infection. Secondary analyses of the primary outcome, controlling for baseline covariates, will be performed using proportional hazards regression models (32). Analysis of treatment discontinuation adjusted for clinical site, baseline status, and whether the patient is randomized to the FAB or control (conventional treatment only) group will be conducted. Any interaction between baseline status or regimen and treatment will also be assessed. Interactions between significant covariates and treatment effects will be assessed in the context of the proportional hazards models.

Interviews will be recorded, transcribed and analysed using the framework analysis. We will structure the analysis of collected data to fit two evaluation frameworks relevant to the successful implementation of FAB:

For peer review only

For peer review only - http://bmjopen.bmj.com/site/about/guidelines.xhtml

- Patient-oriented: Understand the lived experience of patients using FAB with emphasis on intervention acceptability, perceived enablers and barriers for adherence, perceived clarity of advice, and guidance and factors relating to the initial uptake of the intervention.
- Organisation-oriented: Whether and how the use of FAB affected workload and/or workflow; identify process change, adaptation challenges, skills gap; establish treatment fidelity and clinician acceptability.

Study Procedures

Informed Consent Procedures

It is the responsibility of the Investigator, or appropriately GCP trained person delegated by the Investigator as documented in the site delegation log, to obtain written informed consent from each participant prior to any participation/study specific procedures. This should follow adequate explanation of the aims, methods, anticipated benefits and potential hazards of the study. The participants will be given ample time to consider giving their consent for the study. For this study a minimum of 24 hours will be given during which the consenting team will be contactable over the phone to answer any questions. The date that the Participant Information Sheet (PIS) is given to the participant must be documented within the patient's notes to confirm that sufficient time was given. The Investigator (or other qualified person) will explain to the potential participant that they are free to refuse any involvement within the study or alternatively withdraw their consent at any point during the study and for any reason. If there is any further safety information which may result in significant changes in the risk/benefit analysis, the PIS and Informed Consent Form (ICF) will be reviewed and updated accordingly. All participants that are actively enrolled on the study will be informed of the updated information and given a revised copy of the PIS/ICF in order to confirm their wish to continue on the study.

Screening Procedures

Patients will have undergone the below procedures as per standard of care which will in turn feed in to the investigator for their review prior to approaching the potential participant.

- Ocular surface disease index (OSDI) score of greater than 33; OR
- Oxford Corneal Staining grade 2 or greater; OR
- Schirmer's without anaesthesia <5mm at 5 minutes

For peer review only - http://bmjopen.bmj.com/site/about/guidelines.xhtml

The results of these which will be reviewed by investigator prior to approaching the potential participant.

Randomisation Procedures

Randomisation will be carried out by the Anglia Ruskin Clinical Trials Unit (ARCTU) using the SEALED ENVELOPE randomisation service. SEALED ENVELOPE is a randomisation and online database service developed for clinical trial services. It is an internet based system and will be set for this study by ARCTU in accordance with the protocol. Enrolled patients will undergo 1:1 block randomisation to Arm A or B. The system stores the pre-determined sequence of randomisation and this list is visible to neither the investigator nor ARCTU staff. Once a patient has consented to take part in the trial, they will be randomly allocated to either Arm A to receive FAB and convention therapy or Arm B to receive conventional therapy only. The Research Nurse or Fellow or designated staff will log on to a web browser application and enter the patient's eligibility factors into the system. The treatment allocation will be sent to the research team who will make the necessary arrangements for the patient's treatment plan.

The treating ophthalmologist in clinic will prescribe and counsel all patients on the correct technique for conventional treatment. The unblinded research nurses will provide additional training to the patients on the method of delivering FAB, including the use of a training video. Patients will be advised by the research team to inform their ophthalmologist if they have been started on any new treatment during the trial period by other clinicians or themselves.

End of Study Definition

The definition of the end of study will be the point where the last patient recruited had the last follow-up visit.

Participant Withdrawal

Patients will be withdrawn from study based on the following:

- New diagnosis of infection on finger or eye
- Requesting to be withdrawn for other reasons such as inconvenience of increased clinic visits
- Pregnancy after recruitment
- Systemic infection, or systemic antimicrobials for infection.

For peer review only - http://bmjopen.bmj.com/site/about/guidelines.xhtml

- If finger sore from repeated prick and patient does not want to use another finger.

Any adverse effects will be reported back to the lead investigator at the site and if necessary patients

Notification and Reporting Adverse Events or Reactions

No serious adverse events (SAEs) or complications associated with use of fingerprick autologous blood in the treatment of ocular disease has been reported by the literature. Preliminary results of a pilot study conducted by the authors and involving 16 patients with dry eye disease also demonstrated no adverse effects of using FAB in the eye for two months (22). Although, we do not envisage any serious adverse events with the intervention in this trial, if an adverse event (AE) occurs and is not defined as SERIOUS, the AE will be recorded in the study file and the participant followed up by the research team. The AE will also be documented in the participants' medical notes (where appropriate) and the CRF. Serious Adverse Event (SAEs) that are considered to be 'related' and 'unexpected' will be reported to the sponsor within 24 hours of learning of the event and to the Main REC within 15 days in line with the required timeframe. SAE reporting will commence at the start of the trial and up to 4 weeks after the intervention.

PATIENT AND PUBLIC INVOLVEMENT

Patients who participated in a previous pilot (exploratory) study conducted by the study authors (22) were involved in a focus group meeting and provided inputs on research design and study procedures. Two of the patients also participated in an advisory group for this trial provided input on prioritising the research questions and also reviewed the study protocol and participant information resources. Patients in this trial will be invited to request study results from the research team if interested.

ETHICS AND DISSEMINATION

Participant anonymity will be protected and maintained at all times. Information with regards to study participants will be kept confidential and managed in accordance with the Data Protection Act, NHS Caldicott Guardian, The Research Governance Framework for Health and Social Care and Research Ethics Committee Approval in United Kingdom. Data obtained in the trial will be stored using a unique study code, which is non-identifiable and anonymised. Patient's personal data will be stored separately and confidentially by the

For peer review only - http://bmjopen.bmj.com/site/about/guidelines.xhtml

participating sites and not entered into the trial database. All written information will stored in a locked filing cabinet in a locked room. Any web-based data will be stored in a secure password protected and encrypted central database at the Anglia Ruskin Clinical Trials Unit. Only individuals directly involved with the study will have access to trial data. This protocol and any subsequent amendments, along with any accompanying material provided to the participant in addition to any advertising material used in this trial have been approved by the East of England - Cambridgeshire and Hertfordshire Research Ethics Committee (REC reference: 17/EE/0508). Written Approval from the Committee was obtained and subsequently submitted to the respective Trust's Research and Development office with final NHS R&D approval obtained. Data obtained from this study will be published in a suitable peer-review journal and also presented at international ophthalmic conferences including American Academy of Ophthalmology, Royal College of Ophthalmology annual congress, Association for Research and Vision and Ophthalmology, and the European Society of Cataract and Refractive Surgery. Information will be provided to patient groups and charities such as the Sjogren's society and the Royal National Institute of Blind People. Study participants will also be provided with the contact details of the trial team at the respective sites who will be able to answer any additional queries.

For peer review only

For peer review only - http://bmjopen.bmj.com/site/about/guidelines.xhtml

REFERENCES

1. Moss SE, Klein R, Klein BK. Prevalence of and risk factors for dry eye syndrome. *Arch Ophthalmol*. 2000 Sep 1;118(9):1264–8.

2. Hom MM, Nguyen A, Bielory L. Allergic conjuctivitis and dry eye syndrome. Ann *Allergy Asthma Immunol Off Publ Am Coll Allergy Asthma Immunol*. 2012;108:163–6.

3. Schiffman RM, Walt JG, Jacobsen G, Doyle JJ, Lebovics G, Sumner W. Utility assessment among patients with dry eye disease. *Ophthalmol*. 2003;110(7):1412–9.

4. National Eye Research Centre. Eye research overview [Internet]. 2016. Available from: https://www.nerc-charity.org.uk/our-eye-research

5. Kymionis GD, Bouzoukis DI, Diakonis VF, Siganos C. Treatment of chronic dry eye: focus on cyclosporine. *Clin Ophthalmol Auckl NZ*. 2008 Dec;2(4):829–36.

6. Diebold Y, Herreras JM, Callejo S, Argueso P, Calonge M. Carbomer- Versus Cellulose-Based Artificial-Tear Formulations: Morphologic and Toxicologic Effects on a Corneal Cell Line. *Cornea*. 1998;17(4):433.

7. Yokoi N, Yamada H, Mizukusa Y, Bron AJ, Tiffany JM, Kato T, et al. Rheology of Tear Film Lipid Layer Spread in Normal and Aqueous Tear–Deficient Dry Eyes. *Invest Ophthalmol Vis Sci*. 2008 Dec 1;49(12):5319–24.

8. Pfister R, Burstein N. The effects of opthalmic drugs, vehicles and preservatives on corneal epithelium: a scanning electron microscope study. *Invest Ophthalmol Vis Sci*. 1976;15(4):246–59.

9. Fox R, Chan R, Michelson J, Belmont J, Michelson P. Beneficial effect of artificial tears made with artificial tears made with autologous serum in patients with keratoconjunctivitis sicca. *Arthritis Rheumatol*. 1984;29:577–83.

10. Poon AC, Geerling G, Dart JKG, Fraenkel GE, Daniels JT. Autologous serum eyedrops for dry eyes and epithelial defects: clinical and in vitro toxicity studies. *Br J Ophthalmol*. 2001 Oct 1;85(10):1188.

11. Tsubota K, Goto E, Fujuta H, Ono M, Inuoue H, Saito I. Treatment of dry eye by autologous serum application in Sjogren's Syndrome. *Br J Ophthalmol*. 1999;83:390–5.

12. Pan Q, Angelina A, Zambrano A, Marrone M, Stark WJ, Heflin T, et al. Autologous serum eye drops for dry eye. Cochrane Database Syst Rev [Internet]. 2013;(8). Available from: http://dx.doi.org/10.1002/14651858.CD009327.pub2

13. Biswas S, Zaheer I, Monsalve B, Diamond JP. Compression sutures with autologous blood injection for leaking trabeculectomy blebs. *Br J Ophthalmol*. 2009 Mar 25;93(4):549.

14. Burnstein A, WuDunn D, Ishii Y, Jonescu-Cuypers C, Cantor LB. Autologous blood injection for late-onset filtering bleb leak. *Am J Ophthalmol*. 2001 Jul 1;132(1):36–40.

For peer review only - http://bmjopen.bmj.com/site/about/guidelines.xhtml

15. Dinah C, Bhachech B, Ghosh G. Long-term success with autologous blood injection for leaking trabeculectomy blebs. *Br J Ophthalmol.* 2010 Mar 9;94(3):392.

16. Haynes WL, Alward WLM. Combination of Autologous Blood Injection and Bleb Compression Sutures to Treat Hypotony Maculopathy. *J Glaucoma* [Internet]. 1999;8(6). Available from: http://journals.lww.com/glaucomajournal/Fulltext/1999/12000/Combination_of_Autologous_Blood_Injection_and_Bleb.8.aspx

17. de Wit D, Athanasiadis I, Sharma A, Moore J. Sutureless and glue-free conjunctival autograft in pterygium surgery: a case series. *Eye*. 2010 Sep;24(9):1474–7.

18. Chuang L-H, Wang N-K, Yeung L, Chen Y-P, Hwang Y-S, Wu W-C, et al. Use of autologous whole blood during internal limiting membrane peeling and macular hole surgery is protective for indocyanine green toxicity. *Cutan Ocul Toxicol*. 2010 Jun 1;29(2):98–104.

19. Lai C-C, Hwang Y-S, Liu L, Chen K-J, Wu W-C, Chuang L-H, et al. Blood-Assisted Internal Limiting Membrane Peeling for Macular Hole Repair. *Ophthalmol*. 2009 Aug 1;116(8):1525–30.

20. Lai C-C, Wu W-C, Chuang L-H, Yeung L, Chen T-L, Lin K-K. Prevention of Indocyanine Green Toxicity on Retinal Pigment Epithelium with Whole Blood in Stain-Assisted Macular Hole Surgery. *Ophthalmol*. 2005 Aug 1;112(8):1409–14.

21. McCannel CA. Heparinized Autologous Blood for Visualization of the Internal Limiting Membrane During Macular Hole Surgery. *Retina* [Internet]. 2008;28(2). Available from: http://journals.lww.com/retinajournal/Fulltext/2008/02000/Heparinized_Autologous_Blood_for_Visualization_of.22.aspx

22. Than J, Balal S, Wawrzynski J, Nesaratnam N, Saleh GM, Moore J, et al. Fingerprick autologous blood: a novel treatment for dry eye syndrome. *Eye*. 2017 Jun 16;31:1655.

23. Lancaster GA, Dodd S, Williamson PR. Design and analysis of pilot studies: recommendations for good practice. *J Eval Clin Pract*. 2004 Jun 4;10(2):307–12.

24. Sim J, Lewis M. The size of a pilot study for a clinical trial should be calculated in relation to considerations of precision and efficiency. *J Clin Epidemiol.* 2012 Mar 1;65(3):301–8.

25. Julious SA. Sample size of 12 per group rule of thumb for a pilot study. *Pharm Stat.* 2005 Nov 24;4(4):287–91.

26. Ramsey SD, Willke RJ, Glick H, Reed SD, Augustovski F, Jonsson B, et al. Cost-Effectiveness Analysis Alongside Clinical Trials II—An ISPOR Good Research Practices Task Force Report. *Value Health*. 2015 Mar 1;18(2):161–72.

For peer review only - http://bmjopen.bmj.com/site/about/guidelines.xhtml

27. National Institute for Health and Clinical Excellence. NICE Guide to the methods of technology appraisal 2013 [Internet]. NICE; 2013. Available from: https://www.nice.org.uk/process/pmg9/chapter/introduction

28. Herdman M, Gudex C, Lloyd A, Janssen M, Kind P, Parkin D, et al. Development and preliminary testing of the new five-level version of EQ-5D (EQ-5D-5L). *Qual Life Res.* 2011 Dec 1;20(10):1727–36.

29. Fisher LD, Dixon DO, Herson J, Frankowski RK, Hearron MS, Peace KE. Intention to treat in clinical trials. In: Statistical issues in drug research and development. New York: Marcel Dekker; 1990. p. 331–50.

30. Schulz KF, Altman DG, Moher D. CONSORT 2010 Statement: updated guidelines for reporting parallel group randomised trials. *BMJ* [Internet]. 2010 Mar 24;340. Available from: http://www.bmj.com/content/340/bmj.c332.abstract

31. IBM. IBM SPSS Statistics for Windows, Version 22.0. Armounk, NY: IBM Corp.; 2012.

32. Cox D. Regression models and life-tables. *J R Stat Soc*. 1972;(34):187–220.

Authors' Contribution: SB and AS conceived the study with input from YP, EC, GB, MH, RB, SA, BS, MW and SP. AU, KH and MH developed the study protocol with statistical input from EC, qualitative design from YP and input on the economic evaluation phase from GB. AU and AH reviewed the draft protocol for intellectual content with final review and approval by all the authors.

Funding Statement: This work was supported by NIHR Research for Patient Benefit grant number PB-PG-0416-20040.

Ethics Approval: Ethical approval for this study has been granted by the East of England - Cambridgeshire and Hertfordshire Research Ethics Committee (REC reference: 17/EE/0508).

Competing interest: None declared

Data sharing statement: We shall make data available to the scientific community with as few restrictions as feasible, while retaining exclusive use until the publication of major outputs Available and anonymised data can be obtained by contacting the corresponding author.

For peer review only - http://bmjopen.bmj.com/site/about/guidelines.xhtml

List of Figures

Figure 1: Study Scheme

For peer review only

For peer review only - http://bmjopen.bmj.com/site/about/guidelines.xhtml

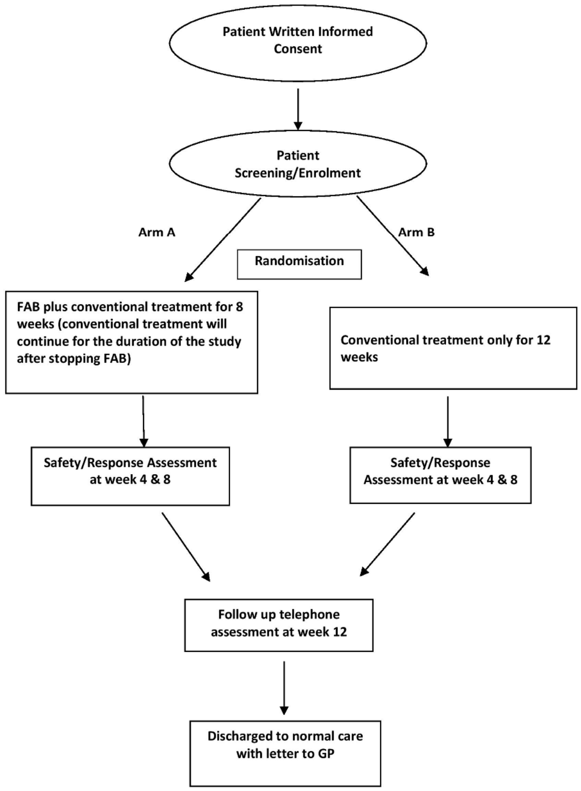

Figure 1: Study Scheme

Figure 1: Study Scheme

210x297mm (300 x 300 DPI)

Anglia Ruskin University | Postgraduate Medical Institute Clinical Trials Unit

The Feasibility of Fingerprick Autologous Blood (FAB) As a Novel Treatment for Severe Dry Eye Disease (DED): A Randomised Controlled Trial

Short title/Acronym: FAB in DED Trial

Sponsor: Bedford Hospital NHS Trust

Representative of the Sponsor:
Dr Mohammad Wasil
Assistant Director, RDI
Bedford Hospital NHS Trust
Kempston Road
Bedford
MK42 9DJ

REC reference: 17/EE/0508

IRAS Project ID: 233813

For peer review only - http://bmjopen.bmj.com/site/about/guidelines.xhtml

Postgraduate Medical Institute
Clinical Trials Unit

Chief Investigator Agreement Page

The clinical study as detailed within this research protocol **(Version 1.4, dated 10 July 2018)** or any subsequent amendments will be conducted in accordance with the Research Governance Framework for Health & Social Care (2005), the World Medical Association Declaration of Helsinki (1996) and the current applicable regulatory requirements and any subsequent amendments of the appropriate regulations.

Chief Investigator Name: Mr Anant Sharma

Chief Investigator Site: Bedford Hospital NHS Trust

Signature and Date:

For peer review only

For peer review only - http://bmjopen.bmj.com/site/about/guidelines.xhtml

Anglia Ruskin University | Postgraduate Medical Institute **Clinical Trials Unit**

Statistician Agreement Page

The clinical study as detailed within this research protocol **(Version 1.4, dated 10 July 2018)** or any subsequent amendments will be conducted in accordance with the Research Governance Framework for Health & Social Care (2005), the World Medical Association Declaration of Helsinki (1996) and the current applicable regulatory requirements and any subsequent amendments of the appropriate regulations.

Statistician Name: Dr Erica Cook

Site: University of Bedfordshire, Bedford

Signature and Date:

For peer review only - http://bmjopen.bmj.com/site/about/guidelines.xhtml

Postgraduate Medical Institute
Clinical Trials Unit

Principal Investigator Agreement Page

The clinical study as detailed within this research protocol **(Version 1.4, dated 10 July 2018)** or any subsequent amendments will be conducted in accordance with the Research Governance Framework for Health & Social Care (2005), the World Medical Association Declaration of Helsinki (1996) and the current applicable regulatory requirements and any subsequent amendments of the appropriate regulations.

Principal Investigator Name:

Principal Investigator Site:

Signature and Date:

For peer review only - http://bmjopen.bmj.com/site/about/guidelines.xhtml

Anglia Ruskin University | Postgraduate Medical Institute **Clinical Trials Unit**

Study Summary/Synopsis

**TITLE**	The Feasibility of Fingerprick Autologous Blood (FAB) as a Novel Treatment for Severe Dry Eye Disease (DED): A Randomised Controlled Trial
**SHORT TITLE**	FAB in DED Trial
**Protocol Version Number and Date**	**Version 1.4, dated 10 July 2018**
**Methodology**	Single-blind randomised controlled Trial
**Study Duration**	24months
**Study Centre**	Bedford Hospital NHS Trust, Moorfields Eye Hospital London,
**Objectives**	To determine the feasibility of a definitive randomised controlled trial (RCT) to determine effectiveness of the use of fresh autologous blood (FAB) compared to conventional treatment for patients with dry eye syndrome.
**Number of Participants**	60
**Main Inclusion Criteria**	- Patient age > 18 years<br>- Severe symptomatic dry eye disease diagnosed by: Ocular Surface Disease Index (OSDI) score of greater than 33; OR Oxford Corneal Staining grade 2 or greater; OR Schirmer's without anaesthesia <5mm at 5 minutes.<br>- Patient able to consent<br>- Patients able and willing complete the quality of life (QoL) questionnaires required for the study
**Statistical Methodology and Analysis**	Data analysis will be primarily descriptive with means, standard deviations and frequency counts calculated for all variables of interest. Exploratory statistical analysis will compare the primary outcome and secondary outcomes between the intervention and control groups at 4 weeks, 8 weeks and 12 weeks (4 weeks post treatment)

For peer review only - http://bmjopen.bmj.com/site/about/guidelines.xhtml

Anglia Ruskin University | Postgraduate Medical Institute Clinical Trials Unit

Glossary of Terms and Abbreviations

AE	Adverse Event
AR	Adverse Reaction
ASR	Annual Safety Report
CA	Competent Authority
CI	Chief Investigator
CRF	Case Report Form
CRO	Contract Research Organisation
DMC	Data Monitoring Committee
EC	European Commission
GAfREC	Governance Arrangements for NHS Research Ethics Committees
ICF	Informed Consent Form
ISRCTN	International Standard Randomised Controlled Trial Number
MA	Marketing Authorisation
MS	Member State
Main REC	Main Research Ethics Committee
NHS R&D	National Health Service Research & Development
PI	Principle Investigator
QA	Quality Assurance
QC	Quality Control
Participant	An individual who takes part in a clinical trial
RCT	Randomised Controlled Trial
REC	Research Ethics Committee
SAE	Serious Adverse Event
SDV	Source Document Verification
SOP	Standard Operating Procedure
SSA	Site Specific Assessment
TMG	Trial Management Group
TSC	Trial Steering Committee

For peer review only - http://bmjopen.bmj.com/site/about/guidelines.xhtml

Anglia Ruskin University | Postgraduate Medical Institute **Clinical Trials Unit**

Table of Contents

1 Introduction 9
1.1 Background 9
1.2 Rationale and Risks/Benefits 11
2 Trial Objectives and Design 12
2.1 Primary Objective and Endpoints 12
2.2 Secondary Objective and Endpoints 12
2.3 Trial Design 13
2.3.1 Setting 14
2.4 Study Scheme Diagram 15
3 Participant Selection 16
3.1 Inclusion Criteria 16
3.2 Exclusion Criteria 16
4 Study Procedures 17
4.1 Informed Consent Procedures 17
4.2 Screening Procedures 17
4.3 Randomisation Procedures 18
4.4 Baseline Procedures 18
4.5 Schedule of Assessment 19
4.6 End of Study Definition 20
4.7 Participant Withdrawal 20
5 Pharmacovigilance 21
5.1 General Definitions 21
5.1.1 Adverse Event (AE) 21
5.1.2 Serious Adverse Event (SAE) 21
5.2 Investigators Assessment 21
5.2.1 Seriousness 21
5.2.2 Causality 21
5.2.3 Expectedness 21
5.2.4 Severity 22
5.3 Notification and reporting Adverse Events or Reactions 22
5.4 Notification and Reporting of Serious Adverse Events 22
5.5 Urgent Safety Measures 22
5.6 Annual Safety Reporting 23
5.7 Overview of the Safety Reporting Process/Pharmacoviligance responsibilities 23
6 Statistical Considerations 24
6.1 Qualitative approach 25
7 Data Handling & Record Keeping 27
7.1 Confidentiality 27
7.2 Study Documents 27
7.3 Case Report Form 28
7.4 Record Retention and Archiving 28
7.5 Compliance 28
7.6 Clinical Governance Issues 29
7.6.1 Ethical Considerations 29

For peer review only - http://bmjopen.bmj.com/site/about/guidelines.xhtml

Anglia Ruskin University | Postgraduate Medical Institute **Clinical Trials Unit**

7.7 Quality Control and Quality Assurance 29
7.7.1 Summary Monitoring Plan 29
7.7.2 Audit and Inspection 29
7.8 Non-Compliance 30
8 Trial Committees 31
9 Publication Policy 32
References 33
Appendix 36

For peer review only

For peer review only - http://bmjopen.bmj.com/site/about/guidelines.xhtml

Anglia Ruskin University | Postgraduate Medical Institute **Clinical Trials Unit**

1 Introduction

1.1 Background

Dry eye disease (DED) is an umbrella term encompassing a range of diseases estimated to affect 14% of all adults aged 48 to 91. If left untreated, DED can lead to severe reduction in the quality of life of the sufferer. It can also cause loss of vision, pain in response to light, painful recurring stabbing sensations, and the feeling of grit in the affected eye(s). Studies show that moderate to severe DED can be as disruptive as angina (1,2). Despite this, only less than 2% of all UK medical research funding is directed at diseases of the eye, leaving eye research critically underfunded (3).

No curative agents for DED exist. The treatment of the disorder is essentially symptomatic with standard non-surgical treatment focused on the use of artificial tears for lubrication and anti-inflammatory drugs. Anti-inflammatory agents include topical steroids and ciclosporin drops but these can cause side effects that limit their long term use (4). Topical steroids can also cause cataracts and glaucoma, all of which further limit its use. Surgical options for DED include punctal occlusion to reduce tear drainage, punctal cauterization (burning the drainage channel of tears, preventing their outflow) and partial suturing of the eyelids; but these are associated with adverse effects and varying levels of patient tolerability. Overall, available conventional treatment options for DED often only alleviate symptoms, have limited effectiveness, and in most cases patients may fail to respond; although the exact rate of treatment failure is unavailable in the published literature.

The mainstay of non-surgical treatment for DED focuses on the use of artificial tears. These can be purchased over the counter and include brand name products such as Viscotears® and Optrex® (5). Human tears contains an extensive range of growth factors, immunoglobulins, enzymes, cytokines, vitamins and electrolytes and these have been shown in numerous studies to be essential for the maintenance and proliferation of corneal epithelial cells as well as for defence against infection (6). It is well known that artificial tears fails to account for the extraordinarily complex composition of the natural tear film. Also, many artificial tears contain preservatives that have been shown to adversely affect the cornea (7). Crudely, human tears with its vast constituents is essentially filtered blood and as such is an obvious source for a

For peer review only - http://bmjopen.bmj.com/site/about/guidelines.xhtml

"tear mimic" containing the substances of tears. Blood, and several blood derived products, including autologous serum, have been studied as tear substitute candidates.

Autologous serum (AS) eye drops have been found in uncontrolled trials to be beneficial in DED patient by improving the ocular surface and reducing symptoms (8–10). However, a Cochrane review concluded that there is inconsistency in evidence on its benefit (11). Obtaining autologous serum requires frequent drawing of blood from the patient— a feature that excludes patients with anaemia or heart failure from using AS. Furthermore it also appears that 100% autologous serum is more beneficial than 50% serum and requires larger volumes of blood and/or more frequent venesection (9). Patients using AS also require access to a fridge as the product needs to be stored at low temperatures; a factor that is likely to be inconvenient for patients. In addition, AS is obtained by processing clotted blood which is often too expensive for the health service to consistently purchase, given the initial cost of £1653.56 and subsequent three-monthly cost of £1131.27 per patient.

The relatively high cost represents the biggest hurdle in the use of AS and is often the reason for delay or inaccessibility in starting treatment for DED using AS. However, we propose that finger prick autologous blood may be a simpler, cost-effective and possibly more acceptable method for treating dry eye disease. For this reason, this study proposes to test the use of finger prick autologous blood (FAB) technique in which whole blood is applied to the eye from a cleaned finger. Autologous fresh blood is already used subconjunctivally to help heal leaking trabeculectomy blebs (12–15). It is also used to help attach limbal autografts in cases of pterygium (16) and vitreoretinal macular hole surgery (17–20), with no adverse effects reported. The FAB method can be used immediately for patients who are awaiting conventional treatment for autologous blood. The objective of this study is therefore to investigate if serum via a drop of fresh blood is an effective treatment for severe dry eye disease which currently require venesected autologous blood and whether this would be particularly useful in the group of patients in whom venesection is contraindicated.

For peer review only - http://bmjopen.bmj.com/site/about/guidelines.xhtml

Anglia Ruskin University | Postgraduate Medical Institute **Clinical Trials Unit**

1.2 Rationale and Risks/Benefits

This study proposes to test the use of whole, fresh, autologous blood as a treatment for severe dry eye disease. The blood can be obtained from a cleaned finger, pricked by the patient using a diabetic lancet and administered immediately to the dry eye. This allows the delivery of not only most of the aforementioned beneficial components of tears but additional growth factors and proteins, fresh and unprocessed, which can help heal the ocular surface. If validated, this may replace current autologous serum practice and its ease of use, vastly reduced cost with greater convenience may mean that it could be extended to other ocular surface diseases.

The proposing team have completed an exploratory study on the use of finger-prick autologous blood (FAB) for persistent epithelial defects and severe dry eye disease and preliminary results indicate improvement with no adverse events reported (21). The exploratory study included 16 patients with a diagnosis of severe to moderate dry eye syndrome and used the FAB method for treatment. The findings of the study demonstrated mean improvements in visual acuity, Oxford corneal staining grade, tear breakup time, Schirmer's test and dry eye disease questionnaire score. The response rate from participants was good with only a single patient who met the inclusion criteria not wishing to participate in the trial. Both the amount of staining (indicating inflammation and ocular surface damage) and their DED questionnaire scores (indicating severity of their symptoms and impact on quality of life) showed mean improvement which reached statistical significance. A search of clinical databases (Medline, CINAHL and AMED) including on-going trials on the UK CRN portfolio database and related websites (www.controlled-trials.com) did not identified any studies using FAB as a treatment for dry eye disease.

For peer review only - http://bmjopen.bmj.com/site/about/guidelines.xhtml

Anglia Ruskin University | Postgraduate Medical Institute **Clinical Trials Unit**

2 Trial Objectives and Design

2.1 Primary Objective and Endpoints

The primary objective is to determine the feasibility of a definitive randomised controlled trial (RCT) to evaluate effectiveness of the use of fresh autologous blood (FAB) compared to conventional treatment for patients with dry eye syndrome. This will involve specifically assessing the following endpoints:

i. Number of eligible patients within the study population and recruitment time frame.
ii. Percentage of eligible patients accepted for randomisation.
iii. Patient compliance with trial protocol, measured as per self-reported adherence to intervention.
iv. Percentage of patients completing study.

2.2 Secondary Objective and Endpoints

The secondary objectives are:

- To determine the effectiveness of the trial intervention and further explore the acceptability of the study design.
- To explore the feasibility of collecting resource use and quality of life data, to inform the design of the health economics component of a future definitive trial.

The secondary end points, which have not been powered for within the feasibility study, include:

For peer review only - http://bmjopen.bmj.com/site/about/guidelines.xhtml

Anglia Ruskin University | Postgraduate Medical Institute Clinical Trials Unit

Outcome Measure	Endpoint
Reduction in corneal inflammation as indicated by staining on front of the eye	Oxford Corneal Staining Guide
Patient pain and symptoms scores	OSDI score
Improvement in objective signs of dry eye disease as indicated by visual acuity	Schirmer's test, tear breakup time, lower and/or upper tear menisci height measurement
Willingness for patients to be randomised and acceptability of the intervention	Structured qualitative interviews
Impact on patients' quality of life	EQ-5D-5L score
Cost to the NHS and patient	Use of additional NHS services and privately purchased over the counter treatments related to dry eyes disease
Safety measure of pressure within eye	Intra ocular pressure (IOP) score

2.3 Trial Design

Single-blind two-arm feasibility randomised controlled trial of FAB with conventional treatment versus conventional therapy alone for dry eye disease, including a qualitative process evaluation.

- ***Arm A – FAB plus conventional treatment***

The patients will use FAB therapy alongside conventional therapy (artificial tears, ciclosporin drops and punctal plugs/cautery) as recommended by their treating ophthalmologist. A fingertip of the hand will be wiped with an alcohol steret and self-pricked using a standard diabetic lancet. The drop of blood is produced as normal and applied to the lower fornix of the affected eye(s) with the lower lid pulled down slightly by the patient. The blood will be applied four times a day. A fresh finger should be used for each eye. The blood will be wiped away with the alcohol steret. Nails should be kept short and nail varnish should be avoided. FAB should be applied at least 15 minutes after any artificial tears and no drops for at least half an hour afterwards.

For peer review only - http://bmjopen.bmj.com/site/about/guidelines.xhtml

- ***Arm B – Conventional treatment only***

The patients will use conventional therapy (artificial tears, cyclosporin drops and punctal plugs/cautery) as recommended by their treating ophthalmologist.

2.3.1 Setting

The study will take place at NHS sites. The main site will be Bedford Hospital NHS Trust and Moorfields Eye Hospital London. Patients will be recruited from corneal and external eye disease clinics at these sites.

For peer review only

For peer review only - http://bmjopen.bmj.com/site/about/guidelines.xhtml

2.4 Study Scheme Diagram

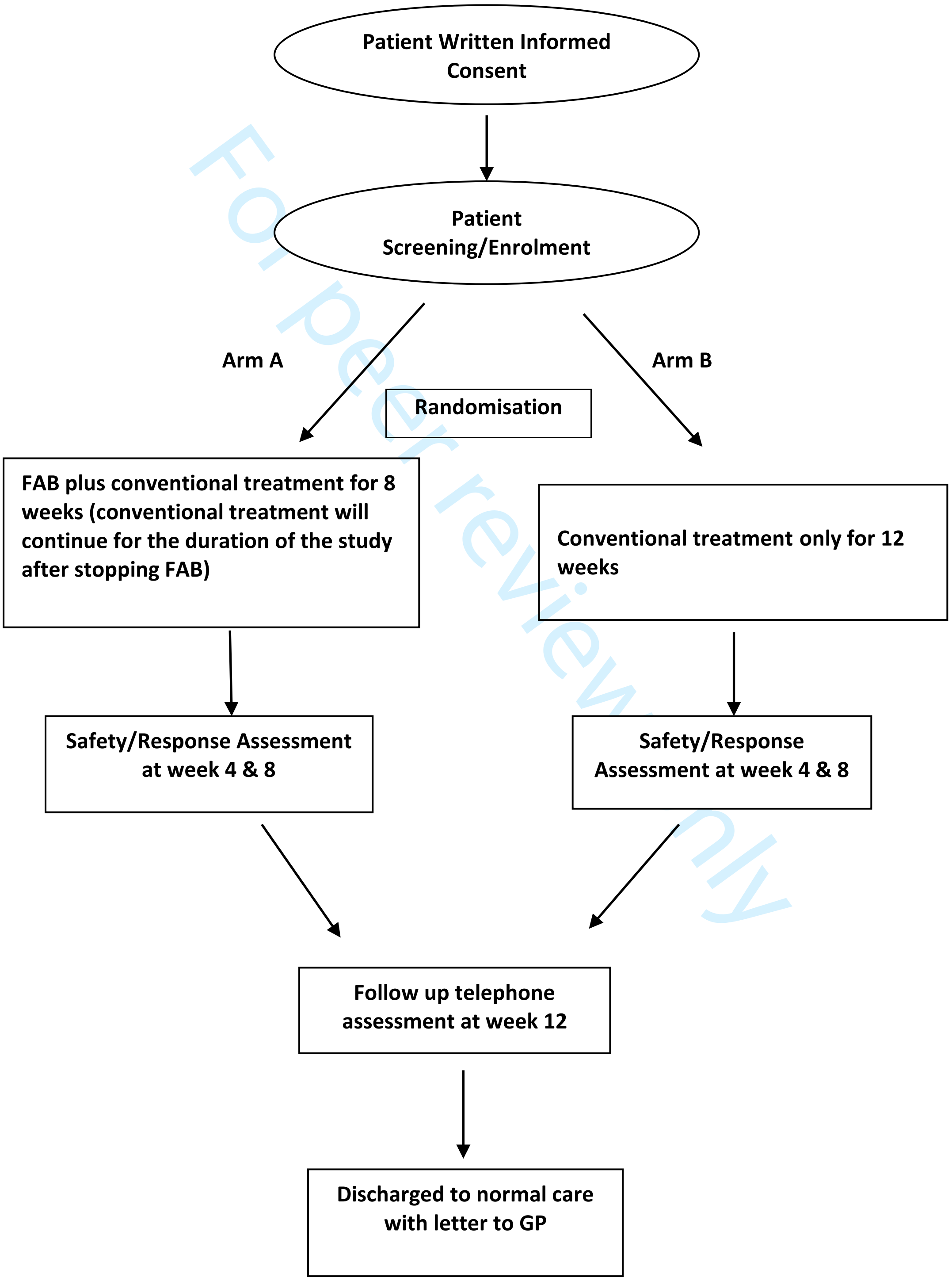

For peer review only - http://bmjopen.bmj.com/site/about/guidelines.xhtml

Anglia Ruskin University | Postgraduate Medical Institute **Clinical Trials Unit**

3 Participant Selection

60 patients (30 in each arm) will be recruited into the study. This allows for an attrition rate of 10%.

3.1 Inclusion Criteria

i. Patient age ≥ 18 years
ii. Severe symptomatic dry eye disease diagnosed by: Ocular surface disease index (OSDI) score of greater than 33; OR Oxford Corneal Staining grade 2 or greater; OR Schirmer's without anaesthesia <5mm at 5 minutes
iii. Patients on artificial tears and/or lubricating drops/gel four times a day
iv. Patient able to give consent
v. Patients able and willing complete the quality of life (QoL) questionnaires required for the study

3.2 Exclusion Criteria

i. Fear of needles
ii. Unable or not willing to carry out repeat finger pricks
iii. Patients with infected finger/s or systemic infection or on systemic antibiotics for infection.
iv. Patients with active ocular infection, active immunological corneal melt, or recurrent corneal erosion.
v. Pregnant or breast feeding women
vi. Previous use of FAB treatment (e.g. from exploratory study)
vii. Systemic illness causing immune system deficiency
viii. Graft versus host disease
ix. Previous use of autologous serum within 3 months
x. Diabetes

For peer review only

For peer review only - http://bmjopen.bmj.com/site/about/guidelines.xhtml

Anglia Ruskin University | Postgraduate Medical Institute Clinical Trials Unit

4 Study Procedures

4.1 Informed Consent Procedures

It is the responsibility of the Investigator, or appropriately GCP trained person delegated by the Investigator as documented in the site delegation log, to obtain written informed consent from each participant prior to any participation/study specific procedures. This should follow adequate explanation of the aims, methods, anticipated benefits and potential hazards of the study. The participants will be given ample time to consider giving their consent for the study. For this study a minimum of 24 hours will be given during which the consenting team will be contactable over the phone to answer any questions. The date that the Participant Information Sheet (PIS) is given to the participant must be documented within the patient's notes to confirm that sufficient time was given. The Investigator (or other qualified person) will explain to the potential participant that they are free to refuse any involvement within the study or alternatively withdraw their consent at any point during the study and for any reason.

If there is any further safety information which may result in significant changes in the risk/benefit analysis, the PIS and Informed Consent Form (ICF) will be reviewed and updated accordingly. All participants that are actively enrolled on the study will be informed of the updated information and given a revised copy of the PIS/ICF in order to confirm their wish to continue on the study.

4.2 Screening Procedures

Patients will have undergone the below procedures as per standard of care which will in turn feed in to the investigator for their review prior to approaching the potential participant.

- Ocular surface disease index (OSDI) score of greater than 33; OR
- Oxford Corneal Staining grade 2 or greater; OR
- Schirmer's without anaesthesia <5mm at 5 minutes

The results of these which will be reviewed by investigator prior to approaching the potential participant.

For peer review only - http://bmjopen.bmj.com/site/about/guidelines.xhtml

4.3 Randomisation Procedures

Randomisation will be carried out by the Anglia Ruskin Clinical Trials Unit (ARCTU) using the SEALED ENVELOPE randomisation service. SEALED ENVELOPE is a randomisation and online database service developed for clinical trial services. It is an internet based system and will be set for this study by ARCTU in accordance with the protocol. Enrolled patients will undergo 1:1 block randomisation to Arm A or B. The system stores the pre-determined sequence of randomisation and this list is visible to neither the investigator nor ARCTU staff. Once a patient has consented to take part in the trial, they will be randomly allocated to either Arm A to receive FAB and convention therapy or Arm B to receive conventional therapy only. The Research Nurse or Fellow or designated staff will log on to a web browser application and enter the patient's eligibility factors into the system. The treatment allocation will be sent to the research team who will make the necessary arrangements for the patient's treatment plan.

The treating ophthalmologist in clinic will prescribe and counsel all patients on the correct technique for conventional treatment. The unblinded research nurses will provide additional training to the patients on the method of delivering FAB, including the use of a training video. Patients will be advised by the research team to inform their ophthalmologist if they have been started on any new treatment during the trial period by other clinicians or themselves over the counter.

4.4 Baseline Procedures

- Patients will undergo the following at baseline:
- Physical examination
- BP and pulse
- Visual acuity test
- Lower and/or upper tear menisci test on optical coherence tomography (OCT, where available)
- Tear breakup time (TBUT)
- Intraocular pressure (IOP)
- Schirmer's test
- Oxford corneal staining grade
- Patient pain and symptom score (OSDI score)
- Lissamine green stain assessment (if available)
- Anterior segment colour photography and cobalt blue fluorescein eye stain photography (if available)
- Anterior segment optical coherence topography (OCT) (if available)
- MMP-9 tear swap (if available)

For peer review only - http://bmjopen.bmj.com/site/about/guidelines.xhtml

Anglia Ruskin University | Postgraduate Medical Institute **Clinical Trials Unit**

4.5 Schedule of Assessment

**Procedures**	**Screening (as per standard of care)**	**Baseline Visit (Day 1)**	**Week 4 visit (+/- 7days**	**Week 8 visit (+/- 7days)**	**Week 12 follow up (Telephone, +/- 7days)**
Informed consent		x			
Demographics		x			
Medical history		x			
Physical examination		x	x	x	
BP and pulse		x	x	x	
Current medical conditions		x	x	x	
Concomitant medications		x	x	x	
Eligibility assessment	x				
Randomisation		x			
Visit dates and appointment		x	x	x	
FAB treatment (Group A only)		x	x	x	
**Treatment and Follow up Phase**					
Completion of QoL questionnaire (EQ-5D-5L)		x	x	x	x
Completion of Health resource questionnaire			x	x	x
Review treatment diary			x	x	
Schirmer's test without anaesthesia (if other criteria not met)	x	x	x	x	
Patient pain and symptom score (OSDI score) if other criteria not met	x	x	x	x	x
Visual acuity		x	x	x	
Lissamine green stain assessment (if available)		x	x	x	
Anterior segment colour photography and cobalt blue fluorescein eye stain photography (if available)		x	x	x	
Anterior segment optical coherence topography (OCT) (if available)		x	x	x	
MMp-9 tear swap (if available)		x		x	
Oxford corneal staining grade	x	x	x	x	
Lower and/or upper tear menisci height (if available)		x	x	x	
Tear breakup time		x	x	x	
Qualitative interview				x	

For peer review only - http://bmjopen.bmj.com/site/about/guidelines.xhtml

Anglia Ruskin University | Postgraduate Medical Institute Clinical Trials Unit

4.6 End of Study Definition

The definition of the end of study will be the point where the last patient recruited had the last follow-up visit.

4.7 Participant Withdrawal

Patients will be withdrawn from study based on the following:

- New diagnosis of infection on finger or eye
- Requesting to be withdrawn for other reasons such as inconvenience of increased clinic visits
- Pregnancy after recruitment
- Systemic infection, or systemic antimicrobials for infection.
- If finger sore from repeated prick and patient does not want to use another finger.

Any adverse effects will be reported back to the lead investigator at the site and if necessary patients

For peer review only

For peer review only - http://bmjopen.bmj.com/site/about/guidelines.xhtml

Anglia Ruskin University | Postgraduate Medical Institute Clinical Trials Unit

5 Pharmacovigilance

5.1 General Definitions

5.1.1 Adverse Event (AE)

An AE is any untoward medical occurrence in a participant to whom a medicinal product has been administered, including occurrences which are not necessarily caused by or related to that product. An AE can therefore be any unfavourable and unintended sign (including an abnormal laboratory finding), symptom or disease temporarily associated with study activities.

5.1.2 Serious Adverse Event (SAE)

An SAE fulfils at least one of the following criteria:

- Is fatal – results in death (NOTE: death is an outcome, not an event)
- Is life-threatening
- Requires inpatient hospitalisation or prolongation of existing hospitalisation
- Results in persistent or significant disability/incapacity
- Is a congenital anomaly/birth defect
- Is otherwise considered medically significant by the Investigator

5.2 Investigators Assessment

5.2.1 Seriousness

The Chief/Principal Investigator responsible for the care of the participant, or in his absence an authorised medic within the research team, is responsible for assessing whether the event is serious according to the definitions given in section 5.1.

5.2.2 Causality

The Investigator must assess the causality of all serious adverse events in relation to the trial treatment according to the definition given

5.2.3 Expectedness

The investigator must assess the expectedness of all SAEs according to the definition given. If the SAE is unexpected and related, then it needs immediate reporting.

For peer review only - http://bmjopen.bmj.com/site/about/guidelines.xhtml

Anglia Ruskin University | Postgraduate Medical Institute **Clinical Trials Unit**

5.2.4 **Severity**

The Investigator must assess the severity of the event according to the following terms and assessments. The intensity of an event should not be confused with the term "serious" which is a regulatory definition based on participant/event outcome criteria.

Mild: Some discomfort noted but without disruption of daily life
Moderate: Discomfort enough to affect/reduce normal activity
Severe: Complete inability to perform daily activities and lead a normal life

5.3 Notification and reporting Adverse Events or Reactions

If the AE is not defined as SERIOUS, the AE is recorded in the study file and the participant is followed up by the research team. The AE is documented in the participants' medical notes (where appropriate) and the CRF.

5.4 Notification and Reporting of Serious Adverse Events

Serious Adverse Event (SAEs) that are considered to be 'related' and 'unexpected' are to be reported to the sponsor within 24 hours of learning of the event and to the Main REC within 15 days in line with the required timeframe. For further guidance on this matter, please refer to Appendix.

5.5 Urgent Safety Measures

The CI may take urgent safety measures to ensure the safety and protection of the clinical trial participants from any immediate hazard to their health and safety, in accordance with Regulation 30. The measures should be taken immediately. In this instance, the approval of the Licensing Authority Approval prior to implementing these safety measures is not required. However, it is the responsibility of the CI to inform the sponsor and Main Research Ethics Committee (via telephone) of this event **immediately**.

The CI has an obligation to inform both the Main Ethics Committee **in writing within 3 days**, in the form of a substantial amendment. The sponsor must be sent a copy of the

For peer review only - http://bmjopen.bmj.com/site/about/guidelines.xhtml

correspondence with regards to this matter. For further guidance on this matter, please refer to Appendix.

5.6 Annual Safety Reporting

The CI will send the Annual Progress Report to the main REC using the NRES template (the anniversary date is the date on the MREC "favourable opinion" letter from the MREC) and to the sponsor. Please see appendix for further information.

5.7 Overview of the Safety Reporting Process/Pharmacoviligance responsibilities

The CI/PI has the overall pharmacovigilance oversight responsibility. The CI/PI has a duty to ensure that pharmacovigilance monitoring and reporting is conducted in accordance with the sponsor's requirements.

For peer review only

For peer review only - http://bmjopen.bmj.com/site/about/guidelines.xhtml

6 Statistical Considerations

A detailed data validation will examine completeness, existence and accuracy of collected data to assess data quality and identify missing and conflicting data. To distinguish missing value mechanisms visualisation methods will be used [R package; Visualization and Imputation of Missing (VIM)] (22). Assumptions based on data Missing At Random (MAR)— that is, that a value is missing depends only on observed values and not on unobserved values (23)— will be imputed using multivariate imputations procedures [(R package; Multivariate Imputation by Chained Equations (MICE) (24)]. The statistical analysis will be performed on an intention-to-treat basis and per-protocol and will be reported according to 2010 CONSORT guidelines (25). All statistical analyses will be completed using SPSS Statistics V.22.0 (26). A p-value of <.05 will be accepted as statistical significance.

Conforming to recommendations for feasibility studies, data analysis will be primarily descriptive, with means, standard deviations and frequency counts calculated for all variables of interest. Exploratory efficacy analysis will compare the primary outcome variables derived from the data collected at 8 weeks between the two arms using a marginal general linear model (GLM) with robust standard errors, to allow for clustering by group. Secondary outcomes between the intervention and control groups will be compared at 4 weeks, 8 weeks and 12 weeks (4 weeks post treatment). Safety outcome measures will include IOP rise and any reported infection.

Secondary analyses of the primary outcome, controlling for baseline covariates, will be performed using proportional hazards regression models (27). Analysis of treatment discontinuation adjusted for clinical site, baseline status, and whether the patient is randomized to the FAB or control (conventional treatment only) group will be conducted. Any interaction between baseline status or regimen and treatment will also be assessed. Interactions between significant covariates and treatment effects will be assessed in the context of the proportional hazards models.

For peer review only - http://bmjopen.bmj.com/site/about/guidelines.xhtml

Anglia Ruskin University | Postgraduate Medical Institute Clinical Trials Unit

6.1 Qualitative approach

A nested qualitative approach using in-depth, semi-structured interviews will help us understand the lived experience of people using FAB and factors relating to the ways that clinical departments and healthcare professionals adapt to working with FAB. Topics, questions and probes in the interviews will emerge from three sources: a) the relevant literature; b) our experience of and involvement in clinical ophthalmology; c) consultation with patients. In this feasibility study, we will use a convenience sampling technique for the qualitative work. We will interview all clinicians who administer the intervention in the study and we will aim to interview 10 patients (adjusted for setting, gender and age group representation). Given the resources, we will aim to draw findings from a larger sample in a future study. Interviews will be recorded, transcribed and analysed using the framework analysis. We will structure the analysis of collected data to fit two evaluation frameworks relevant to the successful implementation of FAB:

1) Patient-oriented: Understand the lived experience of patients using FAB with emphasis on intervention acceptability, perceived enablers and barriers for adherence, perceived clarity of advice and guidance and factors relating to the initial uptake of the intervention.
2) Organisation-oriented: Whether and how the use of FAB affected workload and/or workflow; identify process change, adaptation challenges, skills gap; establish treatment fidelity and clinician acceptability.

This phase will work in parallel to the quantitative data collection so that emerging themes can be investigated in later interviews. Even though the feasibility study sample is reasonably limited, we anticipate some useful insights about the use of FAB to occur because of the triangulation between the RCT and the qualitative findings (e.g. reasons for partial adherence).

6.2 Economic Evaluation

Good practice recommendations for cost-effectiveness analyses (28) suggest concentrating on the measurement of large cost drivers, with less focus on resources that are not expected to differ between different treatments. Estimation of cost-effectiveness is therefore an iterative

For peer review only - http://bmjopen.bmj.com/site/about/guidelines.xhtml

Anglia Ruskin University | Postgraduate Medical Institute Clinical Trials Unit

process and by including a health economic component in a feasibility it is possible to consider how the methods might be refined in any future definitive study. In order to estimate costs, informed by previous data collection instruments (Database of Instruments for Resource Use Measurement: http://www.dirum.org/) and NICE guidance (29), a self-report questionnaire will be devised. This will capture the use of NHS services and privately purchased over the counter treatments such as OPTREX. For benefits, the EQ-5D-5L (30) will be used to measure quality of life, enabling QALY (Quality Adjusted Life Year) scores to be calculated. The main purpose of the economic analysis is to inform the decision regarding how and what cost and effect data would be collected within a more definitive study. in order to inform this decision, we will estimate completion rates and seek to identify big cost drivers.

For peer review only - http://bmjopen.bmj.com/site/about/guidelines.xhtml

Anglia Ruskin University | Postgraduate Medical Institute Clinical Trials Unit

7 Data Handling & Record Keeping

7.1 Confidentiality

The Investigator has a responsibility to ensure that participant anonymity is protected and maintained. They must also ensure that their identities are protected from any unauthorised parties. Information with regards to study participants will be kept confidential and managed in accordance with the Data Protection Act, NHS Caldicott Guardian, The Research Governance Framework for Health and Social Care and Research Ethics Committee Approval.

7.2 Study Documents

- A signed protocol and any subsequent amendments
- Investigator's Brochure
- Sponsor Self-Monitoring template for the trial team to complete on a regular basis as detailed by the Monitoring section
- Current/Superseded Participant Information Sheets (as applicable)
- Current/Superseded Consent Forms (as applicable)
- Indemnity documentation from sponsor
- Conditions of Sponsorship from sponsor
- Conditional/Final R&D Approval
- Signed site agreement
- Ethics submissions/approvals/correspondence
- CVs of CI and site staff
- Delegation log
- Staff training log
- Site signature log
- Participant identification log
- Screening log
- Enrolment log
- Monitoring visit log
- Protocol training log
- Correspondence relating to the trial
- Communication Plan between the CI/PI and members of the study team
- SAE reporting plan for the study

For peer review only - http://bmjopen.bmj.com/site/about/guidelines.xhtml

Anglia Ruskin University | Postgraduate Medical Institute **Clinical Trials Unit**

7.3 Case Report Form

Project data collection will be managed by the Clinical Trials Unit Data Manager. The responsibility for data entry rests with the Research Nurse or designated staff who is supported by the Investigator. ARCTU uses an online data management system called MACRO to design and manage eCRFs (Electronic Case Report Forms). ARCTU will work together with the sponsor to design and validate the data collection tools so that they are appropriate for this study. Once a patient is enrolled on the study the research team can access these forms remotely through the Internet portal and study data will be entered and captured for the study. All data will be in anonymised form and patients will be identifiable only by study number. Data will be remotely monitored by ARCTU and discussed at data monitoring committee meetings. Any inconsistencies, validation errors or inaccuracies will be reported to the lead investigator regularly. Once data collection is complete and the data has been validated, a data lock will be performed and analysis will begin.

7.4 Record Retention and Archiving

During the course of research, all records are the responsibility of the Chief Investigator and must be kept in secure conditions. When the research trial is complete, it is a requirement of the Research Governance Framework and Trust Policy that the records are kept for a further 20 years.

7.5 Compliance

The CI will ensure that the trial is conducted in compliance with the principles of the Declaration of Helsinki (1996), and in accordance with all applicable regulatory requirements including but not limited to the Research Governance Framework, Trust and Research Office policies and procedures and any subsequent amendments.

For peer review only - http://bmjopen.bmj.com/site/about/guidelines.xhtml

Anglia Ruskin University | Postgraduate Medical Institute **Clinical Trials Unit**

7.6 Clinical Governance Issues

7.6.1 Ethical Considerations

This protocol and any subsequent amendments, along with any accompanying material provided to the participant in addition to any advertising material will be submitted by the Investigator to an Independent Research Ethics Committee. Written Approval from the Committee will be obtained and subsequently submitted to the trusts Research and Development office to obtain Final R&D approval.

7.7 Quality Control and Quality Assurance

7.7.1 Summary Monitoring Plan

ARCTU will ensure that the project is carried out in accordance with the Research Governance Framework. All research team members will have GCP (Good Clinical Practice) training before the research commences to ensure every aspect from trial design to dissemination is carried out in line with these principals. GCP is an international quality standard that is provided by International Conference on Harmonisation (ICH), an international body that defines standards, which governments can transpose into regulations for clinical trials involving human subjects.

7.7.2 Audit and Inspection

Auditing: Definition "A systematic and independent examination of trial related activities and documents to determine whether the evaluated trial related activities were conducted, and the data were recorded, analysed and accurately reported according to the protocol, sponsor's standard operating procedures (SOPs), Good Clinical Practice (GCP), and the applicable regulatory requirement(s)."

The FAB in DED study may receive an audit by any of the methods listed below:

For peer review only - http://bmjopen.bmj.com/site/about/guidelines.xhtml

- A project may be identified via the risk assessment process.
- An individual investigator or department may request an audit.
- A project may be identified via an allegation of research misconduct or fraud or a suspected breach of regulations.
- Projects may be selected at random. The Department of Health states that Trusts should be auditing a minimum of 10% of all research projects.
- Projects may be randomly selected for audit by an external organisation.

Internal audits will be conducted by a sponsor's representative

7.8 Non-Compliance

Non-compliance can be defined as 'a noted systematic lack of both the CI and the study staff adhering to SOPs/protocol/ICH-GCP, which leads to prolonged collection of deviations, breaches or suspected fraud.'

These non-compliances may be captured from a variety of different sources including monitoring visits, CRFs, communications and updates. The sponsor will maintain a log of the non-compliances to ascertain if there are any trends developing which to be escalated. The sponsor will assess the non-compliances and action a timeframe in which they need to be dealt with. Each action will be given a different timeframe dependant on the severity. If the actions are not dealt with accordingly, the sponsor will agree an appropriate action, including an on-site audit.

For peer review only - http://bmjopen.bmj.com/site/about/guidelines.xhtml

Anglia Ruskin University | Postgraduate Medical Institute **Clinical Trials Unit**

8 Trial Committees

Trial Management Group (TMG)

A Trial Management Group (TMG) has been formed comprising the Chief Investigator, other lead investigators (clinical and non-clinical) and members of the Data Centres. The TMG will be responsible for the day-to-day running and management of the trial and will meet at least 3 times a year by teleconference.

Trial Steering Committee (TSC)

The Trial Steering Committee (TSC) has membership from TMG plus independent members, including the chair. The role of the TSC is to provide overall supervision for the trial and provide advice through its independent Chairman. The ultimate decision for the continuation of the trial lies with the TSC.

Independent Data Monitoring Committee (IDMC)

The Independent Data Monitoring Committee (IDMC) is the only group who sees the confidential, accumulating data to the trial. Reports to the IDMC will be produced by the trial statistician. The IDMC will meet within 6 months of the trial opening with the frequency of meetings dictated by the IDMC. The IDMC will consider the report for the interim analysis based on the first 30 patients and will decide, keeping in mind the rules described in the statistics section, whether to recommend that the study should continue or be closed. The primary outcome measure for the study addresses toxicity and so it aligns with the IDMC's precautionary role in patient safety. The details of the interim analysis will remain confidential to the IDMC until the study is closed.

For peer review only - http://bmjopen.bmj.com/site/about/guidelines.xhtml

9 Publication Policy

Data obtained from this study will be published in a suitable peer-review journal and also presented at international ophthalmic conferences including American Academy of Ophthalmology, Royal College of Ophthalmology annual congress, Association for Research and Vision and Ophthalmology, and the European Society of Cataract and Refractive Surgery. Information would be provided to patient groups and charities such as the Sjogren's society and the Royal National Institute of Blind People.

For peer review only - http://bmjopen.bmj.com/site/about/guidelines.xhtml

Anglia Ruskin University | Postgraduate Medical Institute Clinical Trials Unit

References

1. Hom MM, Nguyen A, Bielory L. Allergic conjuctivitis and dry eye syndrome. Ann Allergy Asthma Immunol Off Publ Am Coll Allergy Asthma Immunol. 2012;108:163–6.

2. Schiffman RM, Walt JG, Jacobsen G, Doyle JJ, Lebovics G, Sumner W. Utility assessment among patients with dry eye disease. Ophthalmology. 2003;110(7):1412–9.

3. National Eye Research Centre. Eye research overview [Internet]. 2016. Available from: https://www.nerc-charity.org.uk/our-eye-research

4. Kymionis GD, Bouzoukis DI, Diakonis VF, Siganos C. Treatment of chronic dry eye: focus on cyclosporine. Clin Ophthalmol Auckl NZ. 2008 Dec;2(4):829–36.

5. Diebold Y, Herreras JM, Callejo S, Argueso P, Calonge M. Carbomer- Versus Cellulose-Based Artificial-Tear Formulations: Morphologic and Toxicologic Effects on a Corneal Cell Line. Cornea. 1998;17(4):433.

6. Yokoi N, Yamada H, Mizukusa Y, Bron AJ, Tiffany JM, Kato T, et al. Rheology of Tear Film Lipid Layer Spread in Normal and Aqueous Tear–Deficient Dry Eyes. Invest Ophthalmol Vis Sci. 2008 Dec 1;49(12):5319–24.

7. Pfister R, Burstein N. The effects of opthalmic drugs, vehicles and preservatives on corneal epithelium: a scanning electron microscope study. Invest Ophthalmol Vis Sci. 1976;15(4):246–59.

8. Fox R, Chan R, Michelson J, Belmont J, Michelson P. Beneficial effect of artificial tears made with artificial tears made with autologous serum in patients with keratoconjunctivitis sicca. Arthritis Rheumatol. 1984;29:577–83.

9. Poon AC, Geerling G, Dart JKG, Fraenkel GE, Daniels JT. Autologous serum eyedrops for dry eyes and epithelial defects: clinical and in vitro toxicity studies. Br J Ophthalmol. 2001 Oct 1;85(10):1188.

10. Tsubota K, Goto E, Fujuta H, Ono M, Inuoue H, Saito I. Treatment of dry eye by autologous serum application in Sjogren’s Syndrome. Br J Ophthalmol. 1999;83:390–5.

11. Pan Q, Angelina A, Zambrano A, Marrone M, Stark WJ, Heflin T, et al. Autologous serum eye drops for dry eye. Cochrane Database Syst Rev [Internet]. 2013;(8). Available from: http://dx.doi.org/10.1002/14651858.CD009327.pub2

12. Biswas S, Zaheer I, Monsalve B, Diamond JP. Compression sutures with autologous blood injection for leaking trabeculectomy blebs. Br J Ophthalmol. 2009 Mar 25;93(4):549.

13. Burnstein A, WuDunn D, Ishii Y, Jonescu-Cuypers C, Cantor LB. Autologous blood injection for late-onset filtering bleb leak. Am J Ophthalmol. 2001 Jul 1;132(1):36–40.

For peer review only - http://bmjopen.bmj.com/site/about/guidelines.xhtml

Anglia Ruskin University | Postgraduate Medical Institute **Clinical Trials Unit**

14. Dinah C, Bhachech B, Ghosh G. Long-term success with autologous blood injection for leaking trabeculectomy blebs. Br J Ophthalmol. 2010 Mar 9;94(3):392.

15. Haynes WL, Alward WLM. Combination of Autologous Blood Injection and Bleb Compression Sutures to Treat Hypotony Maculopathy. J Glaucoma [Internet]. 1999;8(6). Available from: http://journals.lww.com/glaucomajournal/Fulltext/1999/12000/Combination_of_Autologous_Blood_Injection_and_Bleb.8.aspx

16. de Wit D, Athanasiadis I, Sharma A, Moore J. Sutureless and glue-free conjunctival autograft in pterygium surgery: a case series. Eye. 2010 Sep;24(9):1474–7.

17. Chuang L-H, Wang N-K, Yeung L, Chen Y-P, Hwang Y-S, Wu W-C, et al. Use of autologous whole blood during internal limiting membrane peeling and macular hole surgery is protective for indocyanine green toxicity. Cutan Ocul Toxicol. 2010 Jun 1;29(2):98–104.

18. Lai C-C, Hwang Y-S, Liu L, Chen K-J, Wu W-C, Chuang L-H, et al. Blood-Assisted Internal Limiting Membrane Peeling for Macular Hole Repair. Ophthalmology. 2009 Aug 1;116(8):1525–30.

19. Lai C-C, Wu W-C, Chuang L-H, Yeung L, Chen T-L, Lin K-K. Prevention of Indocyanine Green Toxicity on Retinal Pigment Epithelium with Whole Blood in Stain-Assisted Macular Hole Surgery. Ophthalmology. 2005 Aug 1;112(8):1409–14.

20. McCannel CA. Heparinized Autologous Blood for Visualization of the Internal Limiting Membrane During Macular Hole Surgery. Retina [Internet]. 2008;28(2). Available from: http://journals.lww.com/retinajournal/Fulltext/2008/02000/Heparinized_Autologous_Blood_for_Visualization_of.22.aspx

21. Balal S, Sharma A, Nesaratnam N, Than J, Wawrzynski J, Patel A. Fingerprick autologous blood: a novel treatment for dry eye syndrome and persistent epithelial defects In: Cornea Medical II [Internet]. Copehagen: European Society of Cataract & Refractive Surgeons; 2016. Available from: http://www.escrs.org/abstracts/details.asp?confid=22&sessid=819&type=Free&paperid=27426

22. VIM. Visualization and Imputation of Missing Values [Internet]. 2017. Available from: [http://cran.r-project.org/package=VIM]

23. Schafer JL, John W. Graham. Missing Data: Our View of the State of the Art. Psychol Methods. 2002;7(2):147–77.

24. Azur MJ, Stuart EA, Frangakis C, Leaf PJ. Multiple imputation by chained equations: what is it and how does it work? Int J Methods Psychiatr Res. 2011;20(1):40–9.

25. Schulz KF, Altman DG, Moher D. CONSORT 2010 Statement: updated guidelines for reporting parallel group randomised trials. BMJ [Internet]. 2010 Mar 24;340. Available from: http://www.bmj.com/content/340/bmj.c332.abstract

For peer review only - http://bmjopen.bmj.com/site/about/guidelines.xhtml

26. IBM. IBM SPSS Statistics for Windows, Version 22.0. Armounk, NY: IBM Corp.; 2012.

27. Cox D. Regression models and life-tables. J R Stat Soc. 1972;(34):187–220.

28. Ramsey SD, Willke RJ, Glick H, Reed SD, Augustovski F, Jonsson B, et al. Cost-Effectiveness Analysis Alongside Clinical Trials II—An ISPOR Good Research Practices Task Force Report. Value Health. 2015 Mar 1;18(2):161–72.

29. National Institute for Health and Clinical Excellence. NICE Guide to the methods of technology appraisal 2013. NICE; 2013.

30. Herdman M, Gudex C, Lloyd A, Janssen M, Kind P, Parkin D, et al. Development and preliminary testing of the new five-level version of EQ-5D (EQ-5D-5L). Qual Life Res. 2011 Dec 1;20(10):1727–36.

For peer review only

For peer review only - http://bmjopen.bmj.com/site/about/guidelines.xhtml

Anglia Ruskin University | Postgraduate Medical Institute **Clinical Trials Unit**

Appendix

	Who	When	How	To Whom
**SAE**	Chief Investigator	-Report to Sponsor within 24 hours of learning of the event<br><br>-Report to the MREC within 15 days of learning of the event	SAE Report form for Non-CTIMPs, available from NRES website.	Sponsor and MREC
**Urgent Safety Measures**	Chief Investigator	Contact the Sponsor and MREC Immediately<br><br>Within 3 days	By phone<br><br>Substantial amendment form giving notice in writing setting out the reasons for the urgent safety measures and the plan for future action.	Main REC and Sponsor<br><br>Main REC with a copy also sent to the sponsor. The MREC will acknowledge this within 30 days of receipt.
**Progress Reports**	Chief Investigator	Annually (starting 12 months after the date of favourable opinion)	Annual Progress Report Form (non-CTIMPs) available from the NRES website	Main REC
**Declaration of the conclusion or early termination of the study**	Chief Investigator	Within 90 days (conclusion)<br><br>Within 15 days (early termination)<br>*The end of study should be defined in the protocol*	End of Study Declaration form available from the NRES website	Main REC with a copy to be sent to the sponsor
**Summary of final Report**	Chief Investigator	Within one year of conclusion of the Research	No Standard Format However, the following Information should be included:-<br>Where the study has met its objectives, the main findings and arrangements for publication or dissemination including feedback to participants	Main REC with a copy to be sent to the sponsor

For peer review only - http://bmjopen.bmj.com/site/about/guidelines.xhtml

Reporting checklist for protocol of a clinical trial.

Based on the SPIRIT guidelines.

Instructions to authors

Complete this checklist by entering the page numbers from your manuscript where readers will find each of the items listed below.

Your article may not currently address all the items on the checklist. Please modify your text to include the missing information. If you are certain that an item does not apply, please write "n/a" and provide a short explanation.

Upload your completed checklist as an extra file when you submit to a journal.

In your methods section, say that you used the SPIRIT reporting guidelines, and cite them as:

Chan A-W, Tetzlaff JM, Altman DG, Laupacis A, Gøtzsche PC, Krleža-Jerić K, Hróbjartsson A, Mann H, Dickersin K, Berlin J, Doré C, Parulekar W, Summerskill W, Groves T, Schulz K, Sox H, Rockhold FW, Rennie D, Moher D. SPIRIT 2013 Statement: Defining standard protocol items for clinical trials. Ann Intern Med. 2013;158(3):200-207

		Reporting Item	Page Number
Title	#1	Descriptive title identifying the study design, population, interventions, and, if applicable, trial acronym	1
Trial registration	#2a	Trial identifier and registry name. If not yet registered, name of intended registry	3
Trial registration: data set	#2b	All items from the World Health Organization Trial Registration Data Set	n/a
Protocol version	#3	Date and version identifier	n/a
Funding	#4	Sources and types of financial, material, and other support	18
Roles and responsibilities: contributorship	#5a	Names, affiliations, and roles of protocol contributors	1 & 2
Roles and responsibilities:	#5b	Name and contact information for the trial sponsor	2

For peer review only

sponsor contact information			
Roles and responsibilities: sponsor and funder	#5c	Role of study sponsor and funders, if any, in study design; collection, management, analysis, and interpretation of data; writing of the report; and the decision to submit the report for publication, including whether they will have ultimate authority over any of these activities	n/a
Roles and responsibilities: committees	#5d	Composition, roles, and responsibilities of the coordinating centre, steering committee, endpoint adjudication committee, data management team, and other individuals or groups overseeing the trial, if applicable (see Item 21a for data monitoring committee)	n/a
Background and rationale	#6a	Description of research question and justification for undertaking the trial, including summary of relevant studies (published and unpublished) examining benefits and harms for each intervention	5, 6
Background and rationale: choice of comparators	#6b	Explanation for choice of comparators	7
Objectives	#7	Specific objectives or hypotheses	7
Trial design	#8	Description of trial design including type of trial (eg, parallel group, crossover, factorial, single group), allocation ratio, and framework (eg, superiority, equivalence, non-inferiority, exploratory)	8
Study setting	#9	Description of study settings (eg, community clinic, academic hospital) and list of countries where data will be collected. Reference to where list of study sites can be obtained	8
Eligibility criteria	#10	Inclusion and exclusion criteria for participants. If applicable, eligibility criteria for study centres and individuals who will perform the interventions (eg, surgeons, psychotherapists)	8
Interventions: description	#11a	Interventions for each group with sufficient detail to allow replication, including how and when they will be administered	9

For peer review only

Interventions: modifications	#11b	Criteria for discontinuing or modifying allocated interventions for a given trial participant (eg, drug dose change in response to harms, participant request, or improving / worsening disease)	n/a
Interventions: adherance	#11c	Strategies to improve adherence to intervention protocols, and any procedures for monitoring adherence (eg, drug tablet return; laboratory tests)	n/a
Interventions: concomitant care	#11d	Relevant concomitant care and interventions that are permitted or prohibited during the trial	9
Outcomes	#12	Primary, secondary, and other outcomes, including the specific measurement variable (eg, systolic blood pressure), analysis metric (eg, change from baseline, final value, time to event), method of aggregation (eg, median, proportion), and time point for each outcome. Explanation of the clinical relevance of chosen efficacy and harm outcomes is strongly recommended	7
Participant timeline	#13	Time schedule of enrolment, interventions (including any run-ins and washouts), assessments, and visits for participants. A schematic diagram is highly recommended (see Figure)	
Sample size	#14	Estimated number of participants needed to achieve study objectives and how it was determined, including clinical and statistical assumptions supporting any sample size calculations	8
Recruitment	#15	Strategies for achieving adequate participant enrolment to reach target sample size	8
Allocation: sequence generation	#16a	Method of generating the allocation sequence (eg, computer-generated random numbers), and list of any factors for stratification. To reduce predictability of a random sequence, details of any planned restriction (eg, blocking) should be provided in a separate document that is unavailable to those who enrol participants or assign interventions	12
Allocation concealment	#16b	Mechanism of implementing the allocation sequence (eg, central telephone; sequentially numbered, opaque, sealed	12

For peer review only

mechanism		envelopes), describing any steps to conceal the sequence until interventions are assigned	
Allocation: implementation	#16c	Who will generate the allocation sequence, who will enrol participants, and who will assign participants to interventions	n/a
Blinding (masking)	#17a	Who will be blinded after assignment to interventions (eg, trial participants, care providers, outcome assessors, data analysts), and how	
Blinding (masking): emergency unblinding	#17b	If blinded, circumstances under which unblinding is permissible, and procedure for revealing a participant's allocated intervention during the trial	
Data collection plan	#18a	Plans for assessment and collection of outcome, baseline, and other trial data, including any related processes to promote data quality (eg, duplicate measurements, training of assessors) and a description of study instruments (eg, questionnaires, laboratory tests) along with their reliability and validity, if known. Reference to where data collection forms can be found, if not in the protocol	14
Data collection plan: retention	#18b	Plans to promote participant retention and complete follow-up, including list of any outcome data to be collected for participants who discontinue or deviate from intervention protocols	14
Data management	#19	Plans for data entry, coding, security, and storage, including any related processes to promote data quality (eg, double data entry; range checks for data values). Reference to where details of data management procedures can be found, if not in the protocol	14
Statistics: outcomes	#20a	Statistical methods for analysing primary and secondary outcomes. Reference to where other details of the statistical analysis plan can be found, if not in the protocol	10, 11
Statistics: additional analyses	#20b	Methods for any additional analyses (eg, subgroup and adjusted analyses)	n/a
Statistics: analysis population and missing data	#20c	Definition of analysis population relating to protocol non-adherence (eg, as randomised analysis), and any statistical methods to handle missing data (eg, multiple imputation)	10

For peer review only

Data monitoring: formal committee	#21a	Composition of data monitoring committee (DMC); summary of its role and reporting structure; statement of whether it is independent from the sponsor and competing interests; and reference to where further details about its charter can be found, if not in the protocol. Alternatively, an explanation of why a DMC is not needed	n/a
Data monitoring: interim analysis	#21b	Description of any interim analyses and stopping guidelines, including who will have access to these interim results and make the final decision to terminate the trial	n/a
Harms	#22	Plans for collecting, assessing, reporting, and managing solicited and spontaneously reported adverse events and other unintended effects of trial interventions or trial conduct	13
Auditing	#23	Frequency and procedures for auditing trial conduct, if any, and whether the process will be independent from investigators and the sponsor	n/a
Research ethics approval	#24	Plans for seeking research ethics committee / institutional review board (REC / IRB) approval	3
Protocol amendments	#25	Plans for communicating important protocol modifications (eg, changes to eligibility criteria, outcomes, analyses) to relevant parties (eg, investigators, REC / IRBs, trial participants, trial registries, journals, regulators)	14
Consent or assent	#26a	Who will obtain informed consent or assent from potential trial participants or authorised surrogates, and how (see Item 32)	12
Consent or assent: ancillary studies	#26b	Additional consent provisions for collection and use of participant data and biological specimens in ancillary studies, if applicable	n/a
Confidentiality	#27	How personal information about potential and enrolled participants will be collected, shared, and maintained in order to protect confidentiality before, during, and after the trial	14
Declaration of interests	#28	Financial and other competing interests for principal investigators for the overall trial and each study site	18
Data access	#29	Statement of who will have access to the final trial dataset,	15

For peer review only

		and disclosure of contractual agreements that limit such access for investigators	
Ancillary and post trial care	#30	Provisions, if any, for ancillary and post-trial care, and for compensation to those who suffer harm from trial participation	n/a
Dissemination policy: trial results	#31a	Plans for investigators and sponsor to communicate trial results to participants, healthcare professionals, the public, and other relevant groups (eg, via publication, reporting in results databases, or other data sharing arrangements), including any publication restrictions	15
Dissemination policy: authorship	#31b	Authorship eligibility guidelines and any intended use of professional writers	n/a
Dissemination policy: reproducible research	#31c	Plans, if any, for granting public access to the full protocol, participant-level dataset, and statistical code	n/a
Informed consent materials	#32	Model consent form and other related documentation given to participants and authorised surrogates	n/a
Biological specimens	#33	Plans for collection, laboratory evaluation, and storage of biological specimens for genetic or molecular analysis in the current trial and for future use in ancillary studies, if applicable	n/a

The SPIRIT checklist is distributed under the terms of the Creative Commons Attribution License CC-BY-ND 3.0. This checklist can be completed online using https://www.goodreports.org/, a tool made by the EQUATOR Network in collaboration with Penelope.ai

For peer review only
