# Supplementary material for: The feasibility of finger prick autologous blood (FAB) as a novel treatment for severe dry eye disease (DED): protocol for a randomised controlled trial
Source: BMJ Open. 2018 Oct 31;8(10):e026770. doi: 10.1136/bmjopen-2018-026770 (PMC6252631; doi:10.1136/bmjopen-2018-026770)
Supplement: Reviewer comments [file bmjopen-2018-026770.reviewer_comments.pdf]

PEER REVIEW HISTORY

BMJ Open publishes all reviews undertaken for accepted manuscripts. Reviewers are asked to complete a checklist review form (**http://bmjopen.bmj.com/site/about/resources/checklist.pdf**) and are provided with free text boxes to elaborate on their assessment. These free text comments are reproduced below.

ARTICLE DETAILS

**TITLE (PROVISIONAL)**	The Feasibility of Fingerprick Autologous Blood (FAB) As a Novel Treatment for Severe Dry Eye Disease (DED): Protocol for a Randomised Controlled Trial
**AUTHORS**	Balal, Shafi; Udoh, Arit; Pappas, Yannis; Cook, Erica; Barton, Garry; Hassan, Ali; Hayden, Karen; Bourne, Rupert; Ahmad, Sajjad; Pardhan, Shahina; Harrison, Michael; Sharma, Benjamin; Wasil, Mohammad; Sharma, Anant

This article was not externally reviewed at BMJ Open. Protocols that have been independently assessed prior to submission to BMJ Open are usually fast-tracked to publication on the grounds that further substantial changes will not be possible. This independent assessment will usually be external, independent review for both a substantial grant award from a non-commercial or government funder and ethics approval.
